# Involving men to improve maternal and newborn health: A systematic review of the effectiveness of interventions

**DOI:** 10.1371/journal.pone.0191620

**Published:** 2018-01-25

**Authors:** Mariam Tokhi, Liz Comrie-Thomson, Jessica Davis, Anayda Portela, Matthew Chersich, Stanley Luchters

**Affiliations:** 1 Burnet Institute, Melbourne, Victoria, Australia; 2 Department of Epidemiology and Preventive Medicine, Monash University, Melbourne, Victoria, Australia; 3 Department of Uro-gynaecology, Faculty of Medicine and Health Sciences, Ghent University, Ghent, Belgium; 4 Department of Maternal, Newborn, Child and Adolescent Health, World Health Organization, Geneva, Switzerland; 5 Wits Reproductive Health and HIV Institute, University of the Witwatersrand, Johannesburg, South Africa; 6 Department of Obstetrics and Gynaecology, International Centre for Reproductive Health (ICRH), Ghent University, Ghent, Belgium; TNO, NETHERLANDS

## Abstract

**Background:**

Emerging evidence and program experience indicate that engaging men in maternal and newborn health can have considerable health benefits for women and children in low- and middle-income countries. Previous reviews have identified male involvement as a promising intervention, but with a complex evidence base and limited direct evidence of effectiveness for mortality and morbidity outcomes.

**Objective:**

To determine the effect of interventions to engage men during pregnancy, childbirth and infancy on mortality and morbidity, as well as effects on mechanisms by which male involvement is hypothesised to influence mortality and morbidity outcomes: home care practices, care-seeking, and couple relationships.

**Methods:**

Using a comprehensive, highly sensitive mapping of maternal health intervention studies conducted in low- and middle-income countries between 2000 and 2012, we identified interventions that have engaged men to improve maternal and newborn health. Primary outcomes were care-seeking for essential services, mortality and morbidity, and home care practices. Secondary outcomes relating to couple relationships were extracted from included studies.

**Results:**

Thirteen studies from nine countries were included. Interventions to engage men were associated with improved antenatal care attendance, skilled birth attendance, facility birth, postpartum care, birth and complications preparedness and maternal nutrition. The impact of interventions on mortality, morbidity and breastfeeding was less clear. Included interventions improved male partner support for women and increased couple communication and joint decision-making, with ambiguous effects on women’s autonomy.

**Conclusion:**

Interventions to engage men in maternal and newborn health can increase care-seeking, improve home care practices, and support more equitable couple communication and decision-making for maternal and newborn health. These findings support engaging men as a health promotion strategy, although evidence gaps remain around effects on mortality and morbidity. Findings also indicate that interventions to increase male involvement should be carefully designed and implemented to mitigate potential harmful effects on couple relationship dynamics.

## Introduction

**Fig 1:**
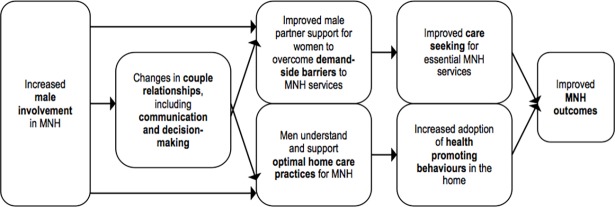
Explanatory model for the effect of male involvement on MNH outcomes.

Men have an important role in maternal and newborn health (MNH) as partners and parents, and can influence behaviours related to MNH within their households and communities [1]. Since the mid-1990s there has been increased recognition of the importance of including men in MNH programs [1–3]. Evidence indicates several mechanisms by which male involvement in MNH can support improved health outcomes (Fig 1). Men can provide substantial practical [4], financial [5] and emotional [6,7] support to women and children to overcome demand-side barriers to accessing health services. Male involvement programs can also contribute to normalising care-seeking within households and communities [8,9]. Men can adopt, and encourage other household members to adopt, health-promoting behaviours at home such as improved nutrition and hygiene practices [10]. Interventions to increase male involvement in MNH have also been linked with changes in couple relationships, such as increased couple communication and equitable decision-making [11], which contribute to improved health and care-seeking outcomes [12,13].

Existing systematic reviews of the effect of male involvement on maternal health [14], MNH [15], and related topics [16–18] have identified male involvement as a promising intervention, but with a complex evidence base and limited evidence of effectiveness for mortality and morbidity outcomes. This review was conducted to comprehensively assess the effectiveness of male involvement in improving MNH in low- and middle-income countries (LMICs), to inform World Health Organization (WHO) health promotion guidelines [2]. To support interpretation of the complex evidence base, the review was designed to capture not only effects on health outcomes, but also effects on key mechanisms by which male involvement is hypothesised to influence these health outcomes: care-seeking, home care practices, and couple relationships. Review findings therefore provide guidance on whether, and how, male involvement can affect MNH outcomes, to assist interpretation and application of the WHO health promotion guidelines that recommend male involvement [2].

## Methods

### Identification of studies

This systematic review was conducted in two stages, described in full elsewhere [2,19,20]. Full protocols for both stages are included as S1–S3 Files. The first stage, a MASCOT/MH-SAR study, used a highly sensitive search strategy to identify, screen and map all maternal health intervention studies conducted in LMICs as defined by the World Bank and published between 2000 and 2012. The mapping focused on health system and community-based interventions for improving maternal health and reducing maternal health inequities. A broad search strategy identified 35,048 unique titles. All of these were screened on title and abstract, 4,172 screened on full text and 2,292 included in the mapping [21].

The second stage of the review sought to answer the question:

What interventions used to increase male involvement have been effective in increasing care-seeking behaviour during pregnancy, for childbirth and after birth for women and newborns and in improving key maternal and newborn health outcomes?

During the MASCOT/MR-SAR mapping, reviewers identified articles addressing this question. Additional studies were identified through expert consultation, reference lists of two published reviews [11,22], and reference lists of included studies. Studies were included if they met the following criteria:

- Study population: women, men, community leaders, communities, and health workers in low and middle income countries;
- Intervention: any intervention intended to increase male involvement during pregnancy, childbirth or after birth;
- Comparison: no intervention for male involvement, or different interventions for male involvement;
- Outcomes: antenatal or postpartum care for women, uptake of essential maternal health services, birth and complication preparedness, birth with a skilled attendant or in a facility, maternal or newborn nutrition, care-seeking for complications or illness, maternal morbidity, and maternal, perinatal or neonatal mortality.

All study designs were included if there was a comparator and empirical data was reported. Studies were excluded where men’s involvement was sought only to promote family planning or the prevention or treatment of sexually transmitted infections. English, French, Japanese, Portuguese and Spanish language studies were included.

### Data extraction and study quality appraisal

Two reviewers extracted data from studies into standard EPPI-reviewer forms. Duplicate extraction was completed for 50% of included articles, with differences checked by a third reviewer. Data was extracted verbatim with the exception of intervention type, which was defined according to emergent categories. Risk of bias was assessed using the Effective Public Health Practice Project (EPHPP) Quality Assessment Tool for Quantitative Studies to score studies on study design, risk of selection bias, control of confounders, blinding, reliability and validity of data collection methods, withdrawals and drop-outs, intervention integrity, and whether intention-to-treat analysis was used [23]. Duplicate scoring was completed for 50% of studies, with differences checked by a third reviewer.

### Synthesis

Studies varied in interventions, study design, use of control groups, data collection methods and outcome measures used. It was therefore determined that meta-analysis was not feasible, and narrative synthesis was completed for each outcome [24,25]. The varying outcome measures also meant it was not feasible to check for publication bias. The approach to narrative synthesis was guided by the explanatory model for the effect of male involvement on MNH outcomes outlined above (Fig 1). Outcomes that had been pre-defined in the review protocol (S3 File) were grouped into four categories: care-seeking for essential MNH services, health promoting home care practices, mortality and morbidity, and couple relationships. Extracted outcome data was imported into tables (S2–S5 Tables) and compared within each category of outcome variables. Outcome data from studies describing the effects of interventions with multiple integrated components, including a male involvement component, was considered separately from outcome data from studies that were specifically designed to assess the effect of a standalone male involvement intervention.

## Results

### Search results

**Fig 2:**
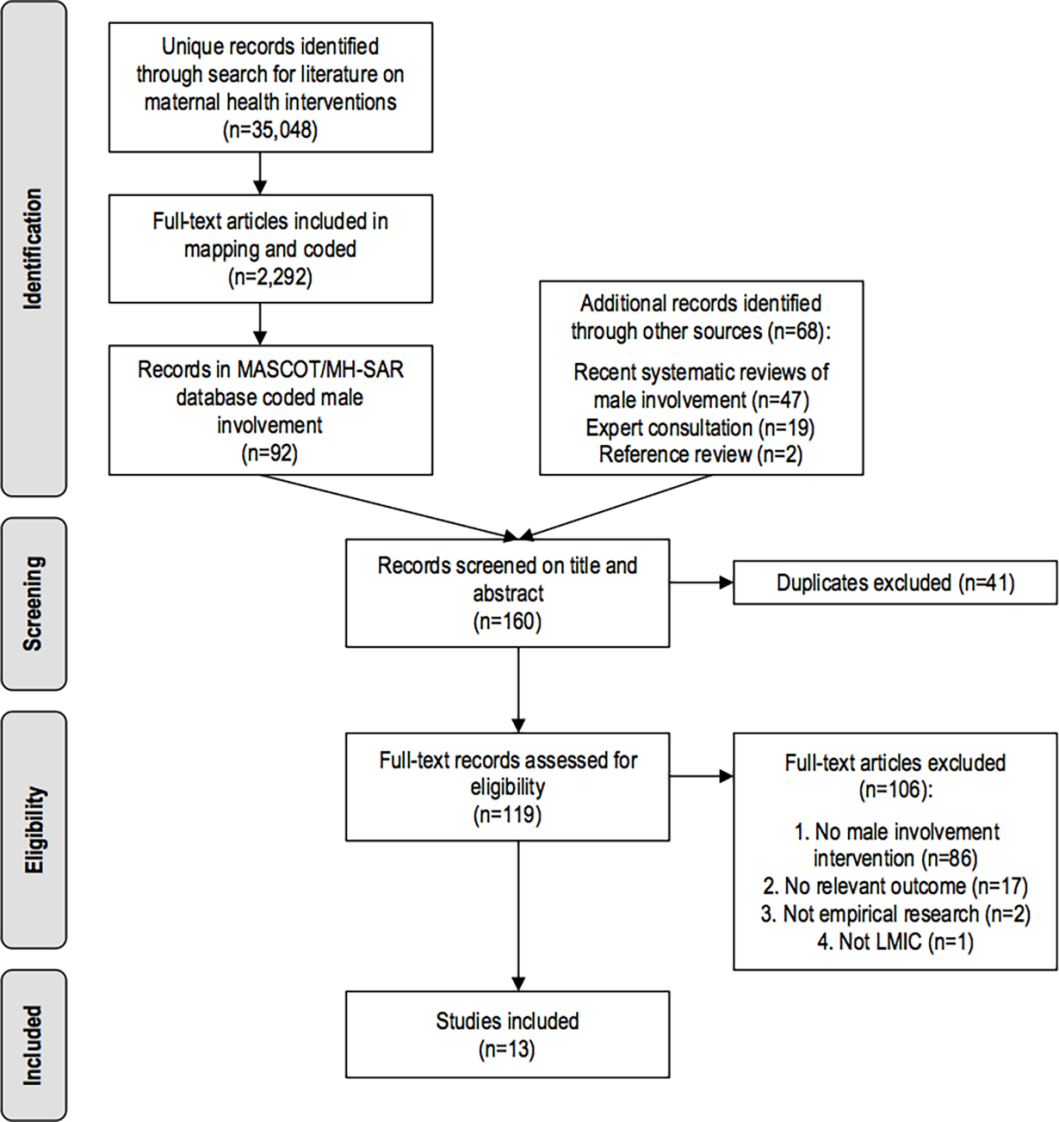
PRISMA flow diagram.

After exclusion of duplicates, 119 articles were screened on full text. Articles were excluded because they did not describe a male involvement intervention (n = 86), did not report a relevant outcome (n = 17), were not empirical research (n = 2) or described a high-income country (n = 1). Thirteen studies were included in the systematic review (Fig 2). Data extracted from included studies is included in S1–S5 Tables.

### Characteristics of included studies

All thirteen studies (Table 1) reported interventions intended to increase the involvement of men in MNH, either as a primary focus of the intervention [26–30] or as one component of a multi-component intervention to improve MNH [31–38]. Eight studies were conducted in South Asia [27,28,30–32,34–36], three in Southern or Eastern Africa [26,33,38] and one each in Indonesia [37] and Turkey [29]. Three studies had experimental designs [26–28] and ten were observational studies [29–38]. All studies were primarily quantitative, with two studies reporting some qualitative findings [29,35].

**Table 1:** Characteristics of included studies.

*Study*	*Study design [39]*	*Intervention*	*Setting*	*Duration*
*Studies designed to assess the effect of a male involvement intervention*				
Kunene 2005 [26]	Cluster randomised controlled trial	Facility-based couples' education at antenatal clinics	South Africa, rural and urban	2001-02 (17 months)
Midhet 2010 [27]	Cluster randomised controlled trial	Community-based education for men and women; training of traditional birth attendants; and community mobilisation to improve referral	Pakistan, rural	1998-2000 (24 months)
Mullany 2007 [28]	Randomised controlled trial	Facility-based couples' education at antenatal clinics at a tertiary hospital	Nepal, urban	2003-04 (8 months)
Sahip 2007 [29]	Cohort analytic	Workplace-based education for expectant fathers	Turkey, urban	Dates not reported (15 months)
Varkey 2004 [30]	Non-equivalent control group	Facility-based education for men and women individually or as a couple at antenatal clinics	India, urban	2001-02 (24 months)
*Studies designed to assess the effect of multiple intervention components*, *including a male involvement intervention*				
Fullerton 2005 [31]	Repeat cross-sectional	Family and community education (home visits and group discussions)	India, rural	2001-02 (13 months)
Hossain 2006 [32]	Quasi-experimental	Family and community education (home visits and group discussions) and improved community-facility linkages	Bangladesh, rural	1999-2001 (26 months)
Mushi 2010 [33]	Before-and-after	Family and community education (home visits and group discussions)	Tanzania, rural	2004-06 (26 months)
Purdin 2009 [34]	Program evaluation using health information system data	Primary healthcare program that included outreach to male partners and community leaders	Pakistan, rural refugee camp	1996-2007 (11 years)
Sinha 2008 [35]	Before-and-after	Family and community education (home visits and public meetings), and improved community-facility linkages	India, rural	2004-06 (18 months)
Sood 2004, Indonesia [37]	Before-and-after with control at endline	Social mobilisation campaign targeting husbands, midwives, and other community members	Indonesia, rural and urban	1999-2003 (52 months)
Sood 2004, Nepal [36]	Before-and-after	Social mobilisation campaign targeting husbands and mothers-in-law	Nepal, rural	2001-03 (22 months)
Turan 2011 [38]	Before-and-after	Community education and training of health care providers	Eritrea, rural	2005-07 (16 months)

### Male involvement interventions

Interventions ranged from five months to 12 years in duration and were delivered through diverse mechanisms including community outreach and education [27,31–38], home visits [31,33,35], facility-based counselling [26,28,30], workplace education programs [29] and mass media social mobilisation campaigns [36,37]. Rationales for involving men also varied between studies, with eight interventions seeking to leverage men’s role as gatekeepers for women’s health [27–31,34,36,37], three interventions aiming to improve men’s access to health services [26,29,30], one parenting intervention designed to support new fathers [38], and five interventions that engaged men as one target group under a broader strategy to increase community involvement in MNH [32,34,35,37,38].

The five studies designed to assess the effect of male involvement alone [26–30] received a moderate rating under the EPHPP quality criteria, while the remaining eight studies, which described multi-component interventions that included a male involvement intervention [31–38], received a weak rating. We have continued this distinction between interventions focused specifically on male involvement and multi-component interventions that include a male involvement intervention in the summary of results below.

### Effect on care-seeking

Eight studies reported on use of antenatal care (ANC) [27,28,33–38]. Of these, two studies specifically designed to assess the effect of male involvement found no change in ANC attendance following facility-based couples’ education in Nepal [27] or community-based education for men and women in Pakistan [28]. The remaining studies, of multi-component interventions that included male involvement, found substantial increases in ANC uptake in Eritrea (OR 17.09, 95% CI 9.85–29.66) [38], Indonesia (83.8% control, 94.4% intervention, p≤0.00) [37], Pakistan (49% baseline, 90% endline, no significance reported) [34] and India (61% baseline, 72.5% post-intervention, p<0.001) [35] but no change following interventions in Nepal [36] or Tanzania [33] that did not supplement education and outreach with activities targeting health providers or facilities.

Nine studies reported on changes in facility birth [27,28,32–38], of which five included data on birth with a skilled attendant either in the home or in a facility [28,32,33,36,37]. Of these, the two studies that specifically assessed the effect of male involvement found no change in facility birth following community-based education in Pakistan [27] and no change in facility birth or birth with a skilled attendant following facility-based education in Nepal [28], although the study in Nepal was conducted among couples attending a tertiary hospital for ANC and facility birth was above 90% in both control and intervention groups [28].

Studies of complex community education or mobilisation interventions targeting men alongside other community members reported increased facility births in Eritrea (OR 26.24, 95% CI 11.42–60.27) [38], India (home births 54.1% baseline, 38.4% post-intervention, p<0.001) [35], Bangladesh (2.4% baseline, 20.5% post-intervention, p<0.01) [32], northern Pakistan (4.8% baseline, 67.2% post-intervention, no significance reported) [34], Indonesia (5.7% control, 11.4% intervention, p≤0.00) [37] and Tanzania (33.3% baseline, 49.8% post-intervention, no significance reported) [33]. In Nepal, one study reported fewer facility births in the intervention arm in the context of a large increase in the background rate of facility births [36]. The four studies of multi-component interventions that reported on birth with a skilled attendant found increased birth attendance by skilled providers in Indonesia (44.2% control, 69.8% intervention, p≤0.00) [37] and Tanzania (34.1% baseline, 51.4% post-intervention, p<0.05) [33], and increased met need for emergency obstetric care in Bangladesh (16.0% baseline, 39.8% post-intervention, p<0.01) [32], but variable results by provider cadre in rural Nepal with an observed decrease in birth attendance by doctors and an observed increase in attendance at home births by skilled providers [36].

Four studies reported on care-seeking for postpartum care for women [27–29,34]. Of the three studies designed specifically to assess the effect of male involvement, uptake of routine postpartum care increased following facility-based couples’ education in urban Nepal (RR 1.25, 95% CI 1.01–1.54) [28], but no change in routine postpartum care was detected following workplace-based education for fathers in Turkey [29] and no change in care-seeking during the postpartum period for maternal complications or illness was found following community-based education for men and women in Pakistan [27]. A complex primary healthcare program in Pakistan that included a male involvement component observed an increase in routine postnatal care visits (27.2% baseline, 84.5% post-intervention, no significance reported) [34].

### Effect on home care practices

Eight studies reported on birth preparedness and complications readiness [28,29,31–33,35–37]. Two studies specifically designed to assess male involvement looked at this outcome; one found increased birth preparedness and complications readiness following workplace-based education for fathers in Turkey (OR 24.30, 95% CI 10.62–55.60) [29] but no difference was found in another study of facility-based education in Nepal [28]. Of the remaining six studies of multi-component interventions that included men alongside other community members, birth preparedness and complications readiness increased following family and community education programs in two studies in India [31,35], and in Indonesia following a social mobilisation campaign [37]. Increases were observed following family and community education in Pakistan [32] and Tanzania [33] but with no significance reported, and there were unclear effects following a social mobilisation campaign in Nepal [36].

Three studies reported on maternal nutrition intake. One study in Pakistan specifically designed to assess the effect of male involvement found no difference in maternal diet or uptake of iron-folic acid supplementation following community-based education [27]. Two complex community-based education interventions in India that included men as part of a broader community engagement strategy increased uptake of nutrition supplements [31,35] and increased consumption of some nutrient-rich foods during pregnancy [35]. No studies reported on maternal or newborn nutrition status.

Four studies reported mixed findings for newborn feeding practices. Of three studies specifically designed to test the effect of male involvement, workplace-based education for fathers in Turkey led to increased early initiation of breastfeeding (OR 2.38, 95% CI 1.24–4.61) and improved breastfeeding continuation [29], while facility-based education for couples in South Africa found no effect on breastfeeding initiation or continuation [26], and facility-based education for women and men in India increased early initiation of breastfeeding (47.3% control, 63.1% intervention, p<0.05) but reduced exclusive breastfeeding continuation to six months (62.0% control, 49.3% intervention, p<0.05) [30]. A complex community-based education intervention that engaged men alongside other community members in India found an increase in early breastfeeding initiation (1.7% baseline, 76.2% post-intervention, p<0.001) and did not report on breastfeeding continuation [31].

### Effect on mortality and morbidity

The two studies that reported on maternal deaths were both multi-component interventions that included a male involvement component [31,34]. One study documented a reduction in the maternal mortality ratio during a primary health care program in Pakistan [34], while no change in maternal mortality was found following family and community education in India [31].

Three studies reported on maternal complications or illness around the time of childbirth. Of the two studies designed to specifically test the effect of male involvement, one reported fewer complications during pregnancy or postpartum after facility-based education in India (37.7% control, 23.2% intervention, p<0.05) [30], but the other found no change during pregnancy, childbirth, or postpartum following community-based education for men and women in Pakistan [27]. A multi-component intervention that engaged men alongside other community members found fewer complications during childbirth after community education and training of health care providers in Eritrea (34% baseline, 13% post-intervention, p<0.001) [38]. No studies reported on maternal mental health outcomes.

Five studies reported on deaths in the perinatal period – three reported on stillbirths [26,30,31], one on perinatal mortality [27] and five on neonatal mortality [26,27,30,31,34]. Of the three studies specifically designed to test male involvement, there was an observed decrease in stillbirths (4% control, 2% intervention, no significance reported) and early neonatal mortality (2% control, 1% intervention, no significance reported) following facility-based education in South Africa [26], but no effect was found following a similar facility-based education intervention in India [30] or community-based education in Pakistan [27]. A community-based education program in India that targeted men alongside other community members found no change in stillbirth or neonatal mortality [31]. A primary health care program in Pakistan, which included outreach to male partners and community leaders as one component, documented a reduction in neonatal mortality but did not report statistical significance [34].

### Effect on couple relationships

Outcomes relating to couple relationships did not feature in the inclusion criteria but were extracted from included studies. These studies therefore comprise only a subset of the available evidence for the effect of male involvement interventions on couple relationships.

Three studies – all designed to specifically assess the effect of male involvement – reported on whether women and their male partners communicated or made decisions together about MNH [26,29,30]. Facility-based education for men and women increased couple communication about sexually transmitted infections in South Africa [26] and India [30], and increased communication about family planning use in India (63.9% control, 84.1% intervention, p<0.05) [30], but had no detectable effect in South Africa [26]. Joint decision-making about family planning use increased in India (76.8% control, 90.5% intervention, p<0.05) [30], but not following workplace-based education in Turkey [29]. Couples’ communication about the health of their baby increased following facility-based education in India (88.4% control, 94.5% intervention, p<0.05) [30], but not in South Africa, although that study found an increase in couple communication about child immunisation (75% control, 81% intervention, p<0.05) [26]. Increased communication regarding breastfeeding was reported following both facility-based education interventions [26,30], while workplace-based education in Turkey led to a large increase in men reporting joint decision-making about breastfeeding (OR 22.08, 95% CI 6.43–75.89) [29].

Four studies reported on practical and emotional support provided by men to their female partners or children. Of these, three were studies of facility- or workplace-based education programs specifically designed to assess the effect of male involvement [26,29,30]. Workplace-based education for expectant fathers in Turkey increased the proportion of men accompanying their partner for the majority of ANC visits (OR 3.00, 95% CI 1.32–6.79) and supporting good pregnancy nutrition (OR 9.00, 95% CI 1.98–40.81) [29]. Facility-based education for men and women in India did not affect the proportion of men accompanying their partners to the antenatal clinic but increased the proportion of men who remained in the room during antenatal counselling and education (15.6% control, 32.4% intervention, p<0.05) [30]. Facility-based couples education in South Africa found no change in men’s assistance or support during childbirth, with the exception of assisting in an emergency during labour (30% control, 43% intervention, p = 0.004) [26]. The similar facility-based education program in India increased the proportion of women reporting their male partner was nearby during labour and delivery (21.2% control, 30.7% intervention, p<0.05) and increased the proportion of men accompanying their partner during a postpartum check-up (47.2% control, 57.7% intervention, p<0.05), but found no change in the proportion of men taking their baby to a health facility for immunisation [30]. Workplace-based education in Turkey increased support provided by men for baby care, and increased men’s contribution to housework after their child was born [29]. There was an observed increase in men supporting their partner to breastfeed following facility-based education in South Africa [26], but no effect following the similar program in India [30]. The fourth study to report on support provided by men was a complex family and community education intervention in rural India, which included male involvement as one of several components [35]. This intervention had no effect on men’s engagement in ANC, but led to observed increases in the proportion of men supporting their partner by completing housework during pregnancy, providing emotional support and supporting their partners to access health services [35]. The same study reported qualitative findings that men’s perceptions of pregnancy had changed from being “their [women’s] issue” to concern and support [35].

Four studies reported on outcomes relating to women’s decision-making autonomy. Three of these were specifically designed to assess the effect of male involvement [26,29,30]. Facility-based education for women and men in India increased joint decision-making about whether to have sexual intercourse (intervention 92.4%, control 83.4%, p<0.05) but had no effect on joint decision-making about whether women could go to a health clinic if unwell [30]. The study of workplace-based education for expectant fathers in Turkey reported qualitative findings that some women were better able to act on their decisions about breastfeeding practices due to support received from male partners [29]:

[B]ecause this [exclusive breastfeeding] was emphasized so much in these programmes, he understood the importance and he was able to say…to me “No, don’t pay attention to them. We are doing the right thing.”

The same study, however, found some women experienced pressure from their male partners to adopt specific behaviours that had been recommended through the intervention, with the authors reporting some men used their knowledge to ‘dominate decision-making about pregnancy nutrition and infant care’ [29]. Similarly, facility-based education for couples in South Africa led to an observed increase in the proportion of women who had ceased breastfeeding because they were advised to do so by their mother or male partner (5% control, 13% intervention, no significance reported) [26]. The fourth study to report on women’s autonomy, a complex education intervention in India that engaged men as one component of a community engagement strategy, increased the proportion of women making decisions about birth planning, including where to give birth, transport for childbirth, and saving money to meet childbirth-related expenses (p≤0.001) [35].

## Discussion

### Contribution of this review

Recent systematic reviews focused on male accompaniment to antenatal care for perinatal health [16], male involvement for maternal health [14] or MNH [15], parenting interventions [17] and gender-integrated interventions [18] all conclude that male involvement is a promising intervention, but with limited evidence of effectiveness, particularly for mortality and morbidity outcomes. These studies support the findings of this review that male involvement interventions can have a positive effect on health service utilisation [14,15] and home care practices such as breastfeeding [15], while at the same time intervention effects can be variable and some negative outcomes have been identified [16]. One review [14] that included maternal mental health as a primary outcome found male involvement interventions were associated with reduced postpartum depression, whereas the inclusion criteria for this review were less sensitive to studies reporting on mental health (S3 File) and no included studies reported on maternal mental health outcomes. Additionally, recent systematic reviews focused on strategies to improve reproductive health outcomes indicate the value of engaging men to increase uptake of postpartum family planning [40] and health services during pregnancy and the postpartum period [41], echoing findings of this review.

The present review complements and adds to this body of work by including a broader range of studies specific to MNH, and capturing a comprehensive range of outcome measures that encompass key mechanisms by which male involvement can affect MNH (Fig 1). Review findings therefore provide guidance on not only whether, but also how, male involvement can affect maternal and newborn health outcomes. Findings are of direct and immediate relevance to policy-makers, practitioners and clinicians who seek to interpret and apply the current WHO health promotion guidelines that recommend male involvement, and complement a recent series of papers focused on implementation considerations for the WHO guidelines [42].

### Evidence for the effectiveness of male involvement

As described above, there are several interrelated mechanisms by which male involvement in MNH can support improved health outcomes: improved care-seeking behaviours and home care practices, underpinned by changed couple relationships, are expected to contribute to improved mortality and morbidity outcomes (Fig 1). The review identified positive, sometimes substantial, effects on health care-seeking associated with engaging men in MNH. However, these effects were generally observed following interventions with multiple integrated components. With the exception of an increase in postpartum visits in one study [28], the five studies specifically designed to test the effect of male involvement found no change in maternity care service utilisation. The review also found male involvement – either alone or alongside other intervention components – can affect care provided in the home to women and newborns, but found important variation in this effect with some harmful outcomes. For example, while several studies reported improved breastfeeding outcomes, one study found decreased exclusive breastfeeding [30] and another study reported more women ceasing breastfeeding due to advice from family members [26]. Effects of male involvement on mortality and morbidity are less clear, although there is some evidence of a positive effect on maternal morbidity. Most studies were not designed or powered to capture an effect on these outcomes. Finally, the review found that interventions in included studies affected couple relationships. These effects were variable and, importantly, detrimental effects were found on women’s autonomy in two studies [26,29], even where other measures of couple relationships had improved.

Even in the absence of direct evidence of an effect on mortality and morbidity, review findings that male involvement interventions can affect care-seeking, home care practices, and couple relationships demonstrate that male involvement has a plausible effect on mortality and morbidity and is therefore a viable health promotion strategy to improve MNH. The variation in effects identified in the review, including a small number of detrimental effects, underscore that intervention design and implementation are critical to ensure that the positive potential of male involvement is realised [43].

### Implications for future research and practice

While the review found that male involvement interventions can have many positive effects, findings also indicate that interventions must be carefully designed and implemented in order to support women’s autonomy and avoid reinforcing unequal gender relations. Interventions that engage men for MNH are typically implemented in settings where gender roles are strongly enforced and there are clear power differences between men and women. In such settings, men’s increased involvement in MNH as one of the ‘limited spaces in which women are empowered in patriarchal societies’ [44] could replicate existing gender inequalities in a new domain, to further disempower women [45]. The report of a woman describing that she felt infantilised by her husband using his new knowledge to tell her what to eat [29] echoes findings from other studies that when men become involved in MNH their involvement may weaken women’s autonomy in aspects of their lives where women have previously had a degree of authority [44].

The review also highlighted several methodological considerations of particular relevance to studies of male involvement. Recruitment of male partners for data collection was generally conducted via women, whether through communities [33,36,37] or health services [26,30], which weakened the statistical power of studies where fewer men were reached than anticipated. This has been identified as a persistent challenge in fatherhood research [43,46], and this review confirms the importance of considering alternative recruitment strategies, such as recruiting men directly, for example through workplaces [29] or other locations where men are present, or over-sampling women in order to achieve a minimum sample size of male partners. While only three studies [30,36,37] asked both men and women about the same outcomes, two of these reported important disagreements between women and men, for example regarding couple communication about MNH [30], men’s participation in maternity care services [30], and whether or not women had given birth in a facility [36]. This indicates that men and women may have important differences in perspective or reporting bias that should be considered in data collection and interpretation of findings. The review also highlighted challenges in interpreting observed changes in couple relationships. Included studies demonstrated that increases in male partner support, couple communication or joint decision-making do not necessarily reflect more egalitarian relationships. Including additional study measures specific to power dynamics within couple relationships [47,48] could support a nuanced interpretation of observed changes in couple communication, decision-making, and men’s practical and emotional support.

The review confirms findings from earlier reviews that there are important gaps in the evidence base for male involvement. Systematically collected and reported qualitative data is needed, particularly regarding women’s autonomy, gender roles and norms, and power dynamics within couple relationships and households. Future research could draw on lessons from the large body of literature on working with men and boys for gender equality and sexual and reproductive health to inform study design, intervention design and implementation, and the selection of study measures.

### Limitations

The review captured a diverse group of studies with varying outcome measures, meaning it was not feasible to conduct meta-analysis or check for publication bias. The quality of included studies was appraised against the EPHPP quality criteria as moderate or weak, largely due to study design: few studies were specifically designed or powered to test the effect of engaging men, particularly on mortality and morbidity outcomes. It is therefore possible that male involvement interventions may have had additional, undetectable effects; however the effects that were detected in studies indicate plausible mechanisms by which male involvement can affect MNH outcomes, including mortality and morbidity. Additionally, study measures used to capture male involvement as an exposure were poorly defined and varied between studies, which has been identified as a challenge to identifying eligible studies in systematic reviews of male involvement interventions [11,43]. Nevertheless, it is likely that all eligible studies are included in the review because of the search strategy that drew on a systematic mapping of maternal health intervention studies [20], with included articles cross-checked with content experts at the WHO Technical Consultation [49]. This search strategy, while a key strength of this review, is also a limitation because the systematic mapping was very resource-intensive [20] and would be challenging to replicate. The systematic mapping covers all maternal health intervention studies conducted in LMICs and published between 2000 and 2012 [21], and therefore the included studies fall within this time period. To manage this limitation, we cross-checked our findings against systematic reviews related to male involvement in MNH published after 2012. We found these supported the findings of this review, with an additional effect on postpartum depression identified in one review [14]. While more recent studies were ineligible for inclusion in this review, the approach taken to data extraction and interpretation for included studies provides insight into the mechanisms of effectiveness of male involvement interventions, with review findings providing evidence for a plausible effect of male involvement on mortality and morbidity outcomes, which remains a gap in the literature on male involvement [14–18].

## Conclusion

Male involvement interventions can improve care-seeking for essential MNH services, and home care practices for women and newborns. Engaging men in MNH also affects couple relationship dynamics. While changes in care-seeking, home care practices and couple relationships are plausible mechanisms to improve mortality and morbidity outcomes, available evidence does not directly demonstrate an effect on mortality or morbidity. Inclusive and nuanced approaches to the design and implementation of interventions, such as involving both women and men in intervention design, and including study measures that capture power dynamics within couple relationships, are required to ensure that male involvement interventions affirm women’s autonomy and support women’s capacity to care for themselves and their newborns. Further research is required to examine the effect of male involvement on mortality and morbidity outcomes, to identify good implementation practices, and to assess women’s and men’s experiences of male involvement and monitor harmful outcomes, particularly on women’s autonomy, using strong study designs.

## Supporting information

S1 Table Study and intervention characteristics. (PDF)

S2 Table Health service utilisation outcomes. (PDF)

S3 Table Home care practices outcomes. (PDF)

S4 Table Mortality and morbidity outcomes. (PDF)

S5 Table Couple relationship outcomes reported in included studies. (PDF)

S1 File Protocol for the first stage of the systematic review. (PDF)

S2 File Protocol for the second stage of the systematic review. (PDF)

S3 File Addendum to protocol for the second stage of the systematic review. (PDF)

S4 File PRISMA checklist. (PDF)
