# Supplementary material for: Involving men to improve maternal and newborn health: A systematic review of the effectiveness of interventions
Source: PLoS One. 2018 Jan 25;13(1):e0191620. doi: 10.1371/journal.pone.0191620 (PMC5784936; doi:10.1371/journal.pone.0191620)
Supplement: S1 Table — (PDF) [file pone.0191620.s001.pdf]

Study	Study design[38]	Description of intervention	Setting	Years	Study population	Number of participants	EPHPP quality assessment
Studies designed to assess the effect of a male involvement intervention							
Kunene 2005	Cluster RCT	Facility-based couples' education at antenatal clinics	South Africa, rural and urban	2000-2003	Women who were pregnant (10–30 weeks' gestation) and in a relationship, and their male partners	Women<br>Intervention n=729<br>Control n=694<br><br>Men<br>Intervention n=608<br>Control n=558	Moderate
Midhet 2010	Cluster RCT	Community-based education for men and women; training of traditional birth attendants; and community mobilisation to improve referral	Pakistan, rural	1998-2002	Ever-married women aged <50 years who had been pregnant in the previous 12 months	Intervention n=703<br>Comparison n=836<br>Control n=1022	Moderate
Mullany 2007	RCT	Facility-based couples' education at antenatal clinics at a tertiary hospital	Nepal, urban	2003-2004	Women who were pregnant (16–28 weeks' gestation), currently married, and whose husbands were present at the health facility	Intervention n=145<br>Comparison n=148<br>Control n=149	Moderate
Sahip 2007	Cohort analytic	Workplace-based education for expectant fathers	Turkey, urban	Not reported	Expectant fathers	Intervention n=80<br>Control n=80	Moderate
Varkey 2004	Non-equivalent control group	Facility-based education for men and women individually or as a couple at antenatal clinics	India, urban	2001-2002	Women who were pregnant (10–26 weeks' gestation) and in a relationship, and their male partners	Women<br>Intervention n=327<br>Control n=302<br><br>Men<br>Intervention n=327<br>Control n=302	Moderate
Studies designed to assess the effect of multiple intervention components, including a male involvement intervention							
Fullerton 2005	Repeat cross-sectional	Family and community education (home visits and group discussions)	India, rural	1998-2001	Women who had participated in intervention activities and given birth during the intervention (previous 30–42 months)	Baseline n=600<br>Post-intervention n=833	Weak
Hossain 2006	Quasi-experimental	Family and community education (home visits and group discussions) and improved community-facility	Bangladesh, rural	1999-2001	Not reported	Not reported	Weak

Study	Study design[38]	Description of intervention	Setting	Years	Study population	Number of participants	EPHPP quality assessment
		linkages					
Mushi 2010	Before-and-after	Family and community education (home visits and group discussions)	Tanzania, rural	2004-2006	Women who were pregnant, lactating, or already had a child and their male partners	Women<br>Baseline n=138<br>Post-intervention n=173<br><br>Men<br>Baseline n=100<br>Post-intervention n=69	Weak
Purdin 2009	Program evaluation using health information system data	Primary healthcare program that included outreach to male partners and community leaders	Pakistan, rural refugee camp	1996-2008	Woman who had been pregnant or given birth during the intervention	Not reported	Weak
Sinha 2008	Before-and-after	Family and community education (home visits and public meetings), and improved community-facility linkages	India, rural	2004-2006	Women who had given birth in the previous 12 months	Baseline n=319<br>Post-intervention n=501	Weak
Sood 2004, Indonesia	Before-and-after with control at endline	Social mobilisation campaign targeting husbands, midwives, and other community members	Indonesia, rural and urban	1999-2003	Women who had had a live birth in the previous 15 months, and their male partners	Women<br>Intervention n=1,024<br>Control n=758<br><br>Men<br>Intervention n=300<br>Control n=283	Weak
Sood 2004, Nepal	Before-and-after	Social mobilisation campaign targeting husbands and mothers-in-law	Nepal, rural	2000-2003	Women who were pregnant or had had a live birth in the previous 3 months, and their male partners	Women<br>Baseline n=390<br>Post-intervention n=424<br><br>Men<br>Baseline n=236<br>Post-intervention n=246	Weak
Turan 2011	Before-and-after	Community education and training of health care providers	Eritrea, rural	2005-2007	Women who had given birth in the previous 12 months	Baseline n=216<br>Post-intervention n=129	Weak
