# Supplementary material for: Involving men to improve maternal and newborn health: A systematic review of the effectiveness of interventions
Source: PLoS One. 2018 Jan 25;13(1):e0191620. doi: 10.1371/journal.pone.0191620 (PMC5784936; doi:10.1371/journal.pone.0191620)
Supplement: S2 Table — (PDF) [file pone.0191620.s002.pdf]

Study	Antenatal care attendance	Birth with a skilled attendant	Facility birth	Postnatal care for women
Studies designed to assess the effect of a male involvement intervention				
Kunene 2005	-	-	-	-
Midhet 2010	Significant increase in women receiving routine antenatal care compared with control in both the intervention arm (AOR 2.9, 95% CI 1.6–5.0) and the comparison arm (AOR 2.4, 95% CI 1.4–4.3), but no significant difference between intervention and comparison (p>0.05)	-	No significant difference in women delivering in the district hospital compared with control in either the intervention arm (AOR 1.3, 95% CI 0.6–2.5) or the comparison arm (AOR 1.3, 95% CI 0.7–2.5), and no observed difference between intervention and comparison (no significance reported). No significant difference in women delivering in any other health facility (control 0.9%, intervention 1.9%, comparison 1.6%, p>0.05).	No significant difference in women's care-seeking at the district hospital during the postpartum period (control 5.1%, intervention 8.9%, comparison 10.4%, p>0.05)
Mullany 2007	No significant difference in ≥4 ANC visits between intervention and comparison (RR 1.06, 95% CI 0.95–1.18)	No significant difference in attendance by skilled provider between intervention and comparison (RR 1.00, 95% CI 0.93–1.09)	No significant difference in women delivering in a health institution between intervention and comparison (RR 0.98, 95% CI 0.91–1.05)	Significant increase in postpartum visit within 2 weeks from birth between intervention and comparison (RR 1.25, 95% CI 1.01–1.54)
Sahip 2007	-	-	-	No significant difference in postpartum check-ups between intervention and control (OR 1.57, 95% CI 0.68–3.63)
Varkey 2004	-	-	-	-
Studies designed to assess the effect of multiple intervention components, including a male involvement intervention				
Fullerton 2005	-	-	-	-
Hossain 2006	-	Significant increase in met need for emergency obstetric care (baseline 16.0%, post-intervention 39.8%, p<0.01)	Significant increase in facility births (baseline 2.4%, post-intervention 20.5%, p<0.01)	-
Mushi 2010	No significant difference in ≥4 ANC visits among primigravida women (baseline 42.2%, post-intervention 51.3%, p>0.05)	Significant increase in birth with a skilled attendant (baseline 34.1%, post-intervention 51.4%, p<0.05)	Observed increase in institutional deliveries (baseline 33.3%, post-intervention 49.8%, no significance reported)	-
Purdin 2009	Observed increase in ≥3 ANC visits (baseline 49%, post-intervention 90%, no significance reported)	-	Observed increase in births in an emergency obstetric care facility (baseline 4.8%, post-intervention 67.2%, no significance reported)	Observed increase in postnatal care within 72 hours from birth (baseline 27.2%, post-intervention 84.5%, no significance reported)
Sinha 2008	Significant increase in ≥4 ANC visits (baseline 61%, post-intervention 72.5%, p<0.001)	-	Significant decrease in home births (baseline 54.1%, post-intervention 38.4%, p<0.001)	-
Sood 2004, Indonesia	Significant increase in ≥4 ANC visits (control 83.8%, intervention 94.4%, p≤0.00)	Significant increase in birth attendance by skilled provider (control 44.2%, intervention 69.8%, p≤0.00)	Significant decrease in home births (control 5.7%, intervention 11.4%, p≤0.00)	-
Sood 2004, Nepal	No observed difference in ≥4 ANC visits (control 75.1%, intervention 75.4%, no	Observed decrease in birth attendance by doctors (control 42.0%, intervention	Observed increase in home births (control 58.0%, intervention 70.7%, no	-

	significance reported)	29.3%, no significance reported). No observed difference in birth attendance by nurses (control 0.0%, intervention 0.0%). Observed increase in birth attendance at home by skilled provider (control 0.0%, intervention 4.0%, no significance reported).	significance reported). Observed decrease in births in hospital (control 42.0%, intervention 29.3%, no significance reported).	
Turan 2011	Significant increase in ≥4 ANC visits (OR 17.09, 95% CI 9.85–29.66)	-	Significant increase in births at a health facility (OR 26.24, 95% CI 11.42–60.27)	-
