# Supplementary material for: Involving men to improve maternal and newborn health: A systematic review of the effectiveness of interventions
Source: PLoS One. 2018 Jan 25;13(1):e0191620. doi: 10.1371/journal.pone.0191620 (PMC5784936; doi:10.1371/journal.pone.0191620)
Supplement: S3 Table — (PDF) [file pone.0191620.s003.pdf]

Study	Birth preparedness and complications readiness	Maternal nutrition	Newborn nutrition and breastfeeding
Studies designed to assess the effect of a male involvement intervention			
Kunene 2005	-	-	Observed increase in breastfeeding initiation both immediately (control 63%, intervention 66%, no significance reported) and within one hour from birth (control 16%, intervention 17%, no significance reported). Observed decrease in commencing mixed feeding before 2 months (control 34%, intervention 30%, no significance reported).
Midhet 2010	-	No significant difference in women taking iron-folic acid tablets compared with control in either the intervention arm (AOR 1.2, 95% CI 0.8–1.8) or the comparison arm (AOR 1.1, 95% CI 0.9–1.5), no significance reported for difference between intervention and comparison. Significant increase in women improving diet in pregnancy compared with control in the intervention arm (AOR 1.2, 95% CI 1.0–1.6) but no significant difference in the comparison arm (AOR 1.2, 95% CI 0.9–1.5), no significance reported for difference between intervention and comparison.	-
Mullany 2007	No significant difference in making ≥4 birth preparations (purchase safe delivery kit, save money for delivery, arrange blood donor, arrange transport for delivery, make emergency plan) between intervention and comparison (RR 1.30, 95% CI 0.78–2.15)	-	-
Sahip 2007	Significant increase in fathers making birth preparations (OR 24.30, 95% CI 10.62–55.60, p<0.01)	-	Significant increase in breastfeeding initiation within 1 hour from birth (OR 2.38, 95% CI 1.24–4.61, p<0.01). Significant increase in exclusive breastfeeding to 3 months (OR 3.44, 95% CI 1.74–6.82, p<0.01). Significant decrease in supplementary feeding before 6 months (OR 0.19, 0.09–0.37, p<0.01). Significant increase in breastfeeding continuation to 9 months (OR 2.64, 95% CI 1.36–5.09, p<0.01).
Varkey 2004	-	-	No difference in whether baby was ever breastfed (control 99.6%, intervention 100.0%, p>0.05). Significant increase in breastfeeding initiation within 1 hour from birth (control 47.3%, intervention 63.1%, p<0.05). Significant decrease in exclusive breastfeeding to 6 months (control 62.0%, intervention 49.3%, p<0.05).
Studies designed to assess the effect of multiple intervention components, including a male involvement intervention			
Fullerton 2005	Significant increase in women making ≥1 birth preparations (save money, arrange transport for delivery,	Significant increase in women taking iron-folic acid tablets or syrup (baseline 0.9%, post-intervention	Significant increase in breastfeeding initiation within 1 hour from birth (baseline 1.7%, post-intervention 76.2%,

	identify referral site) (baseline 0.15%, post-intervention 82.4%, p<0.001)	35.8%, p<0.001)	p<0.001)
Hossain 2006	Observed increase in women knowing ≥4 obstetric danger signs when prompted (intervention 45%, comparison 4%, no significance reported)	-	-
Mushi 2010	Observed increase in women and men able to identify 3 danger signs during pregnancy (baseline 52.9%, post-intervention 56.2%, no significance reported) and able to mention 3 complications during delivery (baseline 44.9%, post-intervention 52.9%, no significance reported). Observed increase in women and men able to identify ≥3 practices that contribute to delay in seeking care (baseline 46.6%, post-intervention 55.9%, no significance reported).	-	-
Purdin 2009	-	-	-
Sinha 2008	Significant increase in women saving money to meet delivery expenses (baseline 43.3%, post-intervention 67.7%, p≤0.001) and identifying or deciding transport for delivery (baseline 28.0%, post-intervention 52.1%, p≤0.001). Significant increase in women identifying a hospital/facility for delivery (baseline 40.2%, post-intervention 65.3%, p≤0.001) or in case of emergency (baseline 35.0%, post-intervention 49.4%, p≤0.001). Observed decrease in women identifying a birth attendant (baseline 44.5%, post-intervention 35.5%, no significance reported).	Significant increase in women consuming additional nutritious foods during pregnancy: green leafy vegetables (baseline 33.5%, post-intervention 65.5%, p≤0.001); milk (baseline 27.0%, post-intervention 33.9%, p≤0.001); lentils (baseline 20.1%, post-intervention 31.1%, p≤0.001); and eggs (baseline 15.4%, post-intervention 28.8%, p≤0.001). Significant increase in women receiving supplementary nutrition from the local health centre (baseline 45.1%, post-intervention 58.7%, p≤0.001).	-
Sood 2004, Indonesia	Significant increase in knowledge of bleeding as a danger sign during pregnancy among both women (control 16.4%, intervention 40.7%, p≤0.00) and male partners (control 11.0%, intervention 31.7%, p≤0.00). Significant increase in knowledge of danger signs during childbirth: severe bleeding among both women (control 12.3%, intervention 30.8%, p≤0.00) and male partners (control 8.8%, intervention 20.3%, p≤0.00); prolonged labour among women (control 4.2%, intervention 8.5%, p≤0.00) but no significant difference among male partners (control 4.2%, intervention 7.0%, p>0.05); and retained placenta among women (control 2.2%, intervention 4.1%, p≤0.05) but no significant difference among male partners (control 1.8%, intervention 3.7%, p>0.05). Significant increase in knowledge of severe bleeding as a danger sign during postpartum period among both women (control 10.3%, intervention 29.2%, p≤0.00) and male partners (control 9.2%, intervention	-	-

	19.7%, p≤0.00), but no significant difference in knowledge of high fever as a danger sign during the postpartum period among either women (control 0.3%, intervention 0.9%, p>0.05) or male partners (control 0.4%, intervention 1.3%, p>0.05).		
Sood 2004, Nepal	Observed increase in knowledge of vaginal bleeding as a danger sign during pregnancy among both women (baseline 30.5%, post-intervention 50.9%, no significance reported) and male partners (baseline 28.0%, post-intervention 31.7%, no significance reported). Observed increase in knowledge of severe vaginal bleeding as a danger sign during childbirth among both women (baseline 23.6%, post-intervention 47.6%, no significance reported) and male partners (baseline 17.4%, post-intervention 26.0%, no significance reported), but no observed difference in knowledge of retained placenta as a danger sign during childbirth among either women (baseline 5.4%, post-intervention 5.9%, no significance reported) or male partners (baseline 3.0%, post-intervention 3.3%, no significance reported) and an observed decrease in knowledge of prolonged labour as a danger sign during childbirth among both women (baseline 72.8%, post-intervention 55.9%, no significance reported) and male partners (baseline 69.1%, post-intervention 48.4%, no significance reported). Observed decrease in knowledge of severe bleeding as a danger sign during postpartum period among women (baseline 61.8%, post-intervention 59.2%, no significance reported) but an observed increase among male partners (baseline 48.7%, post-intervention 48.8%, no significance reported). Observed increase in knowledge of high fever as a danger sign during the postpartum period among women (baseline 23.3%, post-intervention 28.1%, no significance reported) but an observed decrease among male partners (baseline 27.1%, post-intervention 21.5%, no significance reported). Observed increase in knowledge of fast breathing as a danger sign in newborns (≤4 weeks after birth) among both women (baseline 45.4%, post-intervention 55.2%, no significance reported) and male partners (baseline 45.3%, post-intervention 48.8%, no significance reported)	-	-
Turan 2011	-	-	-
