# Supplementary material for: Involving men to improve maternal and newborn health: A systematic review of the effectiveness of interventions
Source: PLoS One. 2018 Jan 25;13(1):e0191620. doi: 10.1371/journal.pone.0191620 (PMC5784936; doi:10.1371/journal.pone.0191620)
Supplement: S4 Table — (PDF) [file pone.0191620.s004.pdf]

Study	Maternal mortality	Maternal morbidity and maternal mental health	Stillbirths	Perinatal mortality	Neonatal mortality
Studies designed to assess the effect of a male involvement intervention					
Kunene 2005	-	-	Observed decrease in stillbirths (control 4%, intervention 2%, no significance reported)	-	Observed decrease in death within 7 days from birth (control 2%, intervention 1%, no significance reported). No observed difference in death between 7 days and 6 months from birth (control 3%, intervention 3%, no significance reported).
Midhet 2010	-	No significant difference in illness during pregnancy compared with control in the intervention arm, but a significant decrease in the comparison arm (control 92.1%, intervention 90.7%, comparison 87.6%, p<0.05), no significance reported for difference between intervention and comparison. No significant difference in illness during delivery compared with control in the intervention arm, but a significant decrease in the comparison arm (control 60.8%, intervention 60.7%, comparison 52.6%, p<0.05), no significance reported for difference between intervention and comparison. No significant difference in illness immediately after delivery (control 45.1%, intervention 44.3%, comparison 40.2%, p>0.05). No significant difference in illness during the postpartum period compared with control in the intervention arm, but a significant decrease in the	-	No significant difference in perinatal mortality compared with control in the intervention arm (AOR 1.4, 95% CI 0.9–2.2) but a significant decrease in the comparison arm (AOR 0.5, 95% CI 0.3–0.7), no significance reported for difference between intervention and comparison	No significant difference in death within 7 days from birth compared with control in either the intervention arm (AOR 0.7, 95% CI 0.3–1.7) or the comparison arm (AOR 0.6, 95% CI 0.4–1.0), no significance reported for difference between intervention and comparison. No significant difference in death within one month from birth compared with control in either the intervention arm (AOR 0.9, 95% CI 0.5–1.6) or the comparison arm (AOR 0.7, 95% CI 0.4–1.0), no significance reported for difference between intervention and comparison.

		comparison arm (control 69.2%, intervention 62.8%, comparison 49.1%, p<0.05), no significance reported for difference between intervention and comparison.			
Mullany 2007	-	-	-	-	-
Sahip 2007	-	-	-	-	-
Varkey 2004	-	Significant decrease in complications in pregnancy or soon after delivery (control, 37.7% intervention, 23.2% p<0.05)	No observed difference in stillbirths (control 2.0% (n=6), intervention 1.5% (n=5), no significance reported)	-	No observed difference in death within 7 days from birth (control 0.0% (n=0), intervention 0.6% (n=2), no significance reported). No observed difference in death between 7 days and 6 months from birth (control 2.0% (n=6), intervention 1.8% (n=6), no significance reported)
Studies designed to assess the effect of multiple intervention components, including a male involvement intervention					
Fullerton 2005	No significant difference in maternal deaths (baseline 1.5%, post-intervention 0.4% p=0.053)	-	No significant difference in stillbirths (baseline 2.7%, post-intervention 4.4%, p=0.095)	-	No significant difference in neonatal mortality (baseline 4.2%, post-intervention 3.5%, p=0.559)
Hossain 2006	-	-	-	-	-
Mushi 2010	-	-	-	-	-
Purdin 2009	Observed decrease in maternal mortality ratio during the intervention (291/100,000 live births in 2000, 102/100,000 live births in 2004, no significance reported)	-	-	-	Observed decrease in neonatal mortality ratio during the intervention (25/1,000 live births in 2000, 20.7/1,000 live births in 2006, no significance reported)
Sinha 2008	-	-	-	-	-
Sood 2004, Indonesia	-	-	-	-	-
Sood 2004, Nepal	-	-	-	-	-
Turan 2011	-	Significant decrease in women or infants experiencing a problem during a recent birth (baseline 34%, post-intervention 13%, p<0.001)	-	-	-
