# Supplementary material for: Involving men to improve maternal and newborn health: A systematic review of the effectiveness of interventions
Source: PLoS One. 2018 Jan 25;13(1):e0191620. doi: 10.1371/journal.pone.0191620 (PMC5784936; doi:10.1371/journal.pone.0191620)
Supplement: S5 Table — (PDF) [file pone.0191620.s005.pdf]

Study	Couple communication and joint decision-making	Practical and emotional support from male partner	Maternal workload during pregnancy	Changes in women's decision-making autonomy
Studies designed to assess the effect of a male involvement intervention				
Kunene 2005	Significant increase in couple communication about STIs (control 64%, intervention 75%, p<0.05), sexual relations (control 75%, intervention 81%, p<0.05), immunisation (control 75%, intervention 81%, p<0.05) and breastfeeding (control 83%, intervention 87%, p<0.05). No significant difference in couple communication about family planning (control 70%, intervention 77%, p>0.05), whether to have more children (control 49%, intervention 54%, p>0.05), or the baby's health (control 71%, intervention 78%, p>0.05).	No significant difference in male partners assisting during labour (control 33%, intervention 38%, p>0.05), taking women to hospital during labour (control 61%, intervention 61%, p>0.05), arranging someone else to take women to hospital during labour (control 31%, intervention 31%, p>0.05), arranging transport during labour (control 13%, intervention 19%, p>0.05) or comforting women during labour (control 8%, intervention 8%, p>0.05). Significant increase in men assisting in an emergency situation during labour (control 30%, intervention 43%, p=0.004). Observed increase in male partners assisting in breastfeeding (control 72%, intervention 77%, no significance reported).	-	Observed increase in women stopping breastfeeding due to advice by mother or partner (control 5%, intervention 13%, no significance reported)
Midhet 2010	-	-	Significant increase in women reducing household work in pregnancy (control 17.5%, intervention 25.3%, comparison 18.5%, difference between the intervention arm and the comparison and control arms significant at p<0.01)	-
Mullany 2007	-	-	-	-
Sahip 2007	Significant increase in joint decision-making about infant feeding 3 months after birth (OR 22.08, 95% CI 6.43–75.89, p<0.01) and 9 months after birth (OR 26.33, 95% CI 3.44–201.76, p<0.01). No significant difference in joint decision-making about contraception 3 months after birth (OR 1.63, 95% CI 0.68–3.92) or 9 months after birth (OR 1.59, 95% CI 0.61–4.12).	Significant increase in male partners accompanying women on more than half the antenatal check-ups (OR 3.00, 95% CI 1.32–6.79, p<0.01). Significant increase in men supporting good pregnancy nutrition (OR 9.00, 95% CI 1.98–40.81, p<0.01). Significant increase in men changing nappies 3 months after birth (OR 5.98, 95% CI 2.92–12.26, p<0.01) and 9 months after birth (OR 4.53, 95% CI 2.26–9.06, p<0.01), and dressing the baby 9 months after birth (OR 11.67, 95% CI 5.23–26.03, p<0.01). No significant difference in men playing with the baby (OR 1.30, 95% CI 0.34–5.04). Significant increase in men	-	The authors report qualitative findings that some women experienced pressure from their male partners to adopt behaviours that had been recommended through the intervention, and to prioritise the baby above their own needs or preferences.

		contributing to housework 3 months after birth (control 7.60/40, intervention 10.90/40, p≤0.000) and 9 months after birth (control 7.62/40, intervention 9.61/40, p≤0.013). The authors report qualitative findings that several women reported that their husbands had joined them in resisting family pressure and adopting recommended health behaviours.		
Varkey 2004	Significant increase in couple communication about family planning use (control 63.9%, intervention 84.1%, p<0.05), the baby's health (control 88.4%, intervention 94.5%, p<0.05) and breastfeeding (control 74.8%, intervention 89.0%, p<0.05). No significant difference in couple communication about STIs and HIV/AIDS (control 9.9%, intervention 13.5%, p>0.05). Significant increase in joint decision-making about whether to use family planning (control 76.8%, intervention 90.5%, p<0.05), what family planning method to use (control 78.8%, intervention 90.2%, p<0.05) and whether to take a sick child to the clinic (control 81.1%, intervention 90.2%, p<0.05). No significant difference in joint decision-making about when to have another child (control 84.8%, intervention 90.5%, p>0.05).	No significant difference in male partners accompanying women to antenatal clinics (control 86.4%, intervention 90.5%, p>0.05) or being in the room during antenatal physical examinations (control 0.7%, intervention 2.1%, p>0.05). Significant increase in male partners being in the room during antenatal counselling (control 15.6%, intervention 32.4%, p<0.05), nearby during labour and delivery (control 21.2%, intervention 30.7%, p<0.05), in the room during family planning consultations (control 12.3%, intervention 27.8%, p<0.05) and accompanying women during the postpartum visit (control 47.2%, intervention 57.7%, p<0.05). No significant difference in male partners encouraging women to breastfeed (control 95.4%, intervention 89.1%, p>0.05). No significant difference in male partners taking the baby for immunisation either alone (control 0.4%, intervention 1.3%, p>0.05) or together with women (control 48.9%, intervention 57.4%, p>0.05).	-	Significant increase in joint decision-making about whether to have sexual relations (control 83.4%, intervention 92.4%, p<0.05), but no significant difference in joint decision-making about whether women can go to the clinic if unwell (control 66.9%, intervention 73.1%, p>0.05)
Studies designed to assess the effect of multiple intervention components, including a male involvement intervention				
Fullerton 2005	-	-	-	-
Hossain 2006	-	-	-	-
Mushi 2010	-	-	-	-
Purdin 2009	-	-	-	-
Sinha 2008	-	No significant difference in male partners accompanying women to antenatal	Observed increase in women reporting they reduced housework while pregnant	Significant increase in women deciding whether to deliver in their natal or marital

		clinics (baseline 50.9%, post-intervention 54.0%, p>0.05). Observed increase in male partners providing support for women to access health services (baseline 46.3%, post-intervention 57.7%, no significance reported), emotional support to women (baseline 39.6%, post-intervention 50.8%, no significance reported) and contributing to household work (baseline 27.4%, post-intervention 41.7%, no significance reported). The authors report qualitative findings that husbands' attitudes towards supporting pregnant women had changed from reluctance, and occasionally aggressive refusal, to acceptance and genuine concern.	(baseline 54%, post-intervention 76%, no significance reported). Observed increase in women reducing or stopping the following tasks while pregnant: carrying heavy weights (baseline 59.7%, post-intervention 71.7%, no significance reported), fetching water (baseline 40.6%, post-intervention 52.7%, no significance reported), washing clothes (baseline 18.9%, post-intervention 25.9%, no significance reported), agricultural work (baseline 42.8%, post-intervention 46.1%, no significance reported), non-household work (baseline 6.0%, post-intervention 30.7%, no significance reported).	home (baseline 73.2%, post-intervention 86.8%, $p \leq 0.001$) and deciding to deliver in an institution (baseline 67.1%, post-intervention 78.6%, $p \leq 0.001$)
Sood 2004, Indonesia	-	-	-	-
Sood 2004, Nepal	-	-	-	-
Turan 2011	-	-	-	-
