# Supplementary material for: Involving men to improve maternal and newborn health: A systematic review of the effectiveness of interventions
Source: PLoS One. 2018 Jan 25;13(1):e0191620. doi: 10.1371/journal.pone.0191620 (PMC5784936; doi:10.1371/journal.pone.0191620)
Supplement: S2 File — (PDF) [file pone.0191620.s007.pdf]

Guiding document for five systematic reviews for the WHO guidelines on health promotion interventions for maternal and newborn health

Contents

Acronyms....4

Introduction....4

Section 1: Stage 1 review....6

1.1 Objectives of Stage 1 of the review....6

1.2 Summary of methods for Stage 1 systematic review....6

1.3 Eligibility criteria for Stage 1....7

Section 2: Stage 2 review....11

2.1 Methods....11

2.1.1 Specify the P I C O for the systematic review....12

2.1.2 Define eligibility criteria for inclusion and exclusion of articles for each PICO....12

2.1.4 Define the variables for data extraction for each PICO....12

2.1.5 Compile the PICO specific Addendum....13

2.1.6 Obtain approval for each PICO specific Addendum....14

2.1.7 Identify eligible studies from the various sources....14

2.1.8 Obtain full text documents and upload these into EPPI reviewer 4....15

2.1.9 Determine eligibility of full-text records and extract predefined variables....15

2.1.10 Quality assessment of eligible studies....15

2.1.11Perform quality checks on data extraction....15

2.1.12 Present relevant information in tables to enable grading....15

2.1.13 Analysis and presentation of Evidence Profile tables....15

2.2 Limitations of the systematic review....16

2.3 Stage 2 roles and responsibilities of the review team....16

2.3.1 Timelines and milestones for the Stage 2 systematic reviews....16

Annexes....17

Annex 1: Search strategies in Stage 1....17

PubMED search strategy....17

CINAHL search strategy....19

Embase search strategy....21

PsycINFO search strategy....23

Web of Knowledge search strategy....23

Annex 2. List of low- and middle-income countries....28

Annex 3a: Variables extracted in Stage 1 screening of titles and abstracts, and review of full text....30

Annex 3b: Generic variables extracted in Stage 2 from original research for all five PICOs....36

Annex 3c: Variables extracted from existing systematic reviews in Stage 2 37
Annex 4: Quality Assessment tools 38
Quality assessment of systematic reviews 38
Quality assessment of quantitative studies 40
Quality assessment of qualitative studies 44
References 46

Acronyms

**HICs**	High-income countries
**HIV**	
**LMICs**	Low- and middle-income countries
**MASCOT**	**M**ultilateral **A**ssociation for **S**tudying health inequalities and enhancing North-South and South-South **CO**opera**T**ion
**MCH**	Maternal and child health
**MH**	Maternal health
**MNH**	Maternal and newborn health
**NWO**	Netherlands Organization for Scientific Research
**PICO**	Population, Intervention, Comparator, Outcome,
**PROGRESS-Plus**	Place of Residence, Race/Ethnicity, Occupation, Gender, Religion, Education, Socioeconomic Status, and Social Capital, and Plus represents additional categories such as Age, Disability, and Sexual Orientation
**STIs**	Sexually Transmitted Infections
**TI/AB**	Title abstract
**WHO**	The World Health Organization
**WHO/MCA**	The World Health Organization Department of Maternal, Newborn, Child and Adolescent Health
**WOTRO**	Science for Global Development', part of the Dutch Organization for Scientific Research (NWO)

Introduction

This guiding document outlines the methods to be used for five systematic reviews addressing PICO questions for the World Health Organization (WHO) guidelines on Health Promotion for Maternal and Newborn Health (MNH).

The specific research questions (PICO) of interest include:

1. What strategies employed with women, men, communities and community leaders to increase male involvement have been effective in increasing care-seeking behaviour during pregnancy, for child birth and after birth for the woman and newborn and in improving key maternal and newborn health outcomes?
2. What strategies for community transport schemes are effective in increasing care-seeking behaviour during pregnancy, for child birth and after birth for the woman and newborn and in improving key maternal and newborn health outcomes?
3. Does allowing a woman to have a companion of choice to accompany her during labour and birth in the facility/ or in the presence of a SA lead to an increase in births with a skilled attendant, institutional births and satisfaction with the birth experience, and in improving key maternal and newborn health outcomes?
4. What strategies to find new roles for TBAs within the formal health system are effective for increasing childbirth with a skilled attendant and for improving other key maternal and newborn health outcomes
5. What strategies for maternity waiting homes are effective in increasing birth with a skilled attendant/ institutional birth and improving other key maternal and newborn health outcomes?

The review consists of two phases. Firstly, Stage 1, which entailed developing a systematic map of maternal health literature (see Section 1 below). Thereafter, for Stage 2, this document outlines the methods for addressing the specific review questions to be addressed, using the literature identified in Stage 1 and complemented with other sources, as applicable.

This guiding document thus briefly sums Stage 1 of the review, and details the procedures for Stage 2 systematic reviews on the five WHO/MCA PICO questions above (see flowchart figure and Section 2). The structure of this guiding document is as follows:

Section 1 presents the aims and objectives of the Stage 1 review (Section 1.1); and the methods including the inclusion and exclusion criteria for Stage 1 (Section 1.2). Additional details are provided in the annexes with: the search strategies used to locate potentially eligible articles in Stage 1 of the review (Annex 1); the World Bank list of low- and middle-income countries for these reviews (Annex 2); and the variables extracted in Stage 1 of the review (Annex 3a). The full protocol for the Stage 1 review is available on request.

Section 2 provides an outline of the methods to be used for the Stage 2 reviews, building on the literature located in the stage 1 review (Section 2.1). This is then followed by a description of the methods to be used in the Stage 2 review (Section 2.2).

The stage 2 of the review begins with defining the specific review questions presented in the PICO format. This information and PICO specific methodologies will be presented in an Addendum separate for each PICO. Approval will be obtained in writing from WHO. EPPI-Reviewer 4 will be used for data extraction in Stage 2 of the review (http://eppi.ioe.ac.uk/eppireviewer4/). This software is developed and maintained by the EPPI-Centre of the Institute of Education at the University of London, United Kingdom. Data will be tabulated and prepared for grading before final presentation of the Evidence Profile tables.

Section 1: Stage 1 review

1.1 Objectives of Stage 1 of the review

The stage 1 review was conducted to satisfy the requirements for some components of two projects: the MASCOT project (which received funding from the European Union's Seventh Framework Programme FP7/2007-2013 under grant agreement number 282507); and the Wotro project, with funding from NWO. The Department of Maternal, Newborn, Child and Adolescent Health ofWHO (WHO/MCA) provided funding for reviewers to identify articles addressing the PICOs relevant for the WHO health promotion for MNH guidelines.

Overall, the review aimed to systematically map the evidence on interventions related to health systems and selected tracer conditions concerning maternal health. The review seeks to identify which conditions have been targeted by maternal health researchers in which LMICs, or regions of the world. This review used a health system framework, encompassing the WHO building blocks and patient demand, (described in the definitions and concepts section included in the protocol). The first stage aimed to provide a broad overview of all maternal health interventions published in the last decade.

This first review stage allows for identification of potentially relevant articles for inclusion in reviews of subsequent specific PICO questions. The review also enables us to describe the proportion of maternal health literature that focuses on interventions addressing HIV and other Sexually Transmitted Infections (STIs), hypertension, malaria, or haemorrhage, and the study designs used for maternal health systems research, as well as other key characteristics of maternal health research since 2000. The results will be collated and published in scientific publications.

The Stage 1 review objectives were:

1. To systematically map studies on health system, health promotion or key clinical interventions for maternal health;
2. To systematically identify evidence on the distribution of research on interventions to improve maternal heath in LMICs, identifying differentials in research outputs
3. To assess the extent to which maternal health interventions are explicitly designed and evaluated to address inequities in maternal health (whether effects on equity are explicitly taken into account when designing the intervention
4. Determine the influence of HICs on research in maternal health in LMICs
5. Determine which health system building blocks have been focused on in this research and in which countries or regions of the world
6. Build a team of collaborators working together across several continents.

1.2 Summary of methods for Stage 1 systematic mapping

Primary evidence published in peer-reviewed literature was systematically identified and data extracted into standardised data forms and then analysed. Data were extracted from studies of maternal health interventions related to the health system, health promotion, patient demand or selected tracer conditions (HIV, STIs, Malaria, hypertension and haemorrhage). Studies of individual clinical interventions (other than the selected tracer conditions), or descriptive studies, such as needs assessments were excluded. See Annex 1 for the search terms.

Reviewers initially screened titles and, if required, abstracts, with a low threshold for searching full text. Screening was done independently, in duplicate. Differences between reviewers in extractions were reconciled by a third reviewer. If no abstract was provided, but the title was indicative of a relevant study, these were coded as "No abstract" and then the full text assessed for eligibility.

The team then screened the full text of all articles included after the screening of title and abstract. These full text articles were checked to ensure that the codes applied during screening of the titles and abstracts were screened were correct.

Variables were extracted from the full text documents into four categories of codes (depending on the topic of the study), shown in the figure below. Annex 3a provides a detailed overview of all the variables extracted for each category of the code categories.

Table 1: Overview of the extracted code sets for the included studies with different intervention types.

**Intervention type**	**Code set:** Screening of full text for eligibility	**Code set:** Category A: Generic codes	**Code set:** Category C: Health System codes	**Code set:** Category D: Health promotion codes
**Clinical tracer conditions**	Done in full	Done in full	Only if applicable	Only if applicable
**Health system and community-based studies**	Done in full	Done in full	Done in full	Only if applicable
**Health promotion studies**	Done in full	Done in full	Only if applicable	Done in full

The articles identified in stage 1 as those eligible for Category D: Health promotion includes the studies relevant for the selected WHO PICOs to inform the WHO guidelines. Methodologies used for selection and extraction of the studies for each specific PICO is explained in Section 2 of this document.

1.3 Eligibility criteria for Stage 1

Original studies on maternal health (MH) interventions were included, as well as systematic reviews on MH. All study designs which provide evidence to answer the review question were included in Stage 1, provided that they report an outcome of an intervention. This broad inclusion was used as few randomized trials of health promotion or system interventions have been done in LMICs.

Much research in LMICs is not indexed by the major international research databases, thus we searched a range of research sources, including regional databases and registers of research specific to LMICs. Given the breadth of the potential research literature on maternal health, the search was both broad and inclusive.

Data sources include both unpublished and published literature, drawn from academic and other databases, as well as from experts (Annex 1). Piloting searches helped determine what research evidence to identify and assisted in refining the search criteria. A highly-sensitive search strategy, using both controlled vocabulary and free-text terms to identify studies on PubMED, was developed, and adapted for subsequent searches of other electronic sources. Search terms for maternal health

were combined with terms for LMICs, as defined by the World Bank (see Annex 2). Searches were limited to the period 2000 to 2012. but no language restrictions were employed.

The various databases with number of hits are shown below.

Box 2: The number of hits and date of search for each of the used databases.

Source	Number of hits	Date of search
**CINAHL**	2398	03/21/2012
**EMBASE**	3618	21/09/2012
**LILACS**	3450	21/09/2012
**Medline (PubMED)**	13,634	17/08/2012
**Popline**	12,186	21/09/2012
**PsycINFO**	1139	21/09/2012
**Web of Knowledge**	8903	21/09/2012
**Total**	**45,959**	

We exclude the effects of clinical interventions other than the tracer conditions (HIV and other STIs, hypertension, haemorrhage and malaria) we selected for review. For example, we excluded a study of the effects of iron supplementation for pregnant women. We, however, included individual health system interventions, such as an intervention to increase the numbers of midwives, to raise patient demand (such as counselling for improved care-seeking), or to remove user fees for childbirth services. Studies on the delivery of multiple interventions (complex interventions), such as a package of antenatal care, were included. We included studies that assessed different ways or modes of implementing single clinical interventions.

The box below presents the inclusion and exclusion criteria for Stage 1 (full definitions in the review protocol, available on request).

Box 3: Inclusion and exclusion criteria for Stage 1.

Inclusion criteria

All the criteria below must be met for inclusion in Stage 1:

1. **Population**. Interventions must target a maternal health population (*women in pregnancy, childbirth, or within two years postpartum*), or male involvement with a maternal health population, or be general health system interventions, provided they report outcomes in a maternal health population. If the intervention is among a maternal health population, but is primarily for the benefit of the child, it must still be included nonetheless.
2. **Study designs.** All study designs will be included provided they report on an assessment of the outcome of an intervention. Systematic reviews are also included, but narrative reviews are excluded and classified as “Not research”.
3. **Study outcomes.** To be included, quantitative or qualitative outcomes, or data on the impact of MH interventions at a population level must be reported. Outcomes may be measured in either the woman, or newborn child, male partners or health workers trained Biological, process, health systems and other outcomes measures are all applicable.
4. The following types of interventions:

4.1 **Health system or health promotion interventions** for improving maternal health. This includes studies of socio-economic or environmental interventions, such as improving water supply. Health system interventions generally fall within the 6 health system building blocks, listed in definitions section.

4.2 **Community-based interventions**. Interventions delivered in community settings (any activities occurring outside of health facilities), even delivery of single clinical interventions.

4.3 **Pre-specified single clinical interventions, as tracer conditions**, specifically: HIV/STIs; malaria, hypertension, haemorrhage and pregnancy sepsis.

5. **LMICs**. Only studies in LMICs will be included (Annex 4), LMIC countries defined by the World Bank in 2012 (*http://data.worldbank.org/about/country-classifications/country-and-lending-groups*).
6. **Dates of publication.** Studies published between 2000 and 2012 will be included.
7. **Languages.** Arabic, English, French, Japanese, Portuguese and Spanish language studies were included.

Exclusion criteria

1. **Study designs.** Exclude descriptive studies, such as those documenting prevalence of conditions and needs assessments. Policy discussion papers on system or multiple/complex interventions will be excluded unless they provide outcome data. Books are excluded
2. **Single-clinical interventions** Studies of the effectiveness of single clinical interventions were excluded (apart from the tracer conditions listed above).
3. **Topics.** We exclude interventions related to infertility or fertility (such as contraception failure rates). We only include interventions around contraception if part of a postnatal care intervention.
4. **Service utilisation articles**
5. **Academic theses**.

Flowchart 1: Overview of Stage 1 & Stage 2 of the systematic review

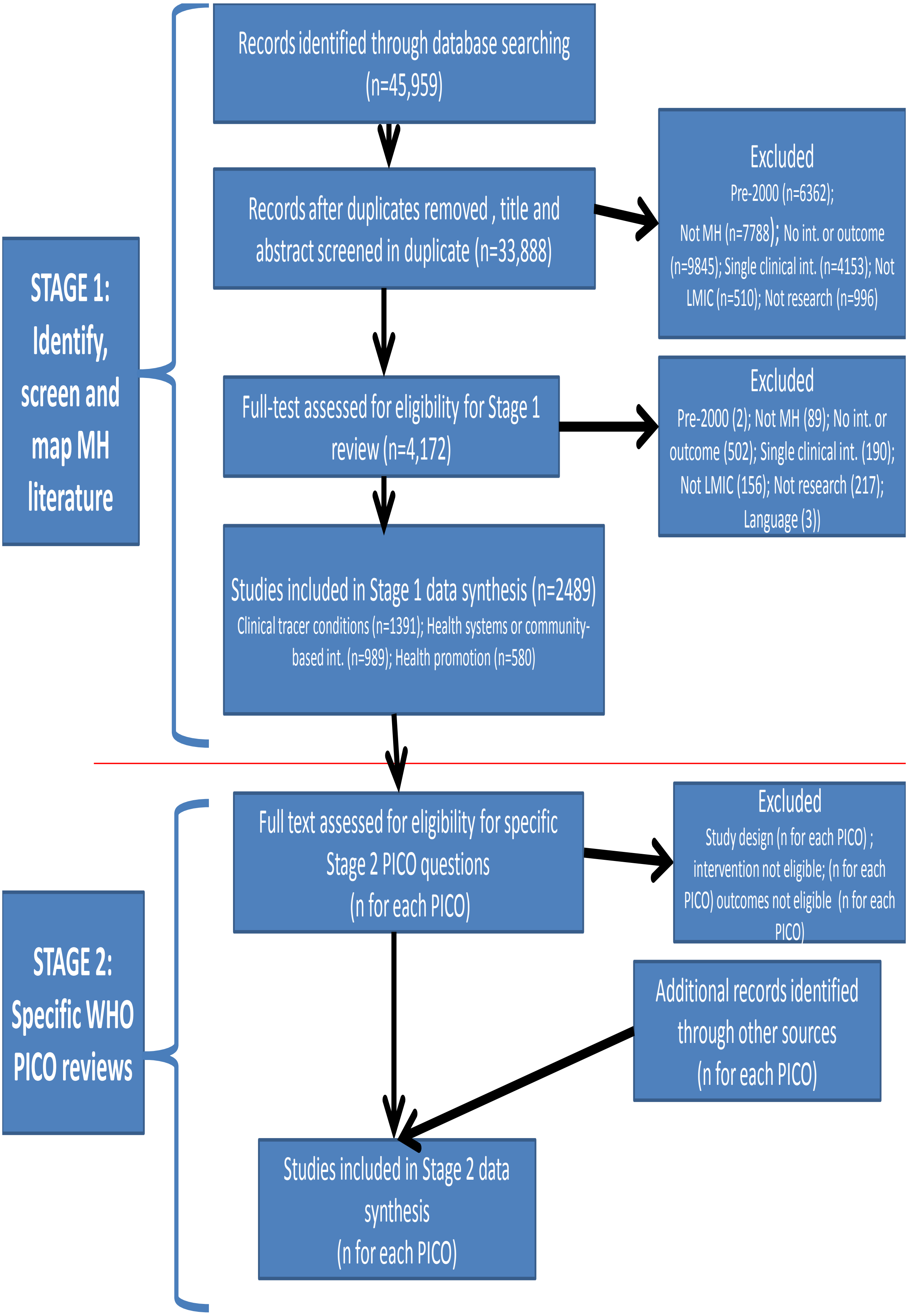

In total, 45,959 items were added to the online systematic review software EPPI-Reviewer 4. The software and individual reviewers then removed duplicate items totalling 12,071. Independently, in duplicate, we then screened the remaining records (33,888) for relevance on their title and abstract. This screening applied the review inclusion and exclusion criteria. The two reviewers or a third reviewer then reconciled any discrepancies in this coding.

From the 33,888 articles reviewed on title and abstract, 4472 were marked for full text review. This is an inclusion rate of 13.2% after screening of title and abstract. We were unable to locate the full text document for a total of 300 articles (6.7%; 300/4472). Of the 3140 full text articles reviewed, a further 45.3% were excluded (1889).

In total, 31.305 articles were excluded after screening of title and abstract and after full text review. This is 92.3% of all the articles identified in the review. Of the studies excluded from the review that were on maternal health, the most important reason for exclusion was that the study did not describe an intervention or outcome (33.0%; 10,347/31,305). Other studies that were on maternal health, but were excluded were those on single clinical interventions other than the tracer conditions (13.9%; 4343/31,305) or only provided data on utilisation of routine services (2.0%; 622/31,305). Other reasons for exclusion were: articles published before the year 2000 (20.3%; 6364); studies not on maternal health (25.2%; 7877/31,305); studies not done in LMICs (2.1%; 666/31,305); Not research (3.9%; 1213/31,305); and an excluded language (1.0%; 303/31,305).

Section 2: Stage 2 review

2.1 Methods

The first step in Stage 2 is the development of a generic guidance document for the Stage 2 review (this document). Stage 2 consists of the following steps outlined in Box 4 below. For each of the five reviews, specific methodological details are provided in a PICO-specific addendum, the structure of which matches the steps in the box below.

Box 4: Steps used for each of the selected PICO's

1. Specify the P I C O for the systematic review;
2. Identification and analysis of existing systematic reviews;
3. Define eligibility criteria for inclusion and exclusion of articles for each PICO question;
4. Define the variables for data extraction for each PICO review. The extracted variables consist of a generic code set and a PICO specific code set;
5. Compile the PICO specific Addendum. This encompasses the PICO definition; review methods; inclusion and exclusion criteria for the PICO; specific variables to extract for the review;
6. Obtain approval from WHO and representatives of the GDG for the generic guidance and for each Addendum;
7. Identify potentially eligible studies from the various sources, including the Stage 1 review and additional stage 2 strategies;
8. Obtain full text documents and upload these into EPPI reviewer 4;
9. Determine eligibility of full-text records and extract predefined data extraction variables;
10. Quality assessment of eligible systematic reviews (AMSTAR criteria), quantitative studies (EPHPP Quality assessment tool) and qualitative studies (Walsh 2006 appraisal tool; Annex 4)
11. Perform quality checks on data extraction;
12. Present relevant information in tables to enable grading and analysis of the evidence;
13. Analysis and presentation of Evidence Profile tables.

2.1.1 Specify the P I C O for the systematic review

An initial scoping exercise determined the PICOs which were later refined by the Guideline Development Group. For each of the PICOs, definitions will be detailed describing the Population, Intervention, Comparator and each of the outcomes of interest. In general, each of the reviews aims to assess the effectiveness of the health promotion intervention on care-seeking behaviour and MNH outcomes. More specifically detailed outcomes are described in the Addendums for each PICO.

2.1.2 Define eligibility criteria for inclusion and exclusion of articles for each PICO

PICO specific eligibility criteria are defined in the respective Addendum.

2.1.4 Define the variables for data extraction for each PICO

Generic variables to be extracted for all included articles of the five reviews are classified as:

- study inclusion and exclusion criteria;
- characteristics of the study population, controls (if any) and setting;
- characteristics of the intervention;
- outcome characteristics and actual outcomes;
- study design and quality;
- cost
- data regarding if the intervention is valued by the population and factors that influence effectiveness of these strategies.

More detailed information on the generic variables is provided in Annex 3b. The definition of each variable is provided in EPPI-Reviewer in the grey-shaded box at the bottom left of the screen for each variable.

Additional PICO specific variables could be extracted and will be defined in the respective Addendum. All generic and PICO specific codes will be piloted before finalisation.

The review team will only extract variables of studies reporting outcomes not assessed in existing reviews. Data that have already been extracted in the existing systematic reviews which are used as the main reference for the PICO (done in past two years and of high quality) will not be extracted – the data extraction tables from the original review will be used. This will be clarified in the respective Addendum.

Relevant variables already extracted in stage 1

Some variables already extracted during Stage 1 will be used in these Stage 2 reviews (i.e. reviewers do not have to extract these data).

1. Details of publication: author names, title, year of publication and journal name
2. Country(ies) where study done
3. Country(ies) of first author
4. Study population is a PROGRESS-Plus group? We marked "Yes", if study population was one of the PROGRESS-PLUS groups: Place of Residence, Race/Ethnicity, Occupation, Gender, Religion, Education, Socioeconomic Status, and Social Capital, and Plus represents additional categories such as Age, Disability and Sexual Orientation. We ticked "No" if the intervention was Universal**,** i.e. aimed at the whole group population, not on the basis of individual needs or risks).
5. Period mainly targeted by intervention (multiple-responses possible). This is the period(s) which the intervention mainly was delivered. Codes are:
    a. Pregnancy (this includes abortion and miscarriage)
    b. Childbirth
    c. Post birth (postpartum haemorrhage <6 hours after childbirth is not considered post-birth, but childbirth).
6. Funder name
7. Main implementing agency: National NGO; International NGO; Government; Research Group; Private sector; Other (add details). This is the group who does the work of implementing the intervention.
8. Intervention delivery extent Coded as: Entire country; More than one district but not entire country (Includes states); Single district; More than one facility but not entire district; Single facility (hospital or clinic); Other (add details) This is the extent to which an intervention is implemented, not the area evaluated, e.g. a programme implemented at national level but assessed in a few hospitals should be coded as "entire country".

2.1.5 Compile the PICO specific Addendum

All essential PICO specific information will be collated in an Addendum detailing the PICO definition; review methods; inclusion and exclusion criteria for the PICO; specific variables to extract for the review. This generic Guiding Document plus each of the PICO-specific Addendum provide the detailed methods and criteria of the review.

2.1.6 Obtain approval for each PICO specific Addendum

Written approvals will be sought from WHO and representative of the GDG for the generic Guiding document and each of the five PICO specific Addendums.

2.1.7 Identify eligible studies from the various sources

Stage 2 search builds on the Stage 1 search as described in section 1 (Annex 1) and additionally identifies relevant studies through review of reference lists and other records as well as through expert advice.

Reference lists of systematic reviews and other records

In stage 2 of the review, for the five PICO questions of this review, the bibliographies of included studies will be scrutinized to find additional studies. In particular, reference lists of systematic reviews related to these topics will be searched and potentially relevant articles reviewed. Studies included, and in some instances excluded, from systematic reviews related to the PICO questions will be assessed for inclusion in each systematic review. During the process of determining eligibility, relevant letters and commentaries on the health promotion topics were identified, the references of these documents will also be examined (articles coded as "Background" during Stage 1 coding).

Existing systematic review (or systematic reviews) can be used as the main reference for the PICO if they are of high quality, as assessed by the AMSTAR tool and done in the past two years. Additional data extraction will be done on studies reporting outcomes that were not included in these existing reviews. This data extraction will be done on the studies included in these existing reviews (even those published prior to 2000), supplemented by studies located in the MASCOT/Wotro review. The MASCOT/Wotro review only included articles in the period 2000-2012. For those existing systematic reviews that included references from pre 2000, these will remain included. For PICOs without existing reviews, the MASCOT database will be used as the source and will include articles published from the year 2000 on, unless there exists a justification to merit a search for articles published before the year 2000.

Expert opinion

For the Stage 2 reviews, experts in each of the topics have been contacted to request them to assist with identifying additional studies which may have relevant data for each PICO, particularly if these are unpublished studies.

All the documents identified through these methods described above, as well as any further studies identified by other means, will be assessed for eligibility in the review, according to the inclusion criteria for each PICO.

Other relevant documents

The review team will identify a few papers which contain useful references for the review, or which may be especially useful during writing up the background and findings of the review. These "background papers" did not necessarily meet all the inclusion criteria, but may be useful in analysis or contextualising the study findings. All background papers are also classified as exclude, include or query.

Duplicates

Duplicate articles may be found, the first of the duplicate articles will be coded using the code sets listed above, and then any subsequent duplicates coded as duplicate (articles with the same title, journal and date of publication).

2.1.8 Obtain full text documents and upload these into EPPI reviewer 4

All potentially eligible documents, both included and excluded documents, will be made available on EPPI reviewer 4 and made accessible in *.pdf.

2.1.9 Determine eligibility of full-text records and extract predefined variables

Eligibility of documents will be determined using the defined eligibility criteria. A single reviewer extracts the predefined data variables as detailed in the Generic codes (Annex 3b) AND the Addendum. Data from existing eligible systematic reviews will be extracted (Annex 3c) in an excel sheet.

2.1.10 Quality assessment of eligible studies

The quality of included studies will be appraised using validated tools specifically developed for appraisal of systematic reviews, quantitative studies and qualitative studies (Annex 4).
For systematic reviews the AMSTAR criteria will be applied (2-4). For both randomised and observational quantitative studies, quality will be assessed using the EPHPP tools (http://www.ephpp.ca/tools.html). Quality of qualitative studies will also be appraised using a standard tool (5). We aim for transparent reporting of judgements made. The quality assessment tools allows for risk of bias to be assessed for each included primary study.

2.1.11Perform quality checks on data extraction

Quality checks will be performed on extracted data with double extraction of at least 10% of the studies as specified in the Addendum as recent reviews exist for most PICOs. In selected circumstances it could be decided to increase the proportion of double data-entry where less experienced members are extracting data.

2.1.12 Present relevant information in tables to enable grading

The flowchart (Figure 1) depicts the steps taken in Stage 1 of the review and Stage 2 from identifying articles until synthesis of data. An individual flowchart will be created for each PICO to demonstrate the flow of articles for each PICO, both excluded and included items. The articles extracted will be summed to report i) the reasons for study exclusion; ii) the proportion of studies with the outcomes of interest; iii) the outcomes reported in included studies.

Results will be tabulated into three tables, specifically: Table 1 Characteristics of population and intervention; Table 2 Outcomes of study; Table 3 Quality of included studies.

Further analytical steps will occur thereafter, including the development of GRADE tables. The processes for GRADE table development are not covered in this guiding document.

2.1.13 Analysis and presentation of Evidence Profile tables

Evidence analysis and synthesis will be done separately for each of the five PICOs. For each PICO, we will interpret the cumulative evidence to draw conclusions about the relevance of results to the review question. The marked heterogeneity of study exposures and of outcomes means results will

almost certainly be summed descriptively or qualitatively, rather than in meta-analysis or by using summary measures. Data will thus be summed qualitatively, and presented in tables.

The synthesis of findings will include discussion of the applicability, transferability, and external validity of findings according to accepted criteria (directness of studies), as well as consider context of the studies. Attention to understanding context and process evaluation will aid judgments about applicability. The inclusion/exclusion criteria of the primary studies will influence the generalizability of findings. A judgement is required during the GRADE process about how much socio-cultural or political context shaped the original studies' interpretation, and if interventions that are effective in this setting will work in different contexts. Applicability relates to the context in which the primary data were collected and the setting to which they will be extrapolated.

2.2 Limitations of the systematic review

Publication bias is likely as negative or null findings of sub-group analyses are less likely to be reported than positive findings(6). We will do formal tests for publication bias, including using funnel plots. Many of the interventions reviewed are part of long-standing programmes and have not been evaluated or evaluations done are not included in the sources we searched. Publication bias may have occurred, with evaluations that have positive findings being more likely to be published than those with negative findings.

2.3 Stage 2 roles and responsibilities of the review team

2.3.1 Timelines and milestones for the Stage 2 systematic reviews

**Phase**	**1. Define in-depth review topics.**	**2. Finalise Stage 2 review protocol**	**3. Identify eligible literature**
**Duration**	**Week 1-2**	**Week 3-4**	**Week 5-6**
**Outputs**	Define final PICO questions. Draft codes for data extraction. Adapt CF for each individual review. Pilot codes, and finalise codes and protocol. Locate potentially relevant studies. Upload any additional PDFs into EPPI website.	Extract data in duplicate using EPPI centre tools. Quality control of data extraction.	Analyse data and prepare results tables.

Dates not provided for Stage 2 as the process is repeated for several reviews, beginning at different time points.

Annexes

Annex 1: Search strategies in Stage 1

PubMED search strategy

(((((non-pregnancy[All Fields] AND related[All Fields] AND ("infection"[MeSH Terms] OR "infection"[All Fields] OR "communicable diseases"[MeSH Terms] OR ("communicable"[All Fields] AND "diseases"[All Fields]) OR "communicable diseases"[All Fields])) OR non-pregnancy related[Title]) OR ((maternal[Title] OR pregnant[Title] OR pregnancy[Title] OR obstetric[Title] OR puerperal[Title] OR mother[Title] OR childbirth[Title] OR labour[Title] OR labor[Title] OR natal[Title] OR post-natal[Title] OR pre-natal[Title] OR prenatal[Title] OR antenatal[Title] OR ante-natal[Title] OR perinatal[Title] OR peri-natal[Title] OR puerperal[Title] OR puerperium[Title]) AND ((((((sepsis[Title] OR septic$[Title]) OR infection$[Title]) OR HIV[Title]) OR tuberculosis[Title]) OR pneumonia[Title]) OR meningitis[Title]))) OR (chorioamnionitis[Title/Abstract] OR "chorioamnionitis"[MeSH Terms])) OR ((("sepsis"[MeSH Terms] OR "sepsis"[All Fields]) OR septic$[All Fields] OR infection$[Title]) AND ((amniotic[Title/Abstract] OR intra-amniotic[Title/Abstract]) OR intraamniotic[Title/Abstract])))) OR ((((anemic[Title] OR anaemia[Title]) OR anaemic[Title]) OR anemia[Title]) AND (puerperal[Title] OR (((((maternal[Title] OR pregnant[Title]) OR pregnancy[Title]) OR obstetric[Title]) OR mother[Title]) OR childbirth[Title])))) OR (((((("Midwifery"[Mesh] OR dula[Title/Abstract]) OR ((("parturition"[MeSH Terms] OR "parturition"[All Fields] OR "birth"[All Fields]) AND attendant[All Fields]) OR (("parturition"[MeSH Terms] OR "parturition"[All Fields] OR "birth"[All Fields]) AND attendants[All Fields]))) OR ("residence characteristics"[MeSH Terms] OR ("residence"[All Fields] AND "characteristics"[All Fields]) OR "residence characteristics"[All Fields] OR ("place"[All Fields] AND "birth"[All Fields]) OR "place of birth"[All Fields])) OR (("Birthing Centers"[MAJR] OR "Delivery Rooms"[MAJR]) OR "Delivery, Obstetric/nursing"[MAJR])) OR ((maternal[Title] OR pregnant[Title] OR pregnancy[Title] OR obstetric[Title] OR puerperal[Title] OR mother[Title] OR childbirth[Title] OR labour[Title] OR labor[Title] OR natal[Title] OR post-natal[Title] OR pre-natal[Title] OR prenatal[Title] OR antenatal[Title] OR ante-natal[Title] OR perinatal[Title] OR peri-natal[Title] OR puerperal[Title] OR puerperium[Title]) AND (("Ambulances"[Mesh] OR "Health Services Accessibility"[Mesh]) OR "Transportation of Patients"[Mesh]))) OR (("Travel"[MeSH Terms] OR "Delivery of Health Care/organization and administration"[MAJR]) AND (maternal[Title] OR pregnant[Title] OR pregnancy[Title] OR obstetric[Title] OR puerperal[Title] OR mother[Title] OR childbirth[Title] OR labour[Title] OR labor[Title] OR natal[Title] OR post-natal[Title] OR pre-natal[Title] OR prenatal[Title] OR antenatal[Title] OR ante-natal[Title] OR perinatal[Title] OR peri-natal[Title] OR puerperal[Title] OR puerperium[Title])))) OR (ectopic pregnancy[Title/Abstract] OR "pregnancy, ectopic"[MeSH Terms])) OR (((((("Postpartum Hemorrhage"[Mesh] OR ((((((((((((((((maternal[Title] OR pregnant[Title]) OR pregnancy[Title]) OR obstetric[Title]) OR puerperal[Title]) OR mother[Title]) OR childbirth[Title]) OR labour[Title]) OR labor[Title]) OR natal[Title]) OR post-natal[Title]) OR pre-natal[Title]) OR prenatal[Title]) OR antenatal[Title]) OR ante-natal[Title]) OR perinatal[Title]) OR peri-natal[Title]) AND (Hemorrhage[Title] OR Haemorrhage[Title]))) OR (((obstetric[All Fields] AND ("haemorrhage"[All Fields] OR "hemorrhage"[MeSH Terms] OR "hemorrhage"[All Fields])) OR obstetric hemorrhage[Title/Abstract]) OR ("postpartum hemorrhage"[MeSH Terms] OR ("postpartum"[All Fields] AND "hemorrhage"[All Fields]) OR "postpartum hemorrhage"[All Fields] OR ("post"[All Fields] AND "partum"[All Fields] AND "hemorrhage"[All Fields]) OR "post partum hemorrhage"[All Fields]) OR ("postpartum hemorrhage"[MeSH Terms] OR ("postpartum"[All Fields] AND "hemorrhage"[All Fields]) OR "postpartum hemorrhage"[All Fields] OR ("post"[All Fields] AND "partum"[All Fields] AND "haemorrhage"[All Fields]) OR "post partum haemorrhage"[All Fields]) OR ("postpartum hemorrhage"[MeSH Terms] OR ("postpartum"[All Fields] AND "hemorrhage"[All Fields]) OR "postpartum hemorrhage"[All Fields] OR ("post"[All Fields] AND "partum"[All Fields] AND "hemorrhage"[All Fields]) OR "post partum hemorrhage"[All Fields]) OR ("postpartum hemorrhage"[MeSH Terms] OR ("postpartum"[All Fields] AND "hemorrhage"[All Fields]) OR "postpartum hemorrhage"[All Fields] OR ("post"[All Fields] AND "partum"[All Fields] AND "haemorrhage"[All Fields]) OR "post partum haemorrhage"[All Fields]))) OR obstetric hemorrhage[Title/Abstract]) OR "Hypertension, Pregnancy-Induced"[Mesh]) OR ((((obstructed labor[Title/Abstract] OR obstructed labour[Title/Abstract]) OR (obstetric fistula[Title/Abstract] OR obstetric fistulae[Title/Abstract])) OR ("vaginal fistula"[MeSH Terms] OR "vesicovaginal fistula"[MeSH Terms])) OR ("Obstetric Labor Complications"[Mesh] OR "Obstetric Labor, Premature"[Mesh]))) OR (((((((((((((((((maternal[Title] OR pregnant[Title]) OR pregnancy[Title]) OR obstetric[Title]) OR puerperal[Title]) OR mother[Title]) OR childbirth[Title]) OR labour[Title]) OR labor[Title]) OR natal[Title]) OR post-natal[Title]) OR pre-natal[Title]) OR prenatal[Title]) OR antenatal[Title]) OR ante-natal[Title]) OR perinatal[Title]) OR peri-natal[Title]) AND (hypertension[Title] OR blood pressure[Title])) AND (((((eclampsia[Title/Abstract] OR preeclampsia[Title/Abstract]) OR HELLP[Title/Abstract]) OR "eclampsia"[MeSH Terms]) OR "pre-eclampsia"[MeSH Terms]) OR pre-eclampsia[Title/Abstract])))) OR ("Pregnancy Complications, Hematologic"[Mesh] OR "Pregnancy in Adolescence"[Mesh] OR "Pregnancy Complications, Infectious"[Mesh] OR "Pregnancy Complications, Cardiovascular"[Mesh] OR "Pregnancy Complications"[Mesh] OR "Pregnancy, Prolonged"[Mesh])) AND (((("africa"[MeSH Terms] OR "africa"[All Fields]) OR (((((((("afghanistan"[MeSH Terms] OR "afghanistan"[All Fields]) OR ("bangladesh"[MeSH Terms] OR "bangladesh"[All Fields])) OR ("benin"[MeSH Terms] OR "benin"[All Fields])) OR ("burkina faso"[MeSH Terms] OR ("burkina"[All Fields] AND "faso"[All Fields]) OR "burkina faso"[All Fields])) OR (((((((((((((((((((((((((((("burundi"[MeSH Terms] OR "burundi"[All Fields]) OR ("cambodia"[MeSH Terms] OR "cambodia"[All Fields])) OR ("central african republic"[MeSH Terms] OR ("central"[All Fields] AND "african"[All Fields] AND "republic"[All Fields]) OR "central african republic"[All Fields])) OR ("chad"[MeSH Terms] OR "chad"[All Fields])) OR ("comoros"[MeSH Terms] OR "comoros"[All Fields])) OR (("congo"[MeSH Terms] OR "congo"[All Fields]) AND Dem.[All Fields] AND Rep[All Fields])) OR ("congo"[MeSH Terms] OR "congo"[All Fields])) OR DRC[Affiliation]) OR ("eritrea"[MeSH Terms] OR "eritrea"[All Fields])) OR ("ethiopia"[MeSH Terms] OR "ethiopia"[All Fields])) OR ("gambia"[MeSH Terms] OR "gambia"[All Fields])) OR ("guinea"[MeSH Terms] OR "guinea"[All Fields])) OR (("guinea"[MeSH Terms] OR "guinea"[All Fields]) AND Bisau[All Fields])) OR ("haiti"[MeSH Terms] OR "haiti"[All Fields])) OR ("kenya"[MeSH Terms] OR "kenya"[All Fields])) OR ("korea"[MeSH Terms] OR "korea"[All Fields])) OR Kyrgyz[All Fields]) OR ("liberia"[MeSH Terms] OR "liberia"[All Fields])) OR ("madagascar"[MeSH Terms] OR "madagascar"[All Fields])) OR ("malawi"[MeSH Terms] OR "malawi"[All Fields])) OR ("mali"[MeSH Terms] OR "mali"[All Fields])) OR ("mozambique"[MeSH Terms] OR "mozambique"[All Fields])) OR ("myanmar"[MeSH Terms] OR "myanmar"[All Fields])) OR ("nepal"[MeSH Terms] OR "nepal"[All Fields])) OR ("niger"[MeSH Terms] OR "niger"[All Fields])) OR ("rwanda"[MeSH Terms] OR "rwanda"[All Fields])) OR ("sierra leone"[MeSH Terms] OR ("sierra"[All Fields] AND "leone"[All Fields]) OR "sierra leone"[All Fields])) OR ("somalia"[MeSH Terms] OR "somalia"[All Fields]))) OR ((((("tajikistan"[MeSH Terms] OR "tajikistan"[All Fields]) OR ("tanzania"[MeSH Terms] OR "tanzania"[All Fields])) OR ("togo"[MeSH Terms] OR "togo"[All Fields])) OR ("uganda"[MeSH Terms] OR "uganda"[All Fields])) OR ("zimbabwe"[MeSH Terms] OR "zimbabwe"[All Fields]))) OR ("africa, northern"[MeSH Terms] OR ("africa"[All Fields] AND "northern"[All Fields]) OR "northern africa"[All Fields] OR "sahara"[All Fields])) OR sub-saharan[All Fields])) OR ((("angola"[MeSH Terms] OR "angola"[All Fields]) OR ("armenia"[MeSH Terms] OR "armenia"[All Fields])) OR ("belize"[MeSH Terms] OR "belize"[All Fields])) OR ("bhutan"[MeSH Terms] OR "bhutan"[All Fields])) OR ("bolivia"[MeSH Terms] OR "bolivia"[All Fields])) OR ("cameroon"[MeSH Terms] OR "cameroon"[All Fields])) OR ("cape verde"[MeSH Terms] OR ("cape"[All Fields] AND "verde"[All Fields]) OR "cape verde"[All Fields])) OR ("congo"[MeSH Terms] OR "congo"[All Fields])) OR ("cote d'ivoire"[MeSH Terms] OR ("cote"[All Fields] AND "d'ivoire"[All Fields]) OR "cote d'ivoire"[All Fields])) OR ("cote d'ivoire"[MeSH Terms] OR ("cote"[All Fields] AND "d'ivoire"[All Fields]) OR "cote d'ivoire"[All Fields] OR ("ivory"[All Fields] AND "coast"[All Fields]) OR "ivory coast"[All Fields])) OR ("djibouti"[MeSH Terms] OR "djibouti"[All Fields])) OR ("egypt"[MeSH Terms] OR "egypt"[All Fields])) OR ("el salvador"[MeSH Terms] OR ("el"[All Fields] AND "salvador"[All Fields]) OR "el salvador"[All Fields])) OR ("fiji"[MeSH Terms] OR "fiji"[All Fields])) OR

("georgia"[MeSH Terms] OR "georgia"[All Fields] OR "georgia (republic)"[MeSH Terms] OR ("georgia"[All Fields] AND "(republic)"[All Fields]) OR "georgia (republic)"[All Fields])) OR ("ghana"[MeSH Terms] OR "ghana"[All Fields])) OR ("guatemala"[MeSH Terms] OR "guatemala"[All Fields])) OR ("guyana"[MeSH Terms] OR "guyana"[All Fields])) OR ("honduras"[MeSH Terms] OR "honduras"[All Fields])) OR ("indonesia"[MeSH Terms] OR "indonesia"[All Fields])) OR ("india"[MeSH Terms] OR "india"[All Fields])) OR ("iraq"[MeSH Terms] OR "iraq"[All Fields])) OR ("micronesia"[MeSH Terms] OR "micronesia"[All Fields] OR "kiribati"[All Fields])) OR ("yugoslavia"[MeSH Terms] OR "yugoslavia"[All Fields] OR "kosovo"[All Fields])) OR Lao[All Fields]) OR ("lesotho"[MeSH Terms] OR "lesotho"[All Fields])) OR ("micronesia"[MeSH Terms] OR "micronesia"[All Fields] OR ("marshall"[All Fields] AND "islands"[All Fields]) OR "marshall islands"[All Fields])) OR ("mauritania"[MeSH Terms] OR "mauritania"[All Fields])) OR ("micronesia"[MeSH Terms] OR "micronesia"[All Fields])) OR ("moldova"[MeSH Terms] OR "moldova"[All Fields])) OR ("mongolia"[MeSH Terms] OR "mongolia"[All Fields])) OR ("morocco"[MeSH Terms] OR "morocco"[All Fields])) OR ("nicaragua"[MeSH Terms] OR "nicaragua"[All Fields])) OR ("nigeria"[MeSH Terms] OR "nigeria"[All Fields])) OR ("pakistan"[MeSH Terms] OR "pakistan"[All Fields])) OR ("papua new guinea"[MeSH Terms] OR ("papua"[All Fields] AND "new"[All Fields] AND "guinea"[All Fields]) OR "papua new guinea"[All Fields])) OR ("paraguay"[MeSH Terms] OR "paraguay"[All Fields])) OR ("philippines"[MeSH Terms] OR "philippines"[All Fields])) OR ("samoa"[MeSH Terms] OR "samoa"[All Fields])) OR ("atlantic islands"[MeSH Terms] OR ("atlantic"[All Fields] AND "islands"[All Fields]) OR "atlantic islands"[All Fields] OR ("sao"[All Fields] AND "tome"[All Fields] AND "principe"[All Fields]) OR "sao tome and principe"[All Fields])) OR (Sao[All Fields] AND Tome[All Fields])) OR ("senegal"[MeSH Terms] OR "senegal"[All Fields])) OR ("melanesia"[MeSH Terms] OR "melanesia"[All Fields] OR ("solomon"[All Fields] AND "islands"[All Fields]) OR "solomon islands"[All Fields])) OR ("sri lanka"[MeSH Terms] OR ("sri"[All Fields] AND "lanka"[All Fields]) OR "sri lanka"[All Fields])) OR ("sudan"[MeSH Terms] OR "sudan"[All Fields])) OR ("swaziland"[MeSH Terms] OR "swaziland"[All Fields])) OR ("syria"[MeSH Terms] OR "syria"[All Fields] OR ("syrian"[All Fields] AND "arab"[All Fields] AND "republic"[All Fields]) OR "syrian arab republic"[All Fields])) OR ("syria"[MeSH Terms] OR "syria"[All Fields])) OR ("east timor"[MeSH Terms] OR ("east"[All Fields] AND "timor"[All Fields]) OR "east timor"[All Fields] OR ("timor"[All Fields] AND "leste"[All Fields]) OR "timor leste"[All Fields])) OR ("indonesia"[MeSH Terms] OR "indonesia"[All Fields] OR "timor"[All Fields])) OR ("tonga"[MeSH Terms] OR "tonga"[All Fields])) OR ("turkmenistan"[MeSH Terms] OR "turkmenistan"[All Fields])) OR ("micronesia"[MeSH Terms] OR "micronesia"[All Fields] OR "tuvalu"[All Fields])) OR ("ukraine"[MeSH Terms] OR "ukraine"[All Fields])) OR ("uzbekistan"[MeSH Terms] OR "uzbekistan"[All Fields])) OR ("vanuatu"[MeSH Terms] OR "vanuatu"[All Fields])) OR ("vietnam"[MeSH Terms] OR "vietnam"[All Fields])) OR (("middle east"[MeSH Terms] OR ("middle"[All Fields] AND "east"[All Fields]) OR "middle east"[All Fields] OR ("west"[All Fields] AND "bank"[All Fields]) OR "west bank"[All Fields]) AND Gaza[All Fields])) OR Gaza[All Fields]) OR ("yemen"[MeSH Terms] OR "yemen"[All Fields])) OR ("zambia"[MeSH Terms] OR "zambia"[All Fields]))) OR ((("albania"[MeSH Terms] OR "albania"[All Fields]) OR ("algeria"[MeSH Terms] OR "algeria"[All Fields])) OR ("american samoa"[MeSH Terms] OR ("american"[All Fields] AND "samoa"[All Fields]) OR "american samoa"[All Fields])) OR ("samoa"[MeSH Terms] OR "samoa"[All Fields])) OR ("antigua and barbuda"[MeSH Terms] OR ("antigua"[All Fields] AND "barbuda"[All Fields]) OR "antigua and barbuda"[All Fields])) OR ("antigua and barbuda"[MeSH Terms] OR ("antigua"[All Fields] AND "barbuda"[All Fields]) OR "antigua and barbuda"[All Fields] OR "antigua"[All Fields])) OR ("antigua and barbuda"[MeSH Terms] OR ("antigua"[All Fields] AND "barbuda"[All Fields]) OR "antigua and barbuda"[All Fields] OR "barbuda"[All Fields])) OR ("argentina"[MeSH Terms] OR "argentina"[All Fields])) OR ("azerbaijan"[MeSH Terms] OR "azerbaijan"[All Fields])) OR ("republic of belarus"[MeSH Terms] OR ("republic"[All Fields] AND "belarus"[All Fields]) OR "republic of belarus"[All Fields] OR "belarus"[All Fields])) OR ("bosnia-herzegovina"[MeSH Terms] OR "bosnia-herzegovina"[All Fields] OR ("bosnia"[All Fields] AND "herzegovina"[All Fields]) OR "bosnia and herzegovina"[All Fields])) OR ("bosnia-herzegovina"[MeSH Terms] OR "bosnia-herzegovina"[All Fields] OR "bosnia"[All Fields])) OR ("bosnia-herzegovina"[MeSH Terms] OR "bosnia-herzegovina"[All Fields] OR "herzegovina"[All Fields])) OR ("botswana"[MeSH Terms] OR "botswana"[All Fields])) OR ("brazil"[MeSH Terms] OR "brazil"[All Fields])) OR ("bulgaria"[MeSH Terms] OR "bulgaria"[All Fields])) OR ("chile"[MeSH Terms] OR "chile"[All Fields])) OR ("china"[MeSH Terms] OR "china"[All Fields])) OR ("colombia"[MeSH Terms] OR "colombia"[All Fields])) OR ("costa rica"[MeSH Terms] OR ("costa"[All Fields] AND "rica"[All Fields]) OR "costa rica"[All Fields])) OR ("cuba"[MeSH Terms] OR "cuba"[All Fields])) OR ("dominica"[MeSH Terms] OR "dominica"[All Fields])) OR ("dominican republic"[MeSH Terms] OR ("dominican"[All Fields] AND "republic"[All Fields]) OR "dominican republic"[All Fields])) OR ("ecuador"[MeSH Terms] OR "ecuador"[All Fields])) OR ("gabon"[MeSH Terms] OR "gabon"[All Fields])) OR ("grenada"[MeSH Terms] OR "grenada"[All Fields])) OR ("iran"[MeSH Terms] OR "iran"[All Fields])) OR ("jamaica"[MeSH Terms] OR "jamaica"[All Fields])) OR ("jordan"[MeSH Terms] OR "jordan"[All Fields])) OR ("kazakhstan"[MeSH Terms] OR "kazakhstan"[All Fields])) OR ("latvia"[MeSH Terms] OR "latvia"[All Fields])) OR ("lebanon"[MeSH Terms] OR "lebanon"[All Fields])) OR ("libya"[MeSH Terms] OR "libya"[All Fields])) OR ("lithuania"[MeSH Terms] OR "lithuania"[All Fields])) OR ("macedonia (republic)"[MeSH Terms] OR ("macedonia"[All Fields] AND "(republic)"[All Fields]) OR "macedonia (republic)"[All Fields] OR "macedonia"[All Fields])) OR ("malaysia"[MeSH Terms] OR "malaysia"[All Fields])) OR ("indian ocean islands"[MeSH Terms] OR ("indian"[All Fields] AND "ocean"[All Fields] AND "islands"[All Fields]) OR "indian ocean islands"[All Fields] OR "maldives"[All Fields])) OR ("mauritius"[MeSH Terms] OR "mauritius"[All Fields])) OR ("comoros"[MeSH Terms] OR "comoros"[All Fields] OR "mayotte"[All Fields])) OR ("mexico"[MeSH Terms] OR "mexico"[All Fields])) OR ("montenegro"[MeSH Terms] OR "montenegro"[All Fields])) OR ("namibia"[MeSH Terms] OR "namibia"[All Fields])) OR ("palau"[MeSH Terms] OR "palau"[All Fields])) OR ("panama"[MeSH Terms] OR "panama"[All Fields])) OR ("peru"[MeSH Terms] OR "peru"[All Fields])) OR ("romania"[MeSH Terms] OR "romania"[All Fields])) OR ("russia"[MeSH Terms] OR "russia"[All Fields] OR ("russian"[All Fields] AND "federation"[All Fields]) OR "russian federation"[All Fields])) OR ("russia"[MeSH Terms] OR "russia"[All Fields])) OR ("ussr"[MeSH Terms] OR "ussr"[All Fields])) OR ("serbia"[MeSH Terms] OR "serbia"[All Fields])) OR ("seychelles"[MeSH Terms] OR "seychelles"[All Fields])) OR ("south africa"[MeSH Terms] OR ("south"[All Fields] AND "africa"[All Fields]) OR "south africa"[All Fields])) OR ("saint kitts and nevis"[MeSH Terms] OR ("saint"[All Fields] AND "kitts"[All Fields] AND "nevis"[All Fields]) OR "saint kitts and nevis"[All Fields] OR ("st"[All Fields] AND "kitts"[All Fields] AND "nevis"[All Fields]) OR "st kitts and nevis"[All Fields])) OR ("saint kitts and nevis"[MeSH Terms] OR ("saint"[All Fields] AND "kitts"[All Fields] AND "nevis"[All Fields]) OR "saint kitts and nevis"[All Fields])) OR (("saints"[MeSH Terms] OR "saints"[All Fields] OR "saint"[All Fields]) AND Kitts[All Fields])) OR ("saint lucia"[MeSH Terms] OR ("saint"[All Fields] AND "lucia"[All Fields]) OR "saint lucia"[All Fields] OR ("st"[All Fields] AND "lucia"[All Fields]) OR "st lucia"[All Fields])) OR ("saint lucia"[MeSH Terms] OR ("saint"[All Fields] AND "lucia"[All Fields]) OR "saint lucia"[All Fields])) OR ("saint vincent and the grenadines"[MeSH Terms] OR ("saint"[All Fields] AND "vincent"[All Fields] AND "grenadines"[All Fields]) OR "saint vincent and the grenadines"[All Fields] OR ("st"[All Fields] AND "vincent"[All Fields] AND "grenadines"[All Fields]) OR "st vincent and the grenadines"[All Fields])) OR (St. Vincent[Author] OR St. Vincent[Investigator])) OR ("saint vincent and the grenadines"[MeSH Terms] OR ("saint"[All Fields] AND "vincent"[All Fields] AND "grenadines"[All Fields]) OR "saint vincent and the grenadines"[All Fields])) OR Saint Vincent[Author]) OR ("saint vincent and the grenadines"[MeSH Terms] OR ("saint"[All Fields] AND "vincent"[All Fields] AND "grenadines"[All Fields]) OR "saint vincent and the grenadines"[All Fields] OR "grenadines"[All Fields])) OR ("suriname"[MeSH Terms] OR "suriname"[All Fields])) OR ("thailand"[MeSH Terms] OR "thailand"[All Fields])) OR ("tunisia"[MeSH Terms] OR "tunisia"[All Fields])) OR ("turkey"[MeSH Terms] OR "turkey"[All Fields])) OR ("uruguay"[MeSH Terms] OR "uruguay"[All Fields])) OR ("venezuela"[MeSH Terms] OR "venezuela"[All Fields]))))

CINAHL search strategy

#	Query	Limiters/Expanders
S44	S39 and S43	Search modes - Boolean/Phrase Limiters - Published Date from: 20000101-20121231
S43	S40 or S41 or S42	Search modes - Boolean/Phrase
S42	Albania OR Algeria OR Samoa OR Antigua OR Barbuda OR Argentina OR Azerbaijan OR Belarus OR Bosnia OR Herzegovina OR Botswana OR Brazil OR Bulgaria OR Chile OR China OR Colombia OR Costa Rica OR Cuba OR Dominica OR Dominican Republic OR Ecuador OR Gabon OR Grenada OR Iran OR Jamaica OR Jordan OR Kazakhstan OR Latvia OR Lebanon OR Libya OR Lithuania OR Macedonia OR Malaysia OR Maldives OR Mauritius OR Mayotte OR Mexico OR Montenegro OR Namibia OR Palau OR Panama OR Peru OR Romania OR Russian Federation OR SerbiaOR Seychelles OR South Africa OR St. Kitts and Nevis OR St. Lucia OR St. Vincent OR Grenadines OR Suriname OR Thailand OR Tunisia OR Turkey OR Uruguay OR Venezuela	Search modes - Boolean/Phrase
S41	Angola OR Armenia OR Belize OR Bhutan OR Bolivia OR Cameroon OR Cape Verde OR Congo, Rep OR Côte d'Ivoire OR Djibouti OR Egypt OR El Salvador OR Fiji OR Georgia OR Ghana OR Guatemala OR Guyana OR Honduras OR Indonesia OR India OR Iraq OR Kiribati OR Kosovo OR Lao PDR OR Lesotho OR Marshall Islands OR Mauritania OR Micronesia OR Moldova OR Mongolia OR Morocco OR Nicaragua OR Nigeria OR Pakistan OR Papua New Guinea OR Paraguay OR Philippines OR Samoa OR São Tomé and Principe OR Senegal OR Solomon Islands OR Sri Lanka OR Sudan OR Swaziland OR Syria* OR Timor-Leste OR Tonga OR Turkmenistan OR Tuvalu OR Ukraine OR Uzbekistan OR Vanuatu OR Vietnam OR Gaza OR Yemen OR Zambia	Search modes - Boolean/Phrase
S40	Afghanistan OR Bangladesh OR Benin OR Burkina Faso OR Burundi OR Cambodia OR Central African Republic OR Chad OR Comoros OR Congo, Dem. Rep OR Eritrea OR Ethiopia OR Gambia, The OR Guinea OR Guinea-Bisau OR Haiti OR Kenya OR Korea, Dem Rep OR Kyrgyz Republic OR Liberia OR Madagascar OR Malawi OR Mali OR Mozambique OR Myanmar OR Nepal OR Niger OR Rwanda OR Sierra Leone OR Somalia OR Tajikistan OR Tanzania OR Togo OR Uganda OR Zimbabwe	Search modes - Boolean/Phrase
S39	S8 or S13 or S19 or S25 or S38	Search modes - Boolean/Phrase
S38	S26 or S27 or S28 or S29 or S30 or S31 or S32 or S33 or S34 or S35 or S36 or S37	Search modes - Boolean/Phrase
S37	traditional birth attendant	Search modes - Boolean/Phrase
S36	(attend* OR unattend*) N2 (birth* OR delivery or labo#r)	Search modes - Boolean/Phrase
S35	unattended birth	Search modes - Boolean/Phrase
S34	(MH "Lay Midwives") OR "birth attendant"	Search modes - Boolean/Phrase
S33	(MH "Delivery Rooms") OR (MH "Alternative Birth Centers")	Search modes - Boolean/Phrase
S32	(MH "Pregnancy, Ectopic") OR (MH "Pregnancy Complications, Cardiovascular+") OR (MH "Pregnancy Complications, Neoplastic+") OR (MH "Puerperal Disorders+")	Search modes - Boolean/Phrase
S31	(MH "Intrapartum Care") OR (MH "Obstetric Care") OR (MH "Delivery")	Search modes - Boolean/Phrase
S30	MM "Management of Labor"	Search modes - Boolean/Phrase
S29	(pro#long* OR obstruct*) N2 (deliver* OR labo#r)	Search modes - Boolean/Phrase
S28	"obstructed labor"	Search modes - Boolean/Phrase
S27	Miscarriage	Search modes - Boolean/Phrase
S26	(MH "Abortion, Spontaneous")	Search modes - Boolean/Phrase
S25	S20 or S21 or S22 or S23 or S24	Search modes - Boolean/Phrase
S24	pre#eclampsia	Search modes - Boolean/Phrase
S23	HELLP	Search modes - Boolean/Phrase
S22	(MH "Eclampsia+") OR (MH "Pre-Eclampsia+")	Search modes - Boolean/Phrase
S21	Eclampsia	Search modes - Boolean/Phrase
S20	(MH "Pregnancy-Induced Hypertension")	Search modes - Boolean/Phrase
S19	S14 or S15 or S17 or S18	Search modes - Boolean/Phrase

S18	"post#partum h#emorrhage"	Search modes - Boolean/Phrase
S17	S2 N2 S16	Search modes - Boolean/Phrase
S16	h#emorrhage	Search modes - Boolean/Phrase
S15	(MH "Postpartum Hemorrhage")	Search modes - Boolean/Phrase
S14	postpartum hemorrhage	Search modes - Boolean/Phrase
S13	S9 or S10 or S12	Search modes - Boolean/Phrase
S12	S2 N2 S11	Search modes - Boolean/Phrase
S11	an#emia	Search modes - Boolean/Phrase
S10	MM "Pregnancy Complications, Hematologic"	Search modes - Boolean/Phrase
S9	maternal anemia	Search modes - Boolean/Phrase
S8	S1 or S4 or S5 or S6 or S7	Search modes - Boolean/Phrase
S7	MM "Pregnancy Complications, Infectious"	Search modes - Boolean/Phrase
S6	infection in pregnancy	Search modes - Boolean/Phrase
S5	"maternal infection"	Search modes - Boolean/Phrase
S4	S2 N2 S3	Search modes - Boolean/Phrase
S3	(infect* OR sepsis OR septic OR tubercul* OR pneumonia OR meningitis OR HIV)	Search modes - Boolean/Phrase
S2	(pregnan* OR maternal OR obstetric* OR puerper* OR partum OR birth OR childbirth)	Search modes - Boolean/Phrase
S1	(MH "Chorioamnionitis")	Search modes - Boolean/Phrase

Embase search strategy

1	(Albania or Algeria or Samoa or Antigua or Barbuda or Argentina or Azerbaijan or Belarus or Bosnia or Herzegovina or Botswana or Brazil or Bulgaria or Chile or China or Colombia or Costa Rica or Cuba or Dominica or Dominican Republic or Ecuador or Gabon or Grenada or Iran or Jamaica or Jordan or Kazakhstan or Latvia or Lebanon or Libya or Lithuania or Macedonia or Malaysia or Maldives or Mauritius or Mayotte or Mexico or Montenegro or Namibia or Palau or Panama or Peru or Romania or Russian Federation or Russia or Serbia or Seychelles or South Africa or St Kitts or Nevis or St Lucia or St Vincent or Grenadines or Suriname or Thailand or Tunisia or Turkey or Uruguay or Venezuela).mp. [mp=title, abstract, subject headings, heading word, drug trade name, original title, device manufacturer, drug manufacturer, device trade name, keyword]
2	(Angola or Armenia or Belize or Bhutan or Bolivia or Cameroon or Cape Verde or Congo, or Cote d'Ivoire or Ivory Coast or Djibouti or Egypt or Arab Republic or El Salvador or Fiji or Georgia or Ghana or Guatemala or Guyana or Honduras or Indonesia or India or Iraq or Kiribati or Kosovo or Lao PDR or Lesotho or Marshall Islands or Mauritania or Micronesia or Moldova or Mongolia or Morocco or Nicaragua or Nigeria or Pakistan or Papua New Guinea or Paraguay or Philippines or Samoa or Sao Tome or Principe or Senegal or Solomon Islands or Sri Lanka or Sudan or Swaziland or Syrian Arab Republic or Timor-Leste or Tonga or Turkmenistan or Tuvalu or Ukraine or Uzbekistan or Vanuatu or Vietnam or West Bank or Gaza or Yemen or Zambia).mp. [mp=title, abstract, subject headings, heading word, drug trade name, original title, device manufacturer, drug manufacturer, device trade name, keyword]
3	(Afghanistan or Bangladesh or Benin or Burkina Faso or Burundi or Cambodia or Central African Republic or Chad or Comoros or Congo or Eritrea or Ethiopia or Gambia or Guinea or Bisau or Haiti or Kenya or Korea or Kyrgyz or Liberia or Madagascar or Malawi or Mali or Mozambique or Myanmar or Nepal or Niger or Rwanda or Sierra Leone or Somalia or Tajikistan or Tanzania or Togo or Uganda or Zimbabwe).mp. [mp=title, abstract, subject headings, heading word, drug trade name, original title, device manufacturer, drug manufacturer, device trade name, keyword]
4	(Africa or sahara* or low income country or low income countries or middle income country or middle income countries or south america or central america or latin america or carribean).mp. [mp=title, abstract, subject headings, heading word, drug trade name, original title, device manufacturer, drug manufacturer, device trade name, keyword]
5	exp Developing Countries/
6	(#1 or #2 or #3 or #4 or #5).mp. [mp=title, abstract, subject headings, heading word, drug trade name, original title, device manufacturer, drug manufacturer, device trade name, keyword]
7	limit 6 to (human and yr="2000 -Current")
8	maternal infection.mp.
9	chorioamnionitis.mp.
10	exp maternal disease/ or exp intrauterine infection/
11	(pregnan* or maternal or obstetric* or puerper* or partum or birth or childbirth or prenatal or postnatal or natal).mp. [mp=title, abstract, subject headings, heading word, drug trade name, original title, device manufacturer, drug manufacturer, device trade name, keyword]
12	(infect* or sepsis or septic or tubercul* or pneumonia or meningitis or HIV).mp. [mp=title, abstract, subject headings, heading word, drug trade name, original title, device manufacturer, drug manufacturer, device trade name, keyword]
13	(#11 adj3 #12).mp. [mp=title, abstract, subject headings, heading word, drug trade name, original title, device manufacturer, drug manufacturer, device trade name, keyword]
14	maternal anemia.mp.
15	exp PREGNANCY COMPLICATIONS, HEMATOLOGIC/
16	(anemi* or anaemi* or hemoglobin or haemoglobin).mp. [mp=title, abstract, subject headings, heading word, drug trade name, original title, device manufacturer, drug manufacturer, device trade name, keyword]
17	(#11 adj3 #16).mp. [mp=title, abstract, subject headings, heading word, drug trade name, original title, device manufacturer, drug manufacturer, device trade name, keyword]
18	exp postpartum hemorrhage/
19	((maternal or obstetric* or puerper* or partum or birth or childbirth or postnatal) adj2 (bleed or bleeding or hemorrhage or haemorrhage)).mp. [mp=title, abstract, subject headings, heading word, drug trade name, original title, device manufacturer, drug manufacturer, device trade name, keyword]
20	exp "eclampsia and preeclampsia"/ or exp eclampsia/
21	(eclampsia or pre-ecalmapsia or preeclampsia or HELLP).ti,ab.
22	miscarriage.ti,ab.
23	exp SPONTANEOUS ABORTION/
24	obstructed labor.mp.
25	exp LABOR OBSTRUCTION/

26 ((obstruct* or prolong*) adj2 (labour or labor or delivery or birth or childbirth)).ti,ab.

27 exp LABOR MANAGEMENT/

28 exp intrapartum care/

29 exp perinatal care/

30 exp DELIVERY ROOM/

31 exp HOME DELIVERY/

32 exp birthplace/

33 birth attendant*.mp.

34 place* of birth*.mp.

35 ((attend* or unattend* or alone or support) adj2 (Birth* or childbirth* or deliver*)).ti,ab.

36 *MATERNAL CARE/

37 8 or 9 or 10 or 13 or 14 or 15 or 17 or 18 or 19 or 20 or 21 or 22 or 23 or 24 or 25 or 26 or 27 or 28 or 29 or 30 or 31 or 32 or 33 or 34 or 35 or 36

38 clincal trial.mp.

39 phase 1 clinical trial/

407 phase 2 clinical trial/

41 controlled clinical trial/ or clinical trial/ or "controlled clinical trial (topic)"/

42 phase 3 clinical trial/

43 phase 4 clinical trial/

44 38 or 39 or 40 or 41 or 42 or 43

45 7 and 37

46 limit 45 to ((evidence based medicine or meta analysis or outcomes research or "systematic review") and yr="2000 -Current")

PsycINFO search strategy

1 (Albania or Algeria or Samoa or Antigua or Barbuda or Argentina or Azerbaijan or Belarus or Bosnia or Herzegovina or Botswana or Brazil or Bulgaria or Chile or China or Colombia or Costa Rica or Cuba or Dominica or Dominican Republic or Ecuador or Gabon or Grenada or Iran or Jamaica or Jordan or Kazakhstan or Latvia or Lebanon or Libya or Lithuania or Macedonia or Malaysia or Maldives or Mauritius or Mayotte or Mexico or Montenegro or Namibia or Palau or Panama or Peru or Romania or Russian Federation or Russia or Serbia or Seychelles or South Africa or St Kitts or Nevis or St Lucia or St Vincent or Grenadines or Suriname or Thailand or Tunisia or Turkey or Uruguay or Venezuela).mp. [mp=title, abstract, heading word, table of contents, key concepts, original title, tests & measures] (51248)
2 (Angola or Armenia or Belize or Bhutan or Bolivia or Cameroon or Cape Verde or Congo, or Cote d'Ivoire or Ivory Coast or Djibouti or Egypt or Arab Republic or El Salvador or Fiji or Georgia or Ghana or Guatemala or Guyana or Honduras or Indonesia or India or Iraq or Kiribati or Kosovo or Lao PDR or Lesotho or Marshall Islands or Mauritania or Micronesia or Moldova or Mongolia or Morocco or Nicaragua or Nigeria or Pakistan or Papua New Guinea or Paraguay or Philippines or Samoa or Sao Tome or Principe or Senegal or Solomon Islands or Sri Lanka or Sudan or Swaziland or Syrian Arab Republic or Timor-Leste or Tonga or Turkmenistan or Tuvalu or Ukraine or Uzbekistan or Vanuatu or Vietnam or West Bank or Gaza or Yemen or Zambia).mp. [mp=title, abstract, heading word, table of contents, key concepts, original title, tests & measures] (28159)
3 (Afghanistan or Bangladesh or Benin or Burkina Faso or Burundi or Cambodia or Central African Republic or Chad or Comoros or Congo or Eritrea or Ethiopia or Gambia or Guinea or Bisau or Haiti or Kenya or Korea or Kyrgyz or Liberia or Madagascar or Malawi or Mali or Mozambique or Myanmar or Nepal or Niger or Rwanda or Sierra Leone or Somalia or Tajikistan or Tanzania or Togo or Uganda or Zimbabwe).mp. [mp=title, abstract, heading word, table of contents, key concepts, original title, tests & measures] (15936)
4 (Africa or sahara* or low income country or low income countries or middle income country or middle income countries or south america or central america or latin america or carribean).mp. [mp=title, abstract, heading word, table of contents, key concepts, original title, tests & measures] (13920)
5 exp Developing Countries/ (3010)
6 limit 2 to (human and yr="2000 - 2012") (18992)
7 limit 3 to (human and yr="2000 - 2012") (10958)
8 limit 4 to (human and yr="2000 - 2012") (10674)
9 limit 5 to (human and yr="2000 - 2012") (2363)
10 (#1 or #2 or #3 or #4 or #5).mp. [mp=title, abstract, heading word, table of contents, key concepts, original title, tests & measures] (906414)
11 limit 10 to (human and yr="2000 -Current") (510379)
12 maternal infection.mp. (118)
13 chorioamnionitis.mp. (40)
14 exp midwifery/ or exp obstetrical complications/ (1531)
15 miscarriage.mp. or exp Spontaneous Abortion/ (768)
16 (pregnan* or maternal or obstetric* or puerper* or partum or birth or childbirth or prenatal or postnatal or natal or post-partum).mp. [mp=title, abstract, heading word, table of contents, key concepts, original title, tests & measures] (84865)
17 (infect* or sepsis or septic or tubercul* or pneumonia or meningitis or HIV or hemorrhage or haemorrhage or bleed*).mp. [mp=title, abstract, heading word, table of contents, key concepts, original title, tests & measures] (49913)
18 (#18 adj3 #19).mp. [mp=title, abstract, heading word, table of contents, key concepts, original title, tests & measures] (2516)
19 (anemia or anaemia).mp. [mp=title, abstract, heading word, table of contents, key concepts, original title, tests & measures] (1011)
20 (#18 adj3 #21).mp. [mp=title, abstract, heading word, table of contents, key concepts, original title, tests & measures] (1230)
21 ((obstruc* or prolong*) adj3 (labour or labour or birth or delivery)).mp. [mp=title, abstract, heading word, table of contents, key concepts, original title, tests & measures] (43)
22 birth attendant*.mp. [mp=title, abstract, heading word, table of contents, key concepts, original title, tests & measures] (110)
23 childbirth.mp. [mp=title, abstract, heading word, table of contents, key concepts, original title, tests & measures] (2867)
24 *Birth/ (2853)
25 12 or 13 or 14 or 15 or 18 or 20 or 21 or 22 or 23 or 24 (10515)
26 11 and 25 (4257)
27 limit 26 to (human and ("reviews (maximizes sensitivity)" or "therapy (maximizes sensitivity)" or "qualitative (maximizes sensitivity)") and human and yr="2000 -Current") (3171)
28 limit 27 to (120 neonatal <birth to age 1 mo> or 200 adolescence <age 13 to 17 yrs> or 320 young adulthood <age 18 to 29 yrs> or 340 thirties <age 30 to 39 yrs> or 360 middle age <age 40 to 64 yrs>) (1139)
29 limit 28 to (100 childhood <birth to age 12 yrs> or 200 adolescence <age 13 to 17 yrs> or 320 young adulthood <age 18 to 29 yrs> or 340 thirties <age 30 to 39 yrs> or 360 middle age <age 40 to 64 yrs>) (1139)
30 limit 29 to yr="2000 - 2005" (339)
31 limit 29 to yr="2006 -Current" (800)

Web of Knowledge search strategy

Set	Results	Search terms
# 69	**8,903**	#59 AND #6<br>Refined by: Web of Science Categories=(PUBLIC ENVIRONMENTAL OCCUPATIONAL HEALTH OR INFECTIOUS DISEASES OR OBSTETRICS GYNECOLOGY OR MEDICINE GENERAL INTERNAL OR TROPICAL MEDICINE OR SOCIAL SCIENCES BIOMEDICAL OR HEALTH POLICY SERVICES OR NURSING OR MEDICINE RESEARCH EXPERIMENTAL)<br>*Databases=SCI-EXPANDED, SSCI, A&HCI Timespan=2000-01-01 - 2012-09-21*<br>*Lemmatization=On*
# 68	**1,248**	#59 AND #6<br>Refined by: Web of Science Categories=(MEDICINE GENERAL INTERNAL)<br>*Databases=SCI-EXPANDED, SSCI, A&HCI Timespan=2000-01-01 - 2012-09-21*<br>*Lemmatization=On*
# 67	**3,064**	#59 AND #6<br>Refined by: Web of Science Categories=(PUBLIC ENVIRONMENTAL OCCUPATIONAL HEALTH)<br>*Databases=SCI-EXPANDED, SSCI, A&HCI Timespan=2000-01-01 - 2012-09-21*<br>*Lemmatization=On*

# 66	**1,560**	#59 AND #6<br>Refined by: Web of Science Categories=(IMMUNOLOGY)<br>*Databases=SCI-EXPANDED, SSCI, A&HCI Timespan=2000-01-01 - 2012-09-21*<br>*Lemmatization=On*
# 65	**2,080**	#59 AND #6<br>Refined by: Web of Science Categories=(INFECTIOUS DISEASES)<br>*Databases=SCI-EXPANDED, SSCI, A&HCI Timespan=2000-01-01 - 2012-09-21*<br>*Lemmatization=On*
# 64	**10,555**	#59 AND #6<br>Refined by: Web of Science Categories=(PUBLIC ENVIRONMENTAL OCCUPATIONAL HEALTH OR INFECTIOUS DISEASES OR OBSTETRICS GYNECOLOGY OR IMMUNOLOGY OR MEDICINE GENERAL INTERNAL OR TROPICAL MEDICINE OR PEDIATRICS OR VIROLOGY OR MICROBIOLOGY OR SOCIAL SCIENCES BIOMEDICAL OR PARASITOLOGY OR HEALTH POLICY SERVICES OR NUTRITION DIETETICS OR NURSING OR MEDICINE RESEARCH EXPERIMENTAL OR HEALTH CARE SCIENCES SERVICES OR REPRODUCTIVE BIOLOGY)<br>*Databases=SCI-EXPANDED, SSCI, A&HCI Timespan=2000-01-01 - 2012-09-21*<br>*Lemmatization=On*
# 63	**10,852**	#59 AND #6<br>Refined by: Web of Science Categories=(PUBLIC ENVIRONMENTAL OCCUPATIONAL HEALTH OR INFECTIOUS DISEASES OR OBSTETRICS GYNECOLOGY OR IMMUNOLOGY OR WOMEN S STUDIES OR MEDICINE GENERAL INTERNAL OR TROPICAL MEDICINE OR PEDIATRICS OR VIROLOGY OR MICROBIOLOGY OR SOCIAL SCIENCES BIOMEDICAL OR PARASITOLOGY OR HEALTH POLICY SERVICES OR NUTRITION DIETETICS OR NURSING OR MEDICINE RESEARCH EXPERIMENTAL OR BIOLOGY OR RESPIRATORY SYSTEM OR HEALTH CARE SCIENCES SERVICES OR DEMOGRAPHY OR ENVIRONMENTAL SCIENCES OR PSYCHOLOGY MULTIDISCIPLINARY OR REPRODUCTIVE BIOLOGY OR SURGERY) AND Research Areas=(PUBLIC ENVIRONMENTAL OCCUPATIONAL HEALTH OR INFECTIOUS DISEASES OR OBSTETRICS GYNECOLOGY OR IMMUNOLOGY OR GENERAL INTERNAL MEDICINE OR TROPICAL MEDICINE OR PEDIATRICS OR VIROLOGY OR MICROBIOLOGY OR BIOMEDICAL SOCIAL SCIENCES OR HEALTH CARE SCIENCES SERVICES OR PARASITOLOGY OR NUTRITION DIETETICS OR NURSING OR RESEARCH EXPERIMENTAL MEDICINE OR LIFE SCIENCES BIOMEDICINE OTHER TOPICS OR REPRODUCTIVE BIOLOGY OR WOMEN S STUDIES)<br>*Databases=SCI-EXPANDED, SSCI, A&HCI Timespan=2000-01-01 - 2012-09-21*<br>*Lemmatization=On*
# 62	**11,111**	#59 AND #6<br>Refined by: Web of Science Categories=(PUBLIC ENVIRONMENTAL OCCUPATIONAL HEALTH OR INFECTIOUS DISEASES OR OBSTETRICS GYNECOLOGY OR IMMUNOLOGY OR WOMEN S STUDIES OR MEDICINE GENERAL INTERNAL OR TROPICAL MEDICINE OR PEDIATRICS OR VIROLOGY OR MICROBIOLOGY OR SOCIAL SCIENCES BIOMEDICAL OR PARASITOLOGY OR HEALTH POLICY SERVICES OR NUTRITION DIETETICS OR NURSING OR MEDICINE RESEARCH EXPERIMENTAL OR BIOLOGY OR RESPIRATORY SYSTEM OR HEALTH CARE SCIENCES SERVICES OR DEMOGRAPHY OR ENVIRONMENTAL SCIENCES OR PSYCHOLOGY MULTIDISCIPLINARY OR REPRODUCTIVE BIOLOGY OR SURGERY)<br>*Databases=SCI-EXPANDED, SSCI, A&HCI Timespan=2000-01-01 - 2012-09-21*<br>*Lemmatization=On*
# 61	**5,217**	#59 AND #6<br>Refined by: Web of Science Categories=(OBSTETRICS GYNECOLOGY OR WOMEN S STUDIES OR MEDICINE GENERAL INTERNAL OR HEMATOLOGY OR TROPICAL MEDICINE OR PEDIATRICS OR SOCIAL SCIENCES BIOMEDICAL OR SOCIAL SCIENCES INTERDISCIPLINARY OR SOCIOLOGY OR SOCIAL ISSUES)<br>*Databases=SCI-EXPANDED, SSCI, A&HCI Timespan=2000-01-01 - 2012-09-21*<br>*Lemmatization=On*
# 60	**13,054**	#59 AND #6<br>*Databases=SCI-EXPANDED, SSCI, A&HCI Timespan=2000-01-01 - 2012-09-21*<br>*Lemmatization=On*
# 59	**95,097**	#58 OR #33 OR #29 OR #22 OR #18<br>*Databases=SCI-EXPANDED, SSCI, A&HCI Timespan=2000-01-01 - 2012-09-21*<br>*Lemmatization=On*
# 58	**37,609**	#57 OR #56 OR #55 OR #54 OR #53 OR #52 OR #51 OR #47 OR #46 OR #45 OR #44 OR #43 OR #42 OR #41 OR #40 OR #39 OR #38 OR #37 OR #36 OR #35 OR #34<br>*Databases=SCI-EXPANDED, SSCI, A&HCI Timespan=2000-01-01 - 2012-09-21*<br>*Lemmatization=On*
# 57	**395**	Topic=((*attend* childbirth*))<br>*Databases=SCI-EXPANDED, SSCI, A&HCI Timespan=2000-01-01 - 2012-09-21*<br>*Lemmatization=On*
# 56	**2,833**	Topic=((*attend* birth))<br>*Databases=SCI-EXPANDED, SSCI, A&HCI Timespan=2000-01-01 - 2012-09-21*<br>*Lemmatization=On*
# 55	**1,592**	Topic=("maternal care")<br>*Databases=SCI-EXPANDED, SSCI, A&HCI Timespan=2000-01-01 - 2012-09-21*<br>*Lemmatization=On*
# 54	**3,944**	Topic=(midwife)<br>*Databases=SCI-EXPANDED, SSCI, A&HCI Timespan=2000-01-01 - 2012-09-21*<br>*Lemmatization=On*
# 53	**529**	Topic=("birth attendant*")<br>*Databases=SCI-EXPANDED, SSCI, A&HCI Timespan=2000-01-01 - 2012-09-21*<br>*Lemmatization=On*
# 52	**587**	Topic=("place of birth")<br>*Databases=SCI-EXPANDED, SSCI, A&HCI Timespan=2000-01-01 - 2012-09-21*<br>*Lemmatization=On*

# 51	**2,632**	#13 AND #50<br>*Databases=SCI-EXPANDED, SSCI, A&HCI Timespan=2000-01-01 - 2012-09-21*<br>*Lemmatization=On*
# 50	**8,104**	#49 OR #48<br>*Databases=SCI-EXPANDED, SSCI, A&HCI Timespan=2000-01-01 - 2012-09-21*<br>*Lemmatization=On*
# 49	**8,104**	Topic=((labour management))<br>*Databases=SCI-EXPANDED, SSCI, A&HCI Timespan=2000-01-01 - 2012-09-21*<br>*Lemmatization=On*
# 48	**8,104**	Topic=((labor management))<br>*Databases=SCI-EXPANDED, SSCI, A&HCI Timespan=2000-01-01 - 2012-09-21*<br>*Lemmatization=On*
# 47	**1,002**	Topic=("obstetric care")<br>*Databases=SCI-EXPANDED, SSCI, A&HCI Timespan=2000-01-01 - 2012-09-21*<br>*Lemmatization=On*
# 46	**195**	Topic=("intrapartum care")<br>*Databases=SCI-EXPANDED, SSCI, A&HCI Timespan=2000-01-01 - 2012-09-21*<br>*Lemmatization=On*
# 45	**17,221**	Topic=(pregnan* complicat*)<br>*Databases=SCI-EXPANDED, SSCI, A&HCI Timespan=2000-01-01 - 2012-09-21*<br>*Lemmatization=On*
# 44	**7**	Topic=("complication* of labour")<br>*Databases=SCI-EXPANDED, SSCI, A&HCI Timespan=2000-01-01 - 2012-09-21*<br>*Lemmatization=On*
# 43	**32**	Topic=("complication* of labor")<br>*Databases=SCI-EXPANDED, SSCI, A&HCI Timespan=2000-01-01 - 2012-09-21*<br>*Lemmatization=On*
# 42	**2,810**	Topic=(complication* of labor)<br>*Databases=SCI-EXPANDED, SSCI, A&HCI Timespan=2000-01-01 - 2012-09-21*<br>*Lemmatization=On*
# 41	**1,755**	Topic=(dystocia)<br>*Databases=SCI-EXPANDED, SSCI, A&HCI Timespan=2000-01-01 - 2012-09-21*<br>*Lemmatization=On*
# 40	**465**	Topic=(OBSTRUCT* LABOUR)<br>*Databases=SCI-EXPANDED, SSCI, A&HCI Timespan=2000-01-01 - 2012-09-21*<br>*Lemmatization=On*
# 39	**1,223**	Topic=(PROLONG* LABOUR)<br>*Databases=SCI-EXPANDED, SSCI, A&HCI Timespan=2000-01-01 - 2012-09-21*<br>*Lemmatization=On*
# 38	**1,223**	Topic=(PROLONG* LABOR)<br>*Databases=SCI-EXPANDED, SSCI, A&HCI Timespan=2000-01-01 - 2012-09-21*<br>*Lemmatization=On*
# 37	**465**	Topic=(OBSTRUCT* LABOR)<br>*Databases=SCI-EXPANDED, SSCI, A&HCI Timespan=2000-01-01 - 2012-09-21*<br>*Lemmatization=On*
# 36	**1,476**	Topic=("SPONTANEOUS ABORTIONS")<br>*Databases=SCI-EXPANDED, SSCI, A&HCI Timespan=2000-01-01 - 2012-09-21*<br>*Lemmatization=On*
# 35	**3,363**	Topic=("SPONTANEOUS ABORTION")<br>*Databases=SCI-EXPANDED, SSCI, A&HCI Timespan=2000-01-01 - 2012-09-21*<br>*Lemmatization=On*
# 34	**4,380**	Topic=(miscarriage*) AND Topic=(pregnan*)<br>*Databases=SCI-EXPANDED, SSCI, A&HCI Timespan=2000-01-01 - 2012-09-21*<br>*Lemmatization=On*
# 33	**19,698**	#32 OR #31 OR #30<br>*Databases=SCI-EXPANDED, SSCI, A&HCI Timespan=2000-01-01 - 2012-09-21*<br>*Lemmatization=On*
# 32	**8,922**	Topic=(hypertens*) AND Topic=(pregnan*)<br>*Databases=SCI-EXPANDED, SSCI, A&HCI Timespan=2000-01-01 - 2012-09-21*<br>*Lemmatization=On*
# 31	**1,158**	Topic=(HELLP)<br>*Databases=SCI-EXPANDED, SSCI, A&HCI Timespan=2000-01-01 - 2012-09-21*<br>*Lemmatization=On*
# 30	**15,082**	Topic=(*eclampsia*)<br>*Databases=SCI-EXPANDED, SSCI, A&HCI Timespan=2000-01-01 - 2012-09-21*<br>*Lemmatization=On*
# 29	**2,959**	#28 OR #27 OR #26 OR #25 OR #24 OR #23

		Databases=SCI-EXPANDED, SSCI, A&HCI Timespan=2000-01-01 - 2012-09-21<br>*Lemmatization=On*
# 28	**802**	Topic=(*natal* h?emorrhage)<br>*Databases=SCI-EXPANDED, SSCI, A&HCI Timespan=2000-01-01 - 2012-09-21*<br>*Lemmatization=On*
# 27	**479**	Topic=(obstetric h?emorrhage)<br>*Databases=SCI-EXPANDED, SSCI, A&HCI Timespan=2000-01-01 - 2012-09-21*<br>*Lemmatization=On*
# 26	**0**	Topic=(obstetric h?emorhage)<br>*Databases=SCI-EXPANDED, SSCI, A&HCI Timespan=2000-01-01 - 2012-09-21*<br>*Lemmatization=On*
# 25	**697**	Topic=(postpartum bleed*)<br>*Databases=SCI-EXPANDED, SSCI, A&HCI Timespan=2000-01-01 - 2012-09-21*<br>*Lemmatization=On*
# 24	**510**	Topic=("postpartum haemorrhage".)<br>*Databases=SCI-EXPANDED, SSCI, A&HCI Timespan=2000-01-01 - 2012-09-21*<br>*Lemmatization=On*
# 23	**1,353**	Topic=("postpartum hemorrhage".)<br>*Databases=SCI-EXPANDED, SSCI, A&HCI Timespan=2000-01-01 - 2012-09-21*<br>*Lemmatization=On*
# 22	**1,835**	#21 OR #19<br>*Databases=SCI-EXPANDED, SSCI, A&HCI Timespan=2000-01-01 - 2012-09-21*<br>*Lemmatization=On*
# 21	**1,835**	#13 AND #20<br>*Databases=SCI-EXPANDED, SSCI, A&HCI Timespan=2000-01-01 - 2012-09-21*<br>*Lemmatization=On*
# 20	**18,960**	Topic=(an?emi* OR h?emoglobin)<br>*Databases=SCI-EXPANDED, SSCI, A&HCI Timespan=2000-01-01 - 2012-09-21*<br>*Lemmatization=On*
# 19	**113**	Topic=("maternal an?emia")<br>*Databases=SCI-EXPANDED, SSCI, A&HCI Timespan=2000-01-01 - 2012-09-21*<br>*Lemmatization=On*
# 18	**45,324**	#17 OR #16 OR #15 OR #12 OR #10 OR #9 OR #8 OR #7<br>*Databases=SCI-EXPANDED, SSCI, A&HCI Timespan=2000-01-01 - 2012-09-21*<br>*Lemmatization=On*
# 17	**43**	Topic=("PUERPERAL INFECTION")<br>*Databases=SCI-EXPANDED, SSCI, A&HCI Timespan=2000-01-01 - 2012-09-21*<br>*Lemmatization=On*
# 16	**246**	Topic=("infection in pregnancy")<br>*Databases=SCI-EXPANDED, SSCI, A&HCI Timespan=2000-01-01 - 2012-09-21*<br>*Lemmatization=On*
# 15	**43,468**	#14 AND #13<br>*Databases=SCI-EXPANDED, SSCI, A&HCI Timespan=2000-01-01 - 2012-09-21*<br>*Lemmatization=On*
# 14	**819,451**	Topic=(infect* OR sepsis OR septic OR tubercul* OR pneumonia OR meningitis OR HIV)<br>*Databases=SCI-EXPANDED, SSCI, A&HCI Timespan=2000-01-01 - 2012-09-21*<br>*Lemmatization=On*
# 13	**417,789**	Topic=(pregnan* OR maternal OR obstetric* OR puerper* OR partum OR birth OR childbirth OR prenatal OR postnatal OR *natal*)<br>*Databases=SCI-EXPANDED, SSCI, A&HCI Timespan=2000-01-01 - 2012-09-21*<br>*Lemmatization=On*
# 12	**925**	Topic=("INTRAUTERINE INFECTION")<br>*Databases=SCI-EXPANDED, SSCI, A&HCI Timespan=2000-01-01 - 2012-09-21*<br>*Lemmatization=On*
# 11	**2,514**	Topic=(INTRAUTERINE INFECTION)<br>*Databases=SCI-EXPANDED, SSCI, A&HCI Timespan=2000-01-01 - 2012-09-21*<br>*Lemmatization=On*
# 10	**1,013**	Topic=(FEMALE GENITAL TRACT INFECTION)<br>*Databases=SCI-EXPANDED, SSCI, A&HCI Timespan=2000-01-01 - 2012-09-21*<br>*Lemmatization=On*
# 9	**155**	Topic=(FEMALE GENITAL TRACT INFLAMMATION)<br>*Databases=SCI-EXPANDED, SSCI, A&HCI Timespan=2000-01-01 - 2012-09-21*<br>*Lemmatization=On*
# 8	**1,821**	Topic=(chorioamnionitis)<br>*Databases=SCI-EXPANDED, SSCI, A&HCI Timespan=2000-01-01 - 2012-09-21*<br>*Lemmatization=On*
# 7	**7,405**	Topic=(maternal infection*)

		Databases=SCI-EXPANDED, SSCI, A&HCI Timespan=2000-01-01 - 2012-09-21<br>*Lemmatization=On*
# 6	**848,085**	#5<br>*Databases=SCI-EXPANDED, SSCI, A&HCI Timespan=2000-01-01 - 2012-09-21*<br>*Lemmatization=On*
# 5	**1,375,550**	#4 OR #3 OR #2 OR #1<br>*Databases=SCI-EXPANDED, SSCI, A&HCI Timespan=All Years*<br>*Lemmatization=On*
# 4	**217,559**	Topic=(Africa OR *sahara* OR "low income country" OR "low income countries" OR "middle income country" OR "middle income countries" OR "south america" OR "central america" OR "latin america" OR carribean)<br>*Databases=SCI-EXPANDED, SSCI, A&HCI Timespan=All Years*<br>*Lemmatization=On*
# 3	**264,996**	Topic=(Afghanistan OR Bangladesh OR Benin OR Burkina Faso OR Burundi OR Cambodia OR Central African Republic OR Chad OR Comoros OR Congo, Dem. Rep OR Eritrea OR Ethiopia OR Gambia, The OR Guinea OR Guinea-Bisau OR Haiti OR Kenya OR Korea, Dem Rep OR Kyrgyz Republic OR Liberia OR Madagascar OR Malawi OR Mali OR Mozambique OR Myanmar OR Nepal OR Niger OR Rwanda OR Sierra Leone OR Somalia OR Tajikistan OR Tanzania OR Togo OR Uganda OR Zimbabwe)<br>*Databases=SCI-EXPANDED, SSCI, A&HCI Timespan=All Years*<br>*Lemmatization=On*
# 2	**380,742**	Topic=(Angola OR Armenia OR Belize OR Bhutan OR Bolivia OR Cameroon OR Cape Verde OR Congo, Rep OR C??te d'Ivoire OR Djibouti OR Egypt OR El Salvador OR Fiji OR Georgia OR Ghana OR Guatemala OR Guyana OR Honduras OR Indonesia OR India OR Iraq OR Kiribati OR Kosovo OR Lao PDR OR Lesotho OR Marshall Islands OR Mauritania OR Micronesia OR Moldova OR Mongolia OR Morocco OR Nicaragua OR Nigeria OR Pakistan OR Papua New Guinea OR Paraguay OR Philippines OR Samoa OR S??o Tom?? and Principe OR Senegal OR Solomon Islands OR Sri Lanka OR Sudan OR Swaziland OR Syria* OR Timor-Leste OR Tonga OR Turkmenistan OR Tuvalu OR Ukraine OR Uzbekistan OR Vanuatu OR Vietnam OR Gaza OR Yemen OR Zambia)<br>*Databases=SCI-EXPANDED, SSCI, A&HCI Timespan=All Years*<br>*Lemmatization=On*
# 1	**732,634**	Topic=(Albania OR Algeria OR Samoa OR Antigua OR Barbuda OR Argentina OR Azerbaijan OR Belarus OR Bosnia OR Herzegovina OR Botswana OR Brazil OR Bulgaria OR Chile OR China OR Colombia OR Costa Rica OR Cuba OR Dominica OR Dominican Republic OR Ecuador OR Gabon OR Grenada OR Iran OR Jamaica OR Jordan OR Kazakhstan OR Latvia OR Lebanon OR Libya OR Lithuania OR Macedonia OR Malaysia OR Maldives OR Mauritius OR Mayotte OR Mexico OR Montenegro OR Namibia OR Palau OR Panama OR Peru OR Romania OR Russian Federation OR SerbiaOR Seychelles OR South Africa OR St. Kitts and Nevis OR St. Lucia OR St. Vincent OR Grenadines OR Suriname OR Thailand OR Tunisia OR Turkey OR Uruguay OR Venezuela)<br>*Databases=SCI-EXPANDED, SSCI, A&HCI Timespan=All Years*<br>*Lemmatization=On*

.

Popline search strategy

This database does not allow complicated searching. Searches are limited to 1 line with limited Boolean options. The subject search option was therefore used as this has a range of maternal and child health options . Phrases and subjects are listed below

1 Subject pregnancy and childbirth complications
2. Safe motherhood
3. “postpartum hemorrhage”
4. Antenatal care –
5. Post-partum care – post partum women
6. Maternal care (limits -– developing countries – health services)
7. Maternal care (limits– developing countries – delivery of health services)
8. Maternal care (limits treatment – developing countries)
9. Maternal care (limits evaluation – developing countries)
10. Maternal mortality (limits developing countries)
11. Post-partum care
12. Contraception for post-partum women
13. Early detection (limits developing countries)
14. Danger signs (limits developing countries and socioeconomic factors)

LILACS

A combination of search terms were used all of which were limited to items about pregnancy. Search terms were: anemia or anaemia, birth attendant, hemorrhage or haemorrhage, infections terms, intrauterine infection, intrapartum care, maternal infection, maternal mortality, miscarriage, pre-eclampsia.

Other sources

Not all databases and registers of research allow the user to use sophisticated approaches for searching. Some may not use index terms, and incorporate limited options for combining terms and search strings. They may also be limited in the manner in which search results can be saved, if at all. It is therefore more difficult to record search strategies and results of screening for these sources

Annex 2. List of low- and middle-income countries

http://data.worldbank.org/about/country-classifications/country-and-lending-groups (7)

East Asia and Pacific		
American Samoa	Malaysia	Samoa
Cambodia	Marshall Islands	Solomon Islands
China	Micronesia, Fed. Sts	Thailand
Fiji	Mongolia	Timor-Leste
Indonesia	Myanmar	Tuvalu
Kiribati	Palau	Tonga
Korea, Dem. Rep.	Papua New Guinea	Vanuatu
Lao PDR	Philippines	Vietnam
**Europe and Central Asia**		
Albania	Kosovo	Russian Federation
Armenia	Kyrgyz Republic	Serbia
Azerbaijan	Latvia	Tajikistan
Belarus	Lithuania	Turkey
Bosnia and Herzegovina	Macedonia, FYR	Turkmenistan
Bulgaria	Moldova	Ukraine
Georgia	Montenegro	Uzbekistan
Kazakhstan	Romania	
**Latin America and the Caribbean**		
Antigua and Barbuda	Dominican Republic	Nicaragua
Argentina	Ecuador	Panama
Belize	El Salvador	Paraguay
Bolivia	Grenada	Peru
Brazil	Guatemala	St. Kitts and Nevis
Chile	Guyana	St. Lucia
Colombia	Haiti	St. Vincent and the Grenadines
Costa Rica	Honduras	Suriname
Cuba	Jamaica	Uruguay
Dominica	Mexico	Venezuela, RB
**Middle East and North Africa**		
Algeria	Jordan	Tunisia
Djibouti	Lebanon	West Bank and Gaza
Egypt, Arab Rep.	Libya	Yemen, Rep.
Iran, Islamic Rep.	Morocco	
Iraq	Syrian Arab Republic	
**South Asia**		
Afghanistan	India	Pakistan
Bangladesh	Maldives	Sri Lanka
Bhutan	Nepal	
**Sub-Saharan Africa**		
Angola	Gambia, The	Nigeria
Benin	Ghana	Rwanda
Botswana	Guinea	São Tomé and Principe
Burkina Faso	Guinea-Bissau	Senegal
Burundi	Kenya	Seychelles
Cameroon	Lesotho	Sierra Leone
Cape Verde	Liberia	Somalia
Central African Republic	Madagascar	South Africa
Chad	Malawi	South Sudan
Comoros	Mali	Sudan
Congo, Dem. Rep.	Mauritania	Swaziland
Congo, Rep	Mauritius	Tanzania
Côte d'Ivoire	Mayotte	Togo

Eritrea	Mozambique	Uganda
Ethiopia	Namibia	Zambia
Gabon	Niger	Zimbabwe
Low-income economies ($1,005 or less)		
Afghanistan	Gambia, The	Myanmar
Bangladesh	Guinea	Nepal
Benin	Guinea-Bisau	Niger
Burkina Faso	Haiti	Rwanda
Burundi	Kenya	Sierra Leone
Cambodia	Korea, Dem Rep.	Somalia
Central African Republic	Kyrgyz Republic	Tajikistan
Chad	Liberia	Tanzania
Comoros	Madagascar	Togo
Congo, Dem. Rep	Malawi	Uganda
Eritrea	Mali	Zimbabwe
Ethiopia	Mozambique	
Lower-middle-income economies ($1,006 to $3,975)		
Angola	India	São Tomé and Principe
Armenia	Iraq	Senegal
Belize	Kiribati	Solomon Islands
Bhutan	Kosovo	Sri Lanka
Bolivia	Lao PDR	Sudan
Cameroon	Lesotho	Swaziland
Cape Verde	Marshall Islands	Syrian Arab Republic
Congo, Rep.	Mauritania	Timor-Leste
Côte d'Ivoire	Micronesia, Fed. Sts.	Tonga
Djibouti	Moldova	Turkmenistan
Egypt, Arab Rep.	Mongolia	Tuvalu
El Salvador	Morocco	Ukraine
Fiji	Nicaragua	Uzbekistan
Georgia	Nigeria	Vanuatu
Ghana	Pakistan	Vietnam
Guatemala	Papua New Guinea	West Bank and Gaza
Guyana	Paraguay	Yemen, Rep.
Honduras	Philippines	Zambia
Indonesia	Samoa	
Upper-middle-income economies ($3,976 to $12,275)		
Albania	Ecuador	Namibia
Algeria	Gabon	Palau
American Samoa	Grenada	Panama
Antigua and Barbuda	Iran, Islamic Rep.	Peru
Argentina	Jamaica	Romania
Azerbaijan	Jordan	Russian Federation
Belarus	Kazakhstan	Serbia
Bosnia and Herzegovina	Latvia	Seychelles
Botswana	Lebanon	South Africa
Brazil	Libya	St. Kitts and Nevis
Bulgaria	Lithuania	St. Lucia
Chile	Macedonia, FYR	St. Vincent and the Grenadines
China	Malaysia	Suriname
Colombia	Maldives	Thailand
Costa Rica	Mauritius	Tunisia
Cuba	Mayotte	Turkey
Dominica	Mexico	Uruguay
Dominican Republic	Montenegro	Venezuela, RB

Annex 3a: Variables extracted in Stage 1 screening of titles and abstracts, and review of full text

Variables extracted during screening of full text articles in Stage 1

Duplicate

Include Health systems, including health promotion

Include Community settings

Include tracer condition/clinical intervention

Include tracer condition/other interventions

Include-Service utilisation and non-intervention (ONLY ARTICLES IN THIS GROUP IF ON THE CLINICAL TRACER CONDITIONS)

Include - query

EXCLUDED CATEGORIES OF ARTICLES (NO FURTHER EXTRACTION TO BE DONE):

- Exclude - not maternal health
- Exclude language
- Exclude - pre 2000
- Exclude - no intervention/outcome
- Exclude-Non-relevant clinical intervention(s)
- Exclude - not LMIC
- Exclude - not research

Background only (use sparingly) e.g. need to check references of an article, or is an article of much interest to the review

Query unclear (details)

Variables extracted during screening of full text articles in Stage 1

1. **EXCLUDE** on title and/or abstract, and why excluded (hierarchy approach: mark only highest applicable item on list):
   - An excluded language
   - Publication pre-2000
   - Population not maternal health
   - No intervention or outcome
   - Single clinical intervention (other than the selected tracer conditions)
   - Not LIMC
   - Not research
   - Other, specify

2. **INCLUDE**, code the topic and study design for all included studies (multiple-response question, MARK ALL APPLICABLE!)
   - **Include Interventional Topic** MARK ALL APPLICABLE RESPONSES
     - Health systems or multiple clinical interventions
     - Community-based interventions
     - Maternal malaria
     - Maternal BP/Hypertension
     - Maternal HIV/STIs
     - Antepartum postpartum haemorrhage
     - Pregnancy sepsis
   - **Include Other**
     - Service utilisation/coverage

3. **NO ABSTRACT**, title indicates article may be relevant but abstract not available

4. **QUERY**, need Full Text to decide if INCLUDE (specify reason for query).

5. **DUPLICATE**

4. **BACKGROUND** is EXCLUDED or INCLUDED on TI/AB, but need to check references of an article, or is an article of much interest to the review

Variables extracted in Stage 1 of the review from full text records

Category A: Generic codes

Variables to be extracted from full text of all articles included after screening of full text. Articles coded only as service utilisation and not one of the tracer conditions are to be coded latter

A. Generic codes, apply to all included FULL TEXT articles:

Excluded on Full text

Language not English (add details) Add language to text box if known

Service utilisation non-tracer (2 be coded later). Service utilisation studies covering tracer conditions must be coded now.

1. Country(ies) (tick all that apply) where research conducted. Tick next to name of country(ies) or type name of country(ies) in other details
2. Country(ies) of first author affiliation. Tick next to name of country(ies) or type name of country(ies) in other details
3. Study population is a PROGRESS-Plus group? PROGRESS-PLUS=Place of Residence, Race/Ethnicity, Occupation, Gender, Religion, Education, Socioeconomic Status, and Social Capital, and Plus represents additional categories such as Age, Disability and Sexual Orientation
4. Paper addresses WHO health promotion? *Tick* Yes if fits into the WHO definition of WHO Health Promotion. Note this is a wide definition involving activities within the community, for the community or with the community, including that which occurs in health service settings, or that which reports community/user involvement/empowerment/engagement. Tick unclear if unsure. Please see below for full definition of WHO health promotion.
5. Research question(s) study might answer (tick all applicable) Health systems (CODE C); Community settings*;* WHO Health promotion; Tracer conditions with single clinical intervention*;* Tracer conditions with complex/multiple interventions*;* Health service utilisation/non-intervention research; Other (details)
6. Study design, enter name of study if provided. Also coded as: Systematic Review; Review (other); Randomised controlled trial (RCT); Effectiveness evaluation including process evaluation (not RCT); Qualitative design; Formative non-intervention research; Other (details); Unclear
7. Intervention topic(s) (tick all that apply) *Emergency obstetric care; Prolonged or obstructed labour; Maternal bleeding/haemorrhage; Sepsis/infection; STIs - other than HIV; Malaria; HIV or MTCT; Hypertension/blood pressure; Induced abortion or post-abortion care; Demand side financing; Miscarriage; Male involvement; Transport schemes;; Traditional birth attendants; Maternity waiting homes; Birth and complications preparedness; Female genital mutilation; Family planning (postpartum or post abortion); Other (add details); Not applicable*
8. DIRECT intervention recipient/population (tick all that apply): Women; Family; Male partner; Community; Community health worker; Traditional birth attendant; Midwife/Nurse; Other mid-level provider (add details); Doctor/Obstetrician; Managers; Planner; Policy maker(s) (add details); Other (add details); Not applicable (add details)
9. Period mainly targeted by intervention (tick all that apply) Pregnancy; Childbirth; Post birth
10. Data collected: maternal health outcomes, service utilisation; cost/health economics; child health outcomes; other
11. Funder. Name of funder, or government funder if mentioned

Category C: Health system codes

C. Specialist health systems codes

INCLUDED CATEGORIES TO EXTRACT FROM HEALTH SYSTEM OR HEALTH PROMOTION ARTICLES

Code all articles from Full text coding that were coded as "include Health systems, include health promotion articles" or "include community setting".

1. **Developer of intervention: National NGO; International NGO; Government (add details)** give the part of government that implemented the intervention**; Research Group; Other (add details).**
2. **Main implementing agency: National NGO; International NGO; Government (add details)** give the part of government that implemented the intervention; **Research Group; Private sector; Other (add details)**
3. **Intervention delivery extent: Entire country; More than one district but not entire country (I**ncludes states); **Single district; More than one facility but not entire district; Single facility (hospital or clinic); Other (add details)** Includes community
4. **Nature of intervention: Broad system intervention beyond maternal health (**A system-level intervention directly targeting one or more of the six health system building blocks): **A maternal disease/condition-specific intervention** (A maternal disease/condition-specific intervention that is expected to have (large) system-wide effects); **Other (add details)**
5. **The intervention involves (tick all with predominant focus): Changes to health services** (Changes to health services at the organizational level which are not expected to have a system-wide effect (e.g. modification of patient flow within a health facility); **Health system-level changes** (Building blocks other than service delivery); **Change at community level** (Intervention directly involving community); **Changes beyond health system** (Changes beyond health system, e.g. micro-credit schemes); **Other (add details)**
6. **Number of building blocks: Single; Multiple; None**
7. **Type of health service or system intervention: (**Type of health system intervention (derived from Table 3 in Adam et al., 2012); **Model of service delivery (**e.g. Scaling up, Integration, Quality improvements, a. Service package, b. Health service organisation: delivery platforms, integration, (de)centralisation c. Quality assurance, adherence to protocols. d. Demand creation); **Health human resource strategy (**e.g. a. Health worker training, skills b. Skills mix, task shifting c. Employment conditions (salaries, benefits, career path, training incentives) d. Supervision e. Performance review, registration, accreditation); **Information systems (a.** Availability of information systems b. Timeliness, quality of data c. Enforcing reporting requirements d. Use of data for programme improvement); **Pharmaceuticals & medical technologies (**e.g. a. Availability of drugs and technologies b. Pricing of medicines and medical supplies c. Procurement, supply chain management d. Rational prescription and use e. Introducing/scale-up of new technologies); **Financing interventions** e.g. a. Availability of finances for health (budget allocation, fiscal space). b. User fees, insurance mechanisms. c. Provider payment / incentives. d. Service vouchers (overlap with demand creation above); **Sector reforms / Governance** e.g. Decentralisation a. Roles & responsibility, level of decision making. b. Professionalism c. Accountability (incl community participation, consumer/stakeholder involvement); **Other (add details); Not health service/system intervention, specify**

Category D: Health promotion codes

D. Specialist health promotion codes (tick all that apply – interventions could fit into a number of codes)

Maternity waiting homes: A maternity waiting home is a setting near a health facility where women can stay in the final weeks of pregnancy. Sometimes called maternity waiting village/facility

Health education (not including birth preparedness: Interventions that use health education with pregnant women, their partners/husbands, their families or with other community members to improve key maternal & new born health outcomes, including improved care practices in the home and improved use of maternal and new born health services**.** Health education must be an explicit component of the intervention. Only include counselling interventions (e.g VCT voluntary counselling and testing for HIV) where the authors have an explicit focus on an education related elements (e.g knowledge outcomes, provider training, service uptake, educational resources).

Birth and complication preparedness: Interventions that works with pregnant women, their partners and families focusing on preparations for birth and in case of complications including who will accompany to the facility, how she will get there, saving funds if needed, what materials to bring, blood donor, etc. Often emergency for after birth including for new born can be discussed

TBA's in the health services: Interventions that involve Traditional Birth Attendants (sometimes called community midwives/traditional midwives). We are particularly interested in interventions that find roles for TBAs that do not involve assisting childbirth but give them other roles to integrate them into health services.

Role of men/other community influential: Any interventions with women, men and/or community members to increase positive male, family and community involvement in supporting the women for care during pregnancy, childbirth or after birth, including care for the child after birth. Other 'community influentials' might include mother in laws, father in laws, other relatives, friends, community leaders, religious leaders who influence decisions and social norms for care during pregnancy, for childbirth and after birth

Community participation in maternal death reviews: Use of methodologies and tools such as community epidemiological surveillance, community-based death reviews, maternal and perinatal death audits, verbal autopsies, and other research on maternal and newborn health issues, where the community is considered a partner not just a source of information i.e. including the involvement of community representatives in gathering, analysing and using the information.

Community involvement other: Use for community involvement in development, delivery, quality, and evaluation of intervention, services or programmes.

Participatory learning and action cycles: Participatory Learning and Action (PLA) is a form of action research. It is a practical, adaptive research strategy that enables diverse groups and individuals to learn, work and act together in a co-operative manner, to focus on issues of joint concern, identify challenges and generate positive responses in a collaborative and democratic manner. Include any study using this approach that works with women, families or communities.

Social accountability: Social accountability can be defined as an approach towards building accountability (of healthcare providers/services/departments) that relies on civic/community/user engagement, i.e., in which it is ordinary citizens and/or civil society organizations who participate directly or indirectly in exacting accountability.

Transport schemes: Interventions that aim to reduce transport barriers women face in accessing skilled care at birth or birth in a facility. These interventions could include a) Interventions to provide non-conventional transport methods E.g. bicycle ambulance, trucks, buses, boats, ox-carts, modified tricycles with platforms, canoes, taxis, three-wheeled motorcycles and trailers. b) Interventions that provide funds to women for transport / of pay for transport for women e.g. vouchers / community emergency funds or c) Interventions organized by the health system to improve transport to for women to facilities and between facilities.

Promotion of human rights: This includes promotion of human rights, sexual rights, reproductive rights, and right to quality health care. Study should explicitly use the language or approach of 'rights'.

Companion of choice at birth: Any intervention focusing on enabling women to have a companion of choice for birth in a facility. These companions can be partners, TBAs, family members or a doula.

Respectful car: Interventions focusing on combating physical abuse; non-consented clinical care; non-confidential care, non-dignified care i.e. verbal abuse; discrimination in services; abandonment and detention in facilities. E.g. Intervention to put in curtains between beds, increase support and supervision of health care workers to improve how they treat women.

Interpersonal/Intercultural Competencies: Include papers about improving providers and services skills to interact with women including interpersonal training, efforts to understand cultural factors that affect use of care, etc.

Community health worker/Services in the community: Interventions delivered in community settings (any activities occurring outside health facilities), provided outcome described (including process/uptake outcomes), even delivery of single clinical interventions. Includes community 'micro-financing' & 'peer services'. Include interventions that use community health workers where they are mandated to deliver services in the community.

Demand side financing: Interventions to reduce financial barriers women face in accessing ANC, childbirth and post-partum, care. I.e. conditional cash transfers/vouchers/ user fee exemptions/loans and subsidies

Other health promotion activity: Falls under the broad definition of WHO health promotion activities - BUT does not address a PICO question or topic in the list above. I.e. whose objectives relate to increasing individual, family or community capacity to contribute to improved health or to increase use of maternal and new born health services.

Annex 3b: Generic variables extracted in Stage 2 from original research for all five PICOs

CODE LIST TO BE UPDATED ONCE CODE SET FINALISED

Codes of extracted variables from selected studies:

1. Inclusion criteria for study. *State the inclusion criteria for the study, or state "none".*

2. Exclusion criteria for study. *State the inclusion criteria for the study, or state "none".*

3. Name of programme/study. *Write name of programme, give full details. Detail any other publications linked to this study.*

4. Study population. *List details separately of intervention and control population, and their characteristics. Provide number enrolled; population characteristics including age of participants and proportion rural/urban, or any other key population characteristics; and population setting or context.*

5. Study design. *Describe the study design as RCT, controlled trial, cohort analytic, cose-control, cohort, interrupted time-series, qualitative, or other*

6. Intervention / exposure characteristics. *Provide detailed intervention description, year of intervention, site of intervention, intervention provider, medium of intervention, intervention intensity, level of intervention, single or multiple intervention, community activities*

7. Comparator characteristics. *Describe the comparison intervention*

8. Outcomes (in study groups or sub-groups). *Extract definition and actual outcomes for care-seeking, maternal health, newborn health, views of women, health worker or TBA outcomes, health system outcomes, community outcomes*

9. Quantitative study quality. *Detail study quality as per the EPHPP quality assessment tool (Annex 4)*

10. Qualitative study quality. *Detail study quality as per the qualitative study quality assessment tool (Annex 4)*

11. Factors influencing the effectiveness of the strategy. *Copy any text about barriers/enablers to the intervention, factors influencing its delivery.*

Annex 3c: Variables extracted from existing systematic reviews in Stage 2

List of variables to extract into Microsoft Excel Sheet from existing systematic reviews on the WHO PICO questions

Variable List

1. Title and authors

2. Review objective(s)

3. Inclusion criteria of review. List all criteria used to decide on inclusion of studies in the review

4. Exclusion criteria of review. List all criteria used to exclude studies from the review

5. Studies included in review. Provide information on the number of studies included in the SR and number of participants in the studies

6. Population. Describe the Population of the PICO for the review

7. Intervention. Describe the Intervention of the PICO for the review

8. Comparator. Describe the Comparator of the PICO for the review

9. Health seeking outcomes extracted

10. Maternal Health outcomes extracted

11. Child health outcomes extracted (including newborn health outcomes)

12. Other outcomes extracted. List all other outcomes presented in the review, or extracted as reported in correspondence with the review authors

13. Data extracted on values and preferences. Indicate "yes" if data extracted on this, or "no" if not, describe the variables extracted on this

14. Study quality assessed. Describe if quality of included studies assessed, how assessed and name of quality assessment tools used if applicable

15. Comments

16. Any additional components required to complete the review. Note here any additional variables that need to be extracted from original studies

17. Quality of systematic review methods. See Annex 4. Apply the AMSTAR (assessment of multiple systematic reviews) measurement tool to assess the methodological quality of systematic reviews on the topic

Annex 4: Quality Assessment tools

Quality assessment of systematic reviews

AMSTAR (assessment of multiple systematic reviews); a measurement tool to assess the methodological quality of systematic reviews (4, 8)

For all criteria enter: Yes; No; Can't answer; or Not applicable. A Yes replay gives a score of 1. Sum the scores to give a total figure.

AMSTAR Criteria	Additional Notes.
**1 The research question and inclusion criteria were established before the conduct of the review?**	Note: Need to refer to a protocol, ethics approval, or pre-determined/a priori published research objectives to score a "yes."
**2 Was there duplicate study selection and data extraction?**	There should be at least two independent data extractors and a consensus procedure for disagreements should be in place. Note: 2 people do study selection, 2 people do data extraction, consensus process or one person checks the other's work.
**3 Was a comprehensive literature search performed?**	At least two electronic sources should be searched. The report must include years and databases used (e.g., Central, EMBASE, and MEDLINE). Key words and/or MESH terms must be stated and where feasible the search strategy should be provided. All searches should be supplemented by consulting current contents, reviews, textbooks, specialized registers, or experts in the particular field of study, and by reviewing the references in the studies found. Note: If at least 2 sources + one supplementary strategy used, select "yes" (Cochrane register/Central counts as 2 sources; a grey literature search counts as supplementary).
**4 Was the status of publication (i.e. grey literature) used as an inclusion criterion?**	The authors should state that they searched for reports regardless of their publication type. The authors should state whether or not they excluded any reports (from the systematic review), based on their publication status, language etc. Note: If review indicates that there was a search for "grey literature" or "unpublished literature," indicate "yes." SINGLE database, dissertations, conference proceedings, and trial registries are all considered grey for this purpose. If searching a source that contains both grey and non-grey, must specify that they were searching for grey/unpublished lit.
**5 Was a list of studies (included and excluded) provided?**	
**6 Were the characteristics of the included studies provided?**	In an aggregated form such as a table, data from the original studies should be provided on the participants, interventions and outcomes. The ranges of characteristics in all the studies analyzed e.g., age, race, sex, relevant socioeconomic data, disease status, duration, severity, or other diseases should be reported. Note: Acceptable if not in table format as long as they are described as above.
**7 Was the scientific quality of the included studies assessed and documented?**	'A priori' methods of assessment should be provided (e.g., for effectiveness studies if the author(s) chose to include only randomized, double-blind, placebo controlled studies, or allocation concealment as inclusion criteria); for other types of studies alternative items will be relevant. Note: Can include use of a quality scoring tool or checklist, e.g., Jadad scale, risk of bias, sensitivity analysis, etc., or a description of quality items, with some kind of result for EACH study ("low" or "high" is fine, as long as it is clear which studies scored "low" and which scored "high"; a summary score/range for all studies is not acceptable).
**8 Was the scientific quality of the included studies used appropriately in formulating conclusions?**	The results of the methodological rigor and scientific quality should be considered in the analysis and the conclusions of the review, and explicitly stated in formulating recommendations. Note: Might say something such as "the results should be interpreted with caution due to poor quality of included studies." Cannot score "yes" for this question if scored "no" for question 7.
**9 Were the methods used to combine the findings of studies appropriate?**	For the pooled results, a test should be done to ensure the studies were combinable, to assess their homogeneity (i.e., Chi-squared test for homogeneity, I2). If heterogeneity exists a random effects model should be used and/or the clinical appropriateness of combining should be taken into consideration (i.e., is it sensible to combine?). Note: Indicate "yes" if they mention or describe heterogeneity, i.e., if they explain that they cannot pool because of heterogeneity/variability between interventions.
**10 Was the likelihood of**	An assessment of publication bias should include a combination of graphical aids (e.g.,

**publication bias assessed?**	funnel plot, other available tests) and/or statistical tests (e.g., Egger regression test). Note: If no test values or funnel plot included, score “no”. Score “yes” if mentions that publication bias could not be assessed because there were fewer than 10 included studies.
**11 Was the conflict of interest included?**	
**AMSTAR Total Score**	Each item scores 1 for yes Sum the scores, out of 11

Quality assessment of quantitative studies

Quality assessment tool for quantitative studies; Effective Public Health Practice Project(9-11)

QUALITY ASSESSMENT TOOL FOR QUANTITATIVE STUDIES

EPHPP
Effective Public Health Practice Project

COMPONENT RATINGS

A) SELECTION BIAS

(Q1) Are the individuals selected to participate in the study likely to be representative of the target population?

1 Very likely
2 Somewhat likely
3 Not likely
4 Can't tell

(Q2) What percentage of selected individuals agreed to participate?

1 80 - 100% agreement
2 60 – 79% agreement
3 less than 60% agreement
4 Not applicable
5 Can't tell

RATE THIS SECTION	STRONG	MODERATE	WEAK
See dictionary	1	2	3

B) STUDY DESIGN

Indicate the study design

1 Randomized controlled trial
2 Controlled clinical trial
3 Cohort analytic (two group pre + post)
4 Case-control
5 Cohort (one group pre + post (before and after))
6 Interrupted time series
7 Other specify ______________________________
8 Can't tell

Was the study described as randomized? If NO, go to Component C.

No Yes

If Yes, was the method of randomization described? (See dictionary)

No Yes

If Yes, was the method appropriate? (See dictionary)

No Yes

RATE THIS SECTION	STRONG	MODERATE	WEAK
**See dictionary**	1	2	3

C) CONFOUNDERS

(Q1) **Were there important differences between groups prior to the intervention?**

1 Yes
2 No
3 Can't tell

The following are examples of confounders:

1 Race
2 Sex
3 Marital status/family
4 Age
5 SES (income or class)
6 Education
7 Health status
8 Pre-intervention score on outcome measure

(Q2) **If yes, indicate the percentage of relevant confounders that were controlled (either in the design (e.g. stratification, matching) or analysis)?**

1 80 – 100% (most)
2 60 – 79% (some)
3 Less than 60% (few or none)
4 Can't Tell

RATE THIS SECTION	STRONG	MODERATE	WEAK
See dictionary	1	2	3

D) BLINDING

(Q1) **Was (were) the outcome assessor(s) aware of the intervention or exposure status of participants?**

1 Yes
2 No
3 Can't tell

(Q2) **Were the study participants aware of the research question?**

1 Yes
2 No
3 Can't tell

RATE THIS SECTION	STRONG	MODERATE	WEAK
See dictionary	1	2	3

E) DATA COLLECTION METHODS

(Q1) **Were data collection tools shown to be valid?**

1 Yes
2 No
3 Can't tell

(Q2) **Were data collection tools shown to be reliable?**

1 Yes
2 No
3 Can't tell

RATE THIS SECTION	STRONG	MODERATE	WEAK
See dictionary	1	2	3

F) WITHDRAWALS AND DROP-OUTS

(Q1) **Were withdrawals and drop-outs reported in terms of numbers and/or reasons per group?**

1 Yes
2 No
3 Can't tell
4 Not Applicable (i.e. one time surveys or interviews)

(Q2) **Indicate the percentage of participants completing the study. (If the percentage differs by groups, record the lowest).**

1 80 -100%
2 60 - 79%
3 less than 60%
4 Can't tell
5 Not Applicable (i.e. Retrospective case-control)

RATE THIS SECTION	STRONG	MODERATE	WEAK	
See dictionary	1	2	3	Not Applicable

G) INTERVENTION INTEGRITY

(Q1) **What percentage of participants received the allocated intervention or exposure of interest?**

1 80 -100%
2 60 - 79%
3 less than 60%
4 Can't tell

(Q2) **Was the consistency of the intervention measured?**

1 Yes
2 No
3 Can't tell

(Q3) **Is it likely that subjects received an unintended intervention (contamination or co-intervention) that may influence the results?**

4 Yes
5 No
6 Can't tell

H) ANALYSES

(Q1) **Indicate the unit of allocation (circle one)**

community organization/institution practice/office individual

(Q2) **Indicate the unit of analysis (circle one)**

community organization/institution practice/office individual

(Q3) **Are the statistical methods appropriate for the study design?**

1 Yes
2 No
3 Can't tell

(Q4) **Is the analysis performed by intervention allocation status (i.e. intention to treat) rather than the actual intervention received?**

1 Yes
2 No
3 Can't tell

GLOBAL RATING

COMPONENT RATINGS

Please transcribe the information from the gray boxes on pages 1-4 onto this page. See dictionary on how to rate this section.

A	SELECTION BIAS	STRONG	MODERATE	WEAK	
		1	2	3	
B	STUDY DESIGN	STRONG	MODERATE	WEAK	
		1	2	3	
C	CONFOUNDERS	STRONG	MODERATE	WEAK	
		1	2	3	
D	BLINDING	STRONG	MODERATE	WEAK	
		1	2	3	
E	DATA COLLECTION METHOD	STRONG	MODERATE	WEAK	
		1	2	3	
F	WITHDRAWALS AND DROPOUTS	STRONG	MODERATE	WEAK	
		1	2	3	Not Applicable

GLOBAL RATING FOR THIS PAPER (circle one):

1	STRONG	(no WEAK ratings)
2	MODERATE	(one WEAK rating)
3	WEAK	(two or more WEAK ratings)

With both reviewers discussing the ratings:

Is there a discrepancy between the two reviewers with respect to the component (A-F) ratings?

No Yes

If yes, indicate the reason for the discrepancy

1 Oversight
2 Differences in interpretation of criteria
3 Differences in interpretation of study

Final decision of both reviewers (circle one):

1 STRONG
2 MODERATE
3 WEAK

Quality assessment of qualitative studies

Appraising the quality of qualitative research: Summary criteria for appraising qualitative research studies (5)

Stages	Essential criteria	Specific prompts
Scope and purpose	Clear statement of, and rationale for, research question, aims or purposes	Clarity of focus demonstrated Explicit purpose given, such as descriptive/explanatory intent, theory building, hypothesis testing Link between research and existing knowledge demonstrated
	Study thoroughly contextualised by existing literature	Evidence of systematic approach to literature review, location of literature to contextualise the findings, or both
Design	Method/design apparent, and consistent with research intent	Rationale given for use of qualitative design Discussion of epistemological/ontological grounding Rationale explored for specific qualitative method (e.g. ethnography, grounded theory, phenomenology) Discussion of why particular method chosen is most appropriate/sensitive/relevant for research question/aims Setting appropriate
	Data collection strategy apparent and appropriate	Were data collection methods appropriate for type of data required and for specific qualitative method? Were they likely to capture the complexity/diversity of experience and illuminate context in sufficient detail? Was triangulation of data sources used if appropriate?
Sampling strategy	Sample and sampling method appropriate	Selection criteria detailed, and description of how sampling was undertaken Justification for sampling strategy given Thickness of description likely to be achieved from sampling Any disparity between planned and actual sample explained
Analysis	Analytic approach appropriate	Approach made explicit (e.g. Thematic distillation, constant comparative method, grounded theory) Was it appropriate for the qualitative method chosen? Was data managed by software package or by hand and why? Discussion of how coding systems/conceptual frameworks evolved How was context of data retained during analysis Evidence that the subjective meanings of participants were portrayed Evidence of more than one researcher involved in stages if appropriate to epistemological/theoretical stance Did research participants have any involvement in analysis (e.g. member checking) Evidence provided that data reached saturation or discussion/rationale if it did not Evidence that deviant data was sought, or discussion/ rationale if it was not
Interpretation	Context described and taken account of in interpretation	Description of social/physical and interpersonal contexts of data collection Evidence that researcher spent time 'dwelling with the data', interrogating it for competing/alternative explanations of phenomena
	Clear audit trail given	Sufficient discussion of research processes such that others can follow 'decision trail'
	Data used to support interpretation	Extensive use of field notes entries/verbatim interview quotes in discussion of findings Clear exposition of how interpretation led to conclusions
Reflexivity	Researcher reflexivity demonstrated	Discussion of relationship between researcher and participants during fieldwork Demonstration of researcher's influence on stages of research process Evidence of self-awareness/insight Documentation of effects of the research on researcher Evidence of how problems/complications met were dealt with.

Ethical dimensions	Demonstration of sensitivity to ethical concerns	Ethical committee approval granted Clear commitment to integrity, honesty, transparency, equality and mutual respect in relationships with participants Evidence of fair dealing with all research participants Recording of dilemmas met and how resolved in relation to ethical issues Documentation of how autonomy, consent, confidentiality, anonymity were managed
Relevance and transferability	Relevance and transferability evident	Sufficient evidence for typicality specificity to be assessed Analysis interwoven with existing theories and other relevant explanatory literature drawn from similar settings and studies Discussion of how explanatory propositions/emergent theory may fit other contexts Limitations/weaknesses of study clearly outlined Clearly resonates with other knowledge and experience Results/conclusions obviously supported by evidence Interpretation plausible and 'makes sense' Provides new insights and increases understanding Significance for current policy and practice outlined Assessment of value/empowerment for participants Outlines further directions for investigation. Comment on whether aims/purposes of research were achieved

Armijo-Olivo, S., C. R. Stiles, et al. (2012). "Assessment of study quality for systematic reviews: a comparison of the Cochrane Collaboration Risk of Bias Tool and the Effective Public Health Practice Project Quality Assessment Tool: methodological research." J Eval Clin Pract **18**(1): 12-18.

Effective Public Health Practice Project "Quality assessment tool for quantitative studies." http://www.ephpp.ca/tools.html

Shea, B. J., L. M. Bouter, et al. (2007). "External validation of a measurement tool to assess systematic reviews (AMSTAR)." PLoS ONE **2**(12): e1350.

Shea, B. J., J. M. Grimshaw, et al. (2007). "Development of AMSTAR: a measurement tool to assess the methodological quality of systematic reviews." BMC Med Res Methodol **7**: 10.

Thomas, B. H., D. Ciliska, et al. (2004). "A process for systematically reviewing the literature: providing the research evidence for public health nursing interventions." Worldviews Evid Based Nurs **1**(3): 176-184.

Walsh, D. and S. Downe (2006). "Appraising the quality of qualitative research." Midwifery **22**(2): 108-119.

References

Equity extension of PRISMA guidelines

Website references

EPPI-Reviewer 4 web based systematic review software
http://eppi.ioe.ac.uk/cms/Default.aspx?alias=eppi.ioe.ac.uk/cms/er4

EPPI-Centre Health Promotion and Public Health Reviews Facility
http://eppi.ioe.ac.uk/cms/Default.aspx?tabid=73

EPPI Centre website and list of all systematic reviews:
http://eppi.ioe.ac.uk/cms/Default.aspx?tabid=62

EPPI Centre Teaching and Learning

http://eppi.ioe.ac.uk/cms/Default.aspx?tabid=168

Publications

Methods for the thematic synthesis of qualitative research in systematic reviews
http://www.biomedcentral.com/1471-2288/8/45

BMJ paper example of equity review – healthy eating mixed methods
http://es.scribd.com/doc/40588135/Integrating-Qualitative-Research-with-Trials-in-systematic-reviews

Chapter on mixed methods synthesis
https://portal.ioe.ac.uk/http/onlinelibrary.wiley.com/doi/10.1002/9781119959847.ch6/summary

Map of inequalities in young people's health example of equity review
http://eppi.ioe.ac.uk/cms/LinkClick.aspx?fileticket=mVu6mYHcwbc%3d&tabid=2410&mid=4471

Reflections on developing and using PROGRESS-Plus

http://equity.cochrane.org/Files/Equity_Update_Vol2_Issue1.pdf

1. van Lonkhuijzen L, Stekelenburg J, van Roosmalen J. Maternity waiting facilities for improving maternal and neonatal outcome in low-resource countries. Cochrane Database of Systematic Reviews. 2012(10. Art. No.: CD006759. DOI:10.1002/14651858.CD006759.pub3.).
2. Kang D, Wu Y, Hu D, Hong Q, Wang J, Zhang X. Reliability and External Validity of AMSTAR in Assessing Quality of TCM Systematic Reviews. Evid Based Complement Alternat Med. 2012;2012:732195.
3. Shea BJ, Hamel C, Wells GA, Bouter LM, Kristjansson E, Grimshaw J, et al. AMSTAR is a reliable and valid measurement tool to assess the methodological quality of systematic reviews. J Clin Epidemiol. 2009 Oct;62(10):1013-20.
4. Shea BJ, Grimshaw JM, Wells GA, Boers M, Andersson N, Hamel C, et al. Development of AMSTAR: a measurement tool to assess the methodological quality of systematic reviews. BMC Med Res Methodol. 2007;7:10.
5. Walsh D, Downe S. Appraising the quality of qualitative research. Midwifery. 2006 Jun;22(2):108-19.

6. Egger M, Davey Smith G, Altman DG. Systematic Reviews in Health Care: Meta-Analysis in Context, Second Edition 2008.
7. The World Bank. Country and Lending Groups: Low- and middle-income economies2012: Available from: http://data.worldbank.org/about/country-classifications/country-and-lending-groups.
8. Shea BJ, Bouter LM, Peterson J, Boers M, Andersson N, Ortiz Z, et al. External validation of a measurement tool to assess systematic reviews (AMSTAR). PLoS ONE. 2007;2(12):e1350.
9. Effective Public Health Practice Project. Quality assessment tool for quantitative studies: Available from: http://www.ephpp.ca/tools.html.
10. Thomas BH, Ciliska D, Dobbins M, Micucci S. A process for systematically reviewing the literature: providing the research evidence for public health nursing interventions. Worldviews Evid Based Nurs. 2004;1(3):176-84.
11. Armijo-Olivo S, Stiles CR, Hagen NA, Biondo PD, Cummings GG. Assessment of study quality for systematic reviews: a comparison of the Cochrane Collaboration Risk of Bias Tool and the Effective Public Health Practice Project Quality Assessment Tool: methodological research. J Eval Clin Pract. 2012 Feb;18(1):12-8.
