# Supplementary material for: Involving men to improve maternal and newborn health: A systematic review of the effectiveness of interventions
Source: PLoS One. 2018 Jan 25;13(1):e0191620. doi: 10.1371/journal.pone.0191620 (PMC5784936; doi:10.1371/journal.pone.0191620)
Supplement: S3 File — (PDF) [file pone.0191620.s008.pdf]

Addendum: Supplementary review for PICO: Strategies for male involvement

Background

This addendum complements the Guiding document and provides the additional PICO specific methods and information.

As described in section 2.1 of the Guidance document, the following steps will be taken for this PICO and detailed below.

Box 1: Steps used for this PICO and other PICO questions in the review.

1. Specify the P I C O for the systematic review;
2. Identification and analysis of existing systematic reviews;
3. Define eligibility criteria for inclusion and exclusion of articles for each PICO question;
4. Define the variables for data extraction for each PICO review. The extracted variables consist of a generic code set and a PICO specific code set;
5. Compile the PICO specific Addendum. This encompasses the PICO definition; review methods; inclusion and exclusion criteria for the PICO; specific variables to extract for the review;
6. Obtain approval from WHO and representatives of the GDG for the generic guidance and for each Addendum;
7. Identify potentially eligible studies from the various sources, including the Stage 1 review and additional stage 2 strategies;
8. Obtain full text documents and upload these into EPPI reviewer 4;
9. Determine eligibility of full-text records and extract predefined data extraction variables;
10. Quality assessment of eligible systematic reviews (AMSTAR criteria), quantitative studies (EPHPP Quality assessment tool) and qualitative studies (Walsh 2006 appraisal tool; Annex 4 of Guiding document)
11. Perform quality checks on data extraction;
12. Present relevant information in tables to enable grading and analysis of the evidence; and
13. Analysis and presentation of Evidence Profile tables.

1. Specify the P I C O for the systematic review

What interventions employed with women, men, communities and community leaders to increase male involvement have been effective in increasing care-seeking behaviour during pregnancy, for child birth and after birth for the woman and newborn and in improving key maternal and newborn health outcomes?

Box 2: PICO for the review of strategies for male involvement.

**Population**	Women, men, community leaders, communities, and health workers in low and middle income countries

**Intervention**	Interventions to increase male involvement
**Comparator**	No intervention for male involvement or different interventions for male involvement
**Critical Outcome 1**	Birth with a skilled attendant; Birth in a facility
**Critical Outcome 2**	Care with a skilled attendant or facility in case of maternal or newborn complications or illness
**Critical Outcome 3**	Use of antenatal care (1 or 4 visits)
**Critical Outcome 4**	Postpartum care visit mother
**Critical Outcome 5**	Uptake of essential MCH package or specific interventions in the package, such as iron supplementation
**Critical Outcome 6**	Maternal nutrition; and newborn nutritional intake and status, including breastfeeding initiation;
**Critical Outcome 7**	Birth and complications preparedness
**Important outcome 1**	Maternal mortality and morbidity
**Important outcome 2**	Neonatal mortality
**Important outcome 3**	Perinatal mortality

Note that the full list of outcomes and other variables to be extracted from eligible articles is listed in section 4."

2. Identification and analysis of existing systematic reviews and gaps analysis

No recent systematic reviews cover this topic in sufficient detail or applied adequate review methods. One systematic review (Davis et al. 2012) on this topic has been identified and we will search the references list for possible studies to include in the review.

3. Define eligibility criteria for inclusion and exclusion of articles for each PICO question

The review includes all study designs with a point of comparison. Only original studies will be included, systematic reviews only are used for assisting to locate studies.

Studies marked as query will be discussed between the review team, who will then decide if the article is to be included.

Inclusion criteria for the supplementary review

a. Intervention of interest. Any strategy employed with women, men, community leaders, communities or health workers to increase male involvement.
b. Outcomes. At least one of the study outcomes detailed in Box 2 above.
c. Comparator group. The *comparison* group involved no strategy for male involvement, or a different strategy.
d. Study setting. Studies in low- and middle-income countries will be included in this review.
e. Types of studies. All study designs will be included provided they report empirical data on an assessment of the outcome of an intervention. For quantitative studies, the study outcome reported among women having male involvement must be compared with the outcome in any comparison group. For qualitative studies, attitudes or experiences related to the intervention are included.

f. Language. Arabic, English, French, Japanese, Portuguese and Spanish language studies will be included.
g. Dates of study publication Articles between 2000 and 2012 that were identified in the MASCOT/Wotro review will be included, as well as studies prior to 2000 that are included or referenced in eligible systematic reviews or mapping on the topic.

Exclusion criteria for the supplementary review

In hierarchy order (mark the first item that excludes the study):

a. Duplicate article. Publications of the same studies. Previous versions of an updated systematic review are considered duplicates.
b. Not relevant intervention. A male involvement intervention has to have been implemented, if a study only examined the recommended or intended involvement of men, this was excluded. Excluded are also studies where male involvement is solely for STI and HIV prevention and treatment including PMTCT, and HIV counselling.
c. No relevant outcome: Studies which do not report effects of the intervention on an outcome stated in Box 2 above. Studies reporting only outcomes outside of pregnancy, childbirth or the postpartum period up to one year post-childbirth will not be extracted.
d. Not empirical research: Paper includes only policy discussion, descriptions of government policies, editorials, or an opinion on a topic.
e. Conference abstract or books are excluded as these are not indexed in databases used in this review making it difficult to perform a replicable search. Further, extraction from such work requires specialised methods beyond the scope of this review.

4. Define the variables for data extraction for each PICO review. The extracted variables consist of a generic code set and a PICO specific code set

The first task of the reviewed is to indicate if the study meets all inclusion and exclusion criteria as set out for this review. For excluded studies, the reason for exclusion is provided.

Two boxes are presented below for the codes for this review. Box 3 lists the variables to extract describing the intervention and the study population. Box 4 shows the variables to extract on the outcomes and other experiences related to the intervention. Quality of the study will be assessed for all included studies (both quantitative and qualitative studies; see Annex 4). The code set consists of generic codes applied to all studies in all reviews, as well as some PICO-specific codes. Definitions of each code are provided in EPPI reviewer.

Box 3: Codes for data extraction on intervention and study population.

1. Inclusion criteria for study *State the inclusion criteria for the study, or state "none"*

2. Exclusion criteria for study *State the inclusion criteria for the study, or state "none"*

3. Primary study objective State the primary or main objective of the study

4. Name of programme/study *Write name of programme, if provided, give full details. Detail any other publications linked to this study*

5. Study population *Give the countr(ies) of the study and the years the intervention took*

place. List details separately of intervention and control population, and their characteristics. Provide number enrolled; population characteristics including age of participants and proportion rural/urban, and education level of women; and population setting or context (for example, give the distance to facility for women if reported, the level of health service where study is set).

6. Selection criteria for men and/or women to receive male involvement intervention
Specify the criteria used for selecting which men and/or women were to receive the male involvement intervention

7. Study design *Describe the study design as RCT, controlled trial, cohort analytic, case-control, cohort, interrupted time-series, qualitative, or other. If randomised trial, extract additional information (method of randomisation, and if this is an appropriate method)*

8. Strategy for male involvement and intervention characteristics *(Copy all text describing the strategy for male involvement and intervention characteristics; ensure comprehensive extraction of all intervention characteristics). Strategies for male involvement could include e.g. community-based strategies, workplace strategies, group education, mass media campaigns and clinic-based initiatives. Intervention characteristics should include information on mode of delivery of the intervention (e.g. IEC materials, one-on-one counselling, community event, radio messages etc), who delivered (e.g. peer educator, senior community member etc), and where delivered (e.g. during football matches, at antenatal clinic).*

9. Single or multiple intervention *(if multiple intervention, extract the interventions provided in addition to the strategies for male involvement)*

10. Intervention intensity *(state the amount of exposure each participant was intended to receive. Write in the exact intended intervention duration and number of visits or exposures).*

11. Comparator characteristics *Describe the comparison intervention, by copying from the paper what the comparator received. Also categorise the comparator intervention as: limited intervention; normal standard care; no intervention; no comparator; other, specify. If there are multiple comparator groups tick other and explain what each of the comparators received.*

The reviewer must copy verbatim (free text) the definition of the outcome used in the study. In addition, extract the outcome and all information about this, e.g. confidence intervals and *P* values.

For complex interventions (where a male involvement intervention was one of several interventions provided), we excluded effects of the intervention on study outcomes if the link between the intervention and the outcome was implausible.

Extract information on outcomes for the whole study population, as well as outcomes disaggregated by age, if provided.

Box 4. Codes for data extraction on the study outcomes and experiences of study participants.

1. Outcomes (in study groups and if disaggregated into age groups).

- *Care seeking outcomes:* birth with a skilled attendant; birth in a facility; care with a

skilled attendant or facility in case of maternal or newborn complications or illness; use of antenatal care; postnatal maternal and newborn clinic attendance; routine child health clinic attendance; care-seeking for childhood illness; Family planning and contraceptive use in postpartum period up to 1 year postpartum;

- *Improved care practices:* Birth and complications preparedness; Maternal and child nutritional intake and status, including breastfeeding initiation, and exclusive breastfeeding uptake and continuation; Family planning and contraceptive use in the postpartum period; Uptake of essential MCH package or specific interventions in the package, such as iron supplementation;
- *Maternal health outcomes comprising the following categories*: maternal deaths; maternal morbidity; mental health outcomes; prolonged or obstructed labour*;*
- *Neonatal health outcomes:* stillbirths, live births, perinatal mortality, neonatal mortality; neonatal morbidity (such as admission to special care); Postnatal care visit newborn;
- *Communication outcomes:* Improved couple communication and joint decision-making for maternal, newborn and child health; changes in women's decision-making, autonomy in making informed choices and decisions;
- *Support outcomes:* Improved support (emotional, financial, or other) for women during pregnancy, childbirth and postpartum;
- *Coverage and level of male involvement (proportion of women accompanied by male partner during antenatal, delivery and post delivery; extent and level of male involvement);*
- *Cost data, including cost-effectiveness and costs for health system or patients (e.g. payments for transport and loss of income);*

2. Values and preferences. Information on values and preferences related to the intervention strategy (including acceptability and follow-up rates) and male involvement itself, both positive and negative attributes, is recorded. Measures of intimate partner violence and other potential harms, including reduced autonomy of women related to male involvement interventions will be noted. Values and preferences could be those of men, women and health workers. Qualitative data about how men want to be involved is carefully documented.

3. Factors influencing the effectiveness of the strategy. *Copy any text about barriers/enablers to the intervention, factors influencing its delivery and effectiveness.*

Satisfaction with the strategies for male involvement as well as data related to the male involvement itself should be extracted in detail.

5. Compile the PICO specific Addendum. This encompasses the PICO definition; review methods; inclusion and exclusion criteria for the PICO; specific variables to extract for the review

This document embodies the specified Addendum and encompasses the PICO definition, review methods, inclusion and exclusion criteria, and PICO specific variables to extract where different or additional from the Guiding document.

6. Obtain approval from WHO and representatives of the GDG for each Addendum

Written approval is being requested.

7. Identify potentially eligible studies from the various sources, including the Stage 1 review and additional stage 2 strategies

For this PICO, articles will be screened from the following sources:

a. Any studies included in the existing systematic reviews, as well as studies they list and describe, but were excluded by that review;
b. The Mascot/Wotro articles coded as "Role of men/other community influential". This was defined as: "Any interventions with women, men and/or community members to increase positive male, family and community involvement in supporting the women for care during pregnancy, childbirth or after birth, including care for the child after birth. Other 'community influentials' might include mother in laws, father in laws, other relatives, friends, community leaders, religious leaders who influence decisions and social norms for care during pregnancy, for childbirth and after birth"";
c. References within full text articles will be screened and those that are suggestive of a male involvement intervention will be retrieved.

8. Obtain full text documents and upload these into EPPI reviewer 4

All full-text documents accessible to the reviewer will be uploaded and made accessible in EPPI reviewer 4.

9. Determine eligibility of full-text records and extract predefined data extraction variables

Determination of eligibility of all full text records will be performed by Stanley Luchters and Matthew Chersich. Any dispute will be resolved by Anayda Portela.

10. Quality assessment of eligible systematic reviews (AMSTAR criteria), quantitative studies (EPHPP Quality assessment tool) and qualitative studies (Walsh 2006 appraisal tool; Annex 4)

Quality assessments will be done on all included studies by the review team. All quality assessment will be done in accordance with the Guiding document.

11. Perform quality checks on data extraction

Quality checks on data extraction will be performed for at least 50% of the eligible studies.

12. Present relevant information in tables to enable grading and analysis of the evidence

The data extracted will be assessed to determine: the proportion of studies with the outcomes of interest; the outcomes reported in included studies; and the conclusions that can be drawn about the effect of maternity waiting homes on care-seeking during labour and birth. Quality of the included studies will be summed. A flow chart of articles will be developed, demonstrating the number of included articles and reasons for exclusions. All findings will be presented in results text. Results will be tabulated into three tables,

specifically: Table 1 Characteristics of population and intervention of included studies; Table 2 Outcomes of included studies; Table 3 Quality of included studies.

13. Analysis and presentation of Evidence Profile tables.

As per Guiding document.

References

Davis J, Luchters S, Holmes W (2012) "Men and maternal and newborn health: Benefits, harms, challenges and potential strategies for engaging men." Melbourne: Burnet Institute on behalf of Compass: Women's and Children's Health Knowledge Hub. http://www.wchknowledgehub.com.au/sites/default/files/BP_Davis_FEB13.pdf
